# Supplementary material for: Tumor‐associated macrophages‐educated reparative macrophages promote diabetic wound healing
Source: EMBO Mol Med. 2022 Dec 21;15(2):e16671. doi: 10.15252/emmm.202216671 (PMC9906426; doi:10.15252/emmm.202216671)
Supplement: Supplementary file 1 — Appendix [file EMMM-15-e16671-s002.pdf]

APPENDIX

Title: Tumor-associated macrophages-educated reparative macrophages promote diabetic wound healing

Authors: Ruoyu Mu [1], Zhe Zhang [1], Congwei Han [1,2], Yiming Niu [1,2], Zhen Xing [2], Zhencheng Liao [1], Jinzhi Xu [2], Ningyi Shao [3], Guokai Chen [3], Junfeng Zhang [2], Lei Dong [2,*], Chunming Wang [1,4,5,*]

Affiliations:

[1] Institute of Chinese Medical Sciences, University of Macau, Macau SAR

[2] School of Life Sciences, Nanjing University, Nanjing, China

[3] Department of Biomedical Sciences, Faculty of Health Sciences, University of Macau, Macau SAR

[4] Zhuhai UM Science & Technology Research Institute, Hengqin Guangdong-Macau In-Depth Cooperation Zone, China

[5] Department of Pharmaceutical Sciences, Faculty of Health Sciences, University of Macau, Macau SAR

* Correspondence should be addressed to:

C. Wang (cmwang@umac.mo) or L. Dong (leidong@nju.edu.cn)

Table of contents

- **Appendix Figure S1 - Preparation of TAMEMs from primary murine macrophages.**
- **Appendix Figure S2 - Preparative tasks for TAMEMs transplantation.**
- **Appendix Figure S3 - Analysis of the phenotypes of host macrophages in diabetic mice.**
- **Appendix Figure S4 - Immunofluorescence staining of M1 and M2 and schematic diagram of the vascular perfusion experiment.**
- **Appendix Figure S5 - Supplementary datas about TAMEMs^{C}.**
- **Appendix Figure S6 - Directly applying the cocktail cannot replace delivering live TAMEMs to heal the diabetic wounds.**
- **Appendix Table S1 - Primer sequences used for RT-qPCR.**
- **Appendix Table S2 - The antibodies used in this study.**
- **Appendix Table S3 - The recombinant proteins used in this study.**

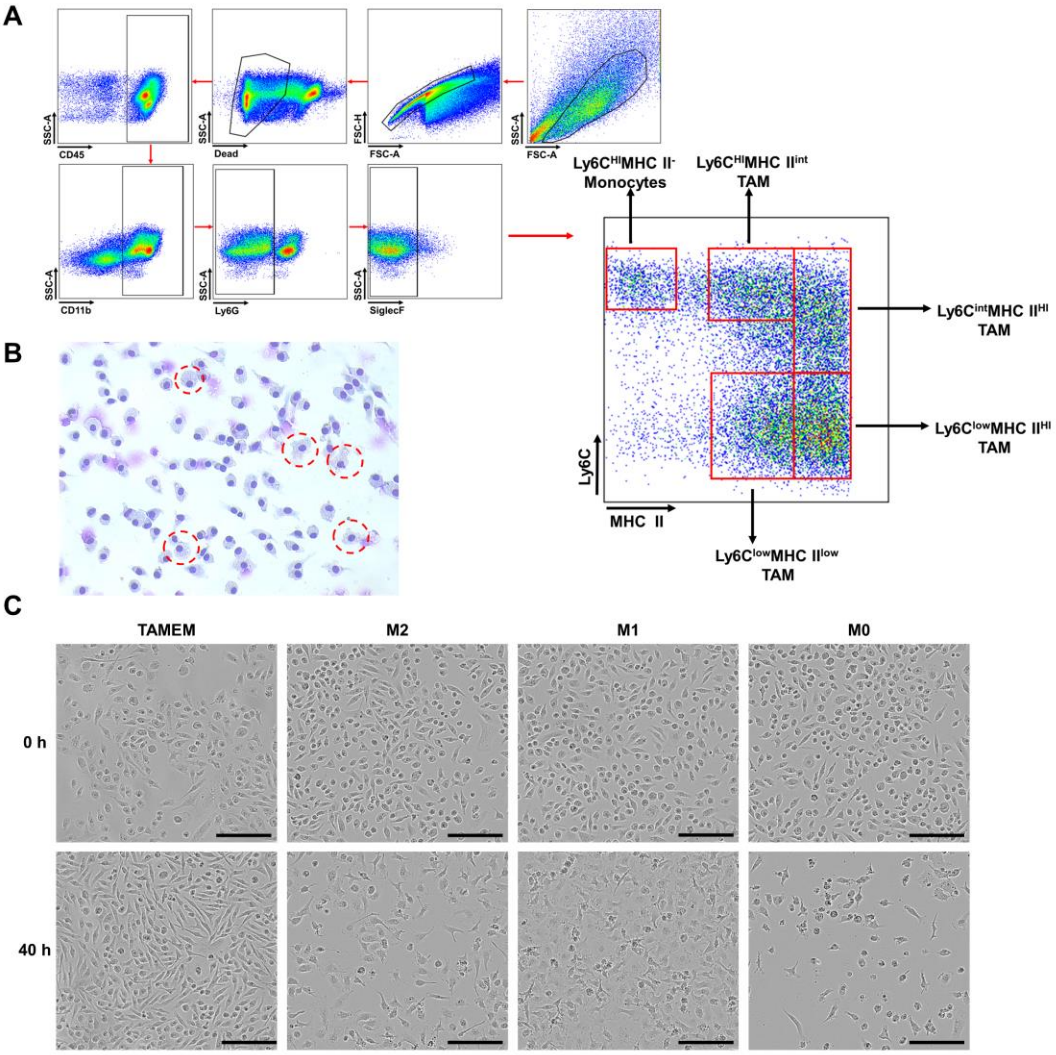

Appendix Figure S1 - Preparation of TAMEMs from primary murine macrophages.

A Gating strategy to select single, live, $CD45^+$, $CD11b^+$, $Ly6G^-$, Siglec F^- cells and clustered by Ly6C and MHC II.

B Wright-Giemsa staining (morphology) for TAMs on day 15 after isolation (the cells in the red circles are representative macrophages).

C Representative pictures of two time points of the real-time image record using IncuCyte system to assess the proliferation and morphological changes in TAMEMs and others (scale bar: 100 µm; *n*=3, biological replicates).

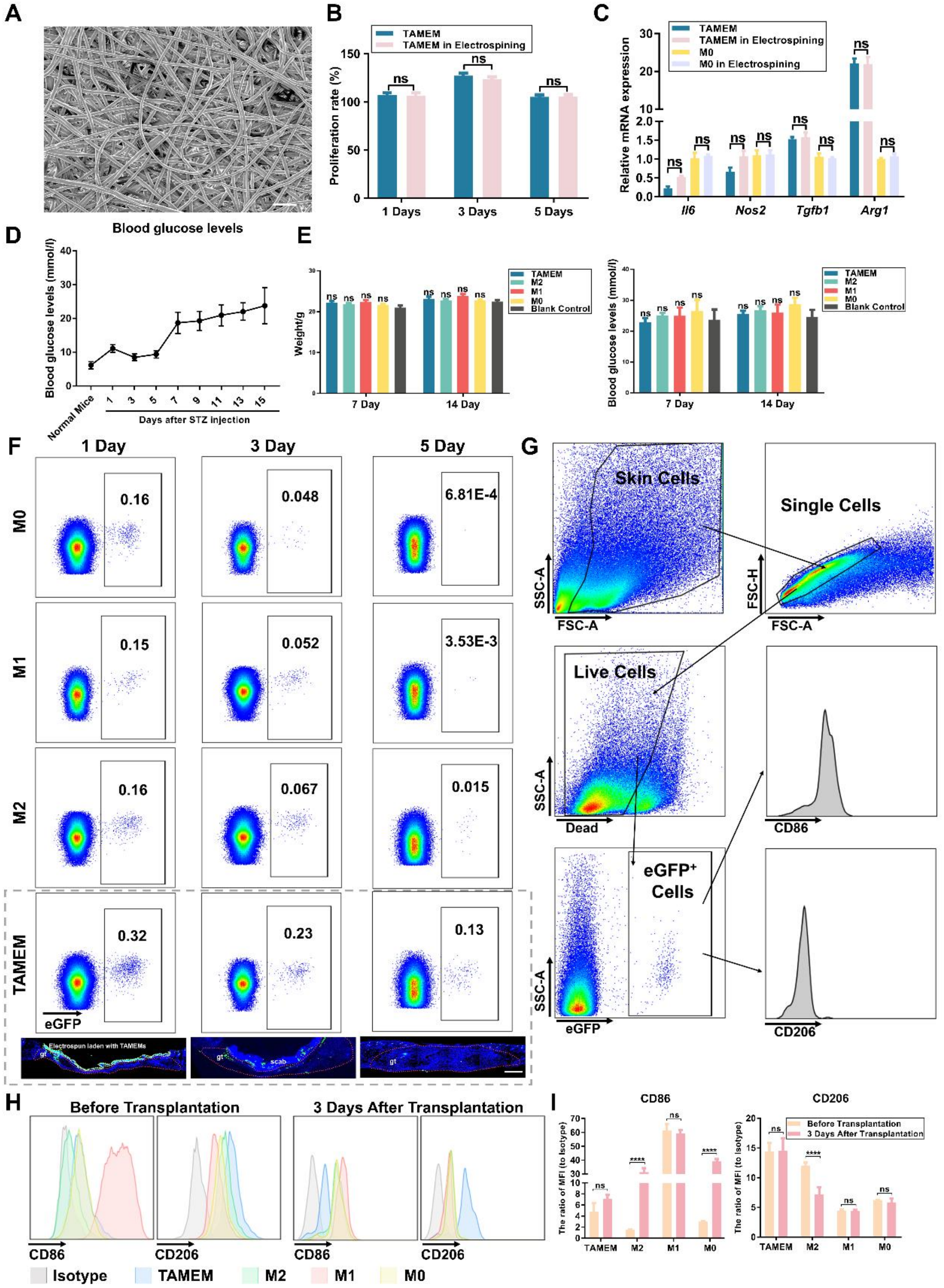

Appendix Figure S2 - Preparative tasks for TAMEMs transplantation.

A Representative SEM images of gelatin electrospinning (scale bar: 10 μm).

B Cell viability of TAMEMs was detected at each time points after seeding in gelatin electrospinning, the rates are presented as a percentage of the initial cell vitality at day 1 (ns: not significant ($P > 0.05$); n=3, biological replicates).

C Quantification of the expression of the macrophage polarization related genes (*Il6*, *Nos2*, *Tgfb1*, and *Arg1*) to compare TAMEMs/ M0 that seeded on the culture plate with them loaded in gelatin

electrospinning scaffolds, followed by culture in the same RPMI-1640 complete medium for 2 days (ns: not significant ($P > 0.05$); n=6, biological replicates).

D Measurement of the level of blood glucose through 15 days after STZ injection.

E Measurement of the level of blood glucose and weight at day 7 and day 14 after the transplantation of TAMEMs, M2, M1, and M0.

F Representative flow cytometry data of remaining eGFP$^+$ cells at each time point after the transplantation of eGFP$^+$ TAMEMs, eGFP$^+$ M2, eGFP$^+$ M1, and eGFP$^+$ M0, and representative images for locating the transplanted eGFP$^+$ TAMEMs, which were differentiated from eGFP$^+$ M0 isolated from C57BL/6 eGFP$^+$ mice, in the wound through 5 days (blue: DAPI; green: eGFP$^+$ TAMEMs; gt: granulation tissue; scale bar: 500 µm; n=3, biological replicates).

G Gating strategy for analyzing phenotypic changes (expression of CD86 and CD206) in transplanted cells by flow cytometry.

H Representative flow cytometry data of CD86 and CD206 expression in transplanted cells before and 3 days after transplantation.

I Quantification of the ratio of the MFI of CD86 and CD206 (compared to that of isotype) to compare the phenotypic changes of transplanted cells before and 3 days after transplantation (****$P < 0.0001$ and ns: not significant ($P > 0.05$); n=3, biological replicates).

Data information: Data represent means ± SD. The differences between groups were analyzed using two-way ANOVA with Tukey's multiple comparison test in (B, E, I) and a two-tailed unpaired t-test (C) in Graph Pad Prism 8.

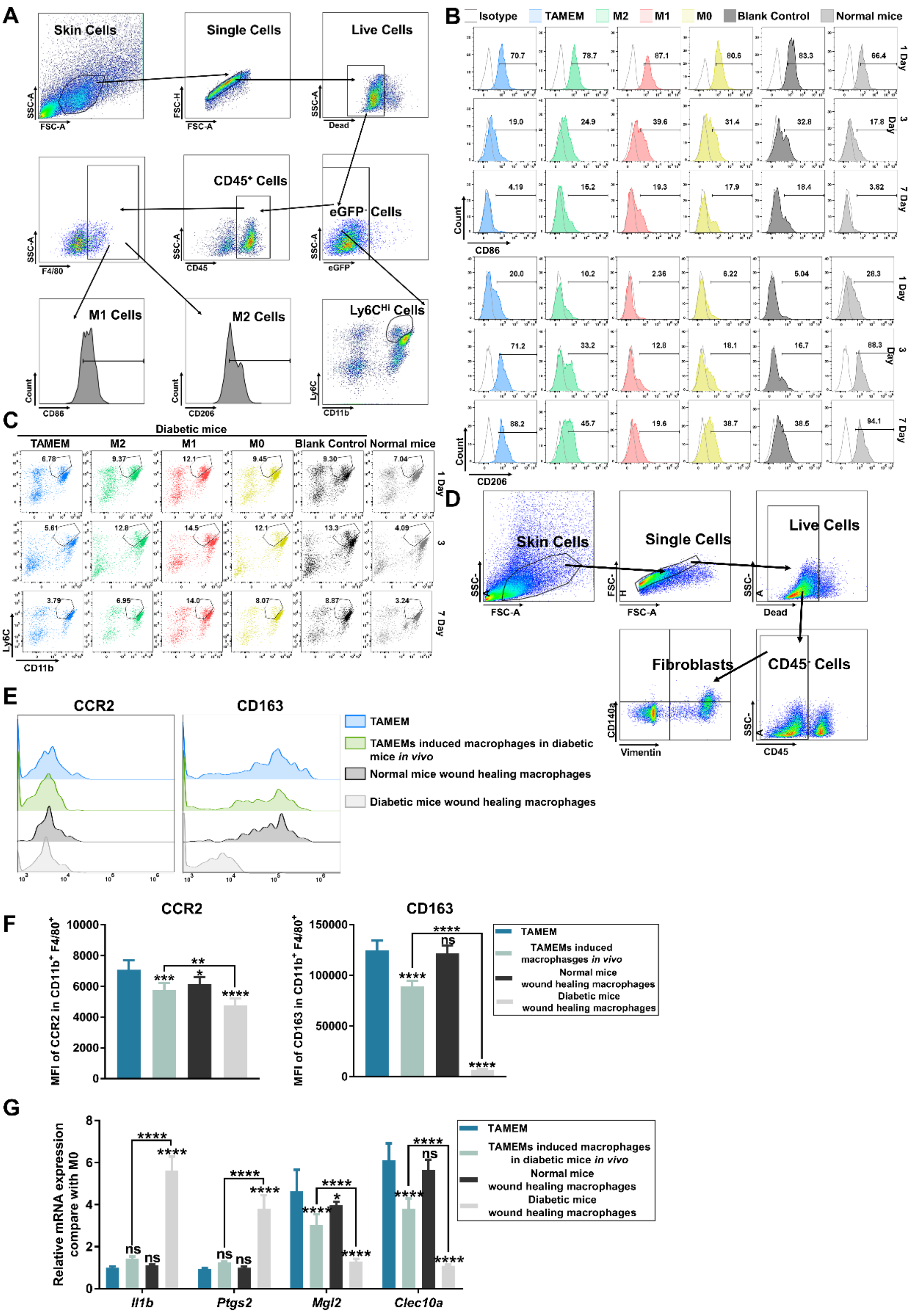

Appendix Figure S3 - Analysis of the phenotypes of host macrophages in diabetic mice.

A Gating strategy for analyzed the proportion of CD86 positive cells (M1), CD206 positive cells (M2), and Ly6C^{Hi} monocytes.

B, C Representative flow cytometry data of CD86 positive cells (M1) and CD206 positive cells (M2)

(B), and Ly6C^{Hi} monocytes (C) at day 1, 3, and 7 after the different treatments.

D Gating strategy for analyzed the proportion of fibroblasts.

E Representative flow cytometry data of CCR2 positive cells and CD163 positive cells at day 7 after the different treatments.

F Quantification of mean fluorescence intensity (MFI) for CCR2 and CD163 in TAMEMs and macrophages gating from wound tissues on postinjury days 7 (**P* < 0.05, ***P* < 0.01, ****P* < 0.001, and *****P* < 0.0001 vs TAMEMs; *n*=5, biological replicates).

G The real-time qPCR analysis of pro-inflammatory genes (*Il1b* and *Ptgs2*) and pro-inflammatory genes (*Mgl2* and *Clec10a*) in TAMEMs and macrophages gating from wound tissues on postinjury days 7 (**P* < 0.05, *****P* < 0.0001, and ns: not significant (*P* > 0.05) vs. TAMEMs; *n*=5, biological replicates).

Data information: Data represent means ± SD. The differences between groups were analyzed using ordinary one-way ANOVA and two-way ANOVA with Tukey's multiple comparison test in (F, G) in Graph Pad Prism 8.

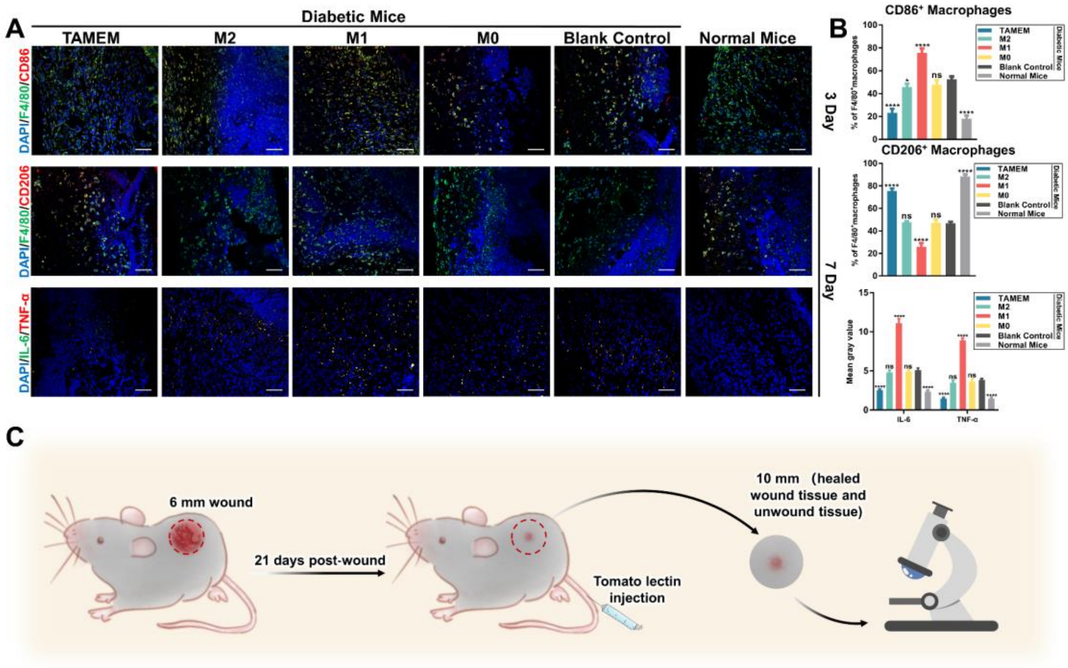

Appendix Figure S4 - Immunofluorescence staining of M1 and M2 and schematic diagram of the vascular perfusion experiment.

A Dual immunofluorescence staining for F4/80 (green) and CD86 (red) (day 3), F4/80 (green) and CD206 (red) (day 7), and IL-6 (green) and TNF-α (red) (day 7) was performed in wound tissues after the administration of different treatments (scale bar: 50 μm; *n*=3, biological replicates).

B Quantification of the numbers of CD86 positive cells and CD206 positive cells, and the mean gray value of pro-inflammatory cytokines (IL-6 and TNF-α) (**P* < 0.05, *****P* < 0.0001, and ns: not significant (*P* > 0.05) vs. the blank control group; *n*=3, biological replicates).

C Schematic diagram of the vascular perfusion experiment.

Data information: Data represent means ± SD. The differences between groups were analyzed using ordinary one-way ANOVA with Tukey's multiple comparison test in (B) in Graph Pad Prism 8.

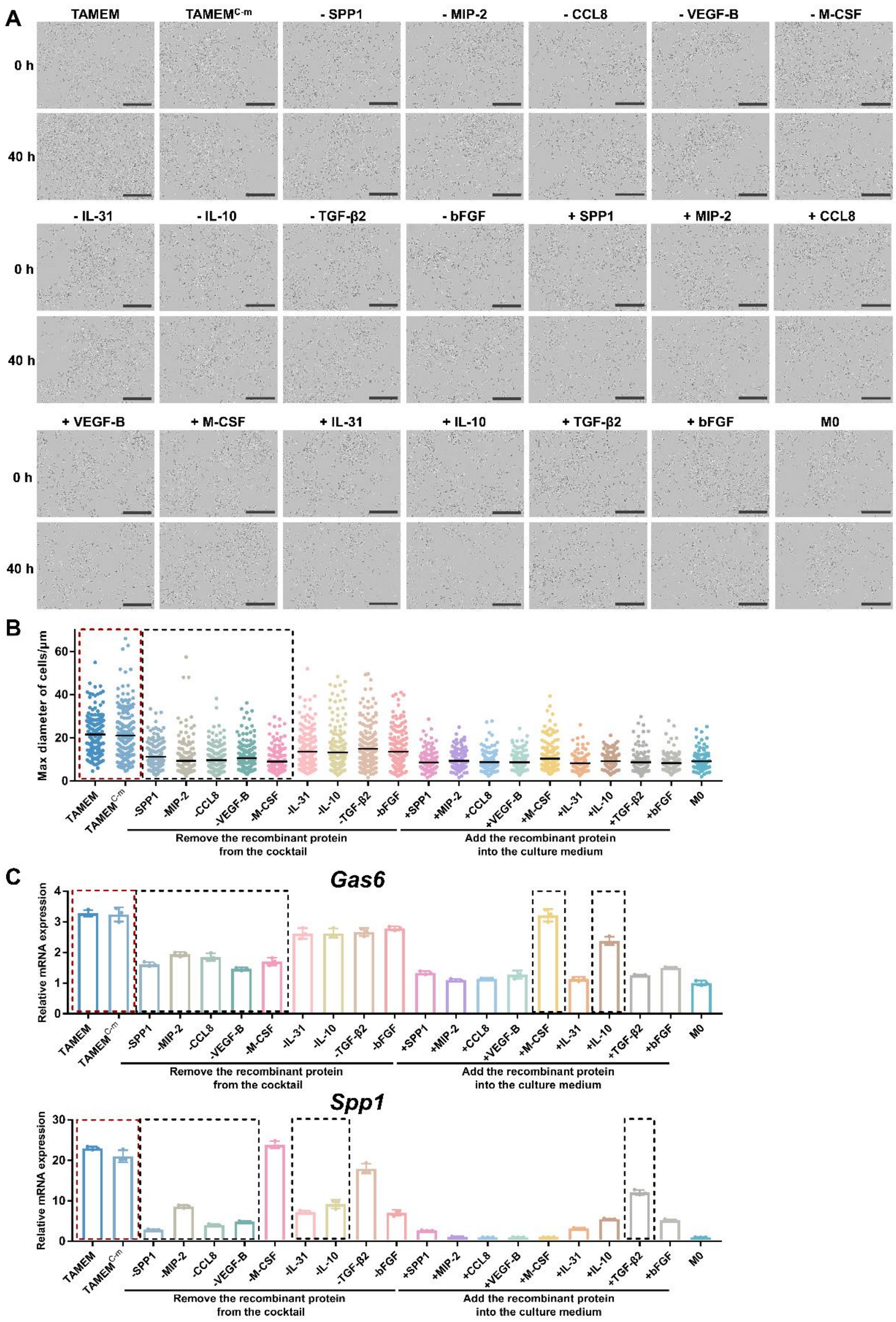

Appendix Figure S5 - Supplementary datas about TAMEMs^{C}.

A Representative pictures of two time points of the real-time image record using IncuCyte system to assess the proliferation and morphological changes in TAMEMs and others.

B Quantification of maximum diameter of TAMEMs and other cells.

C The real-time qPCR analysis of TAMEMs-specific genes (*Gas6* and *Spp1*) compare TAMEMs and

other cells.

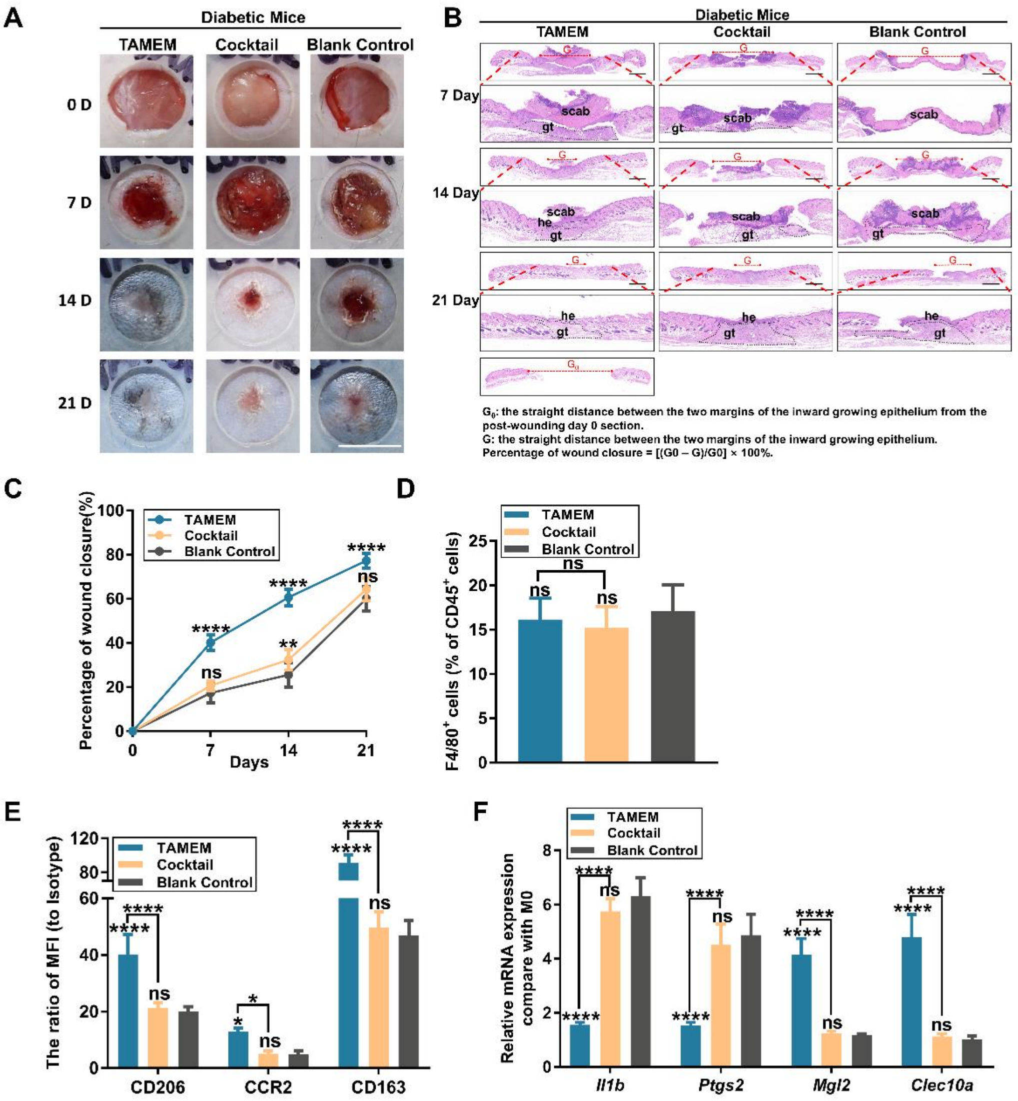

Appendix Figure S6 - Directly applying the cocktail can not replace delivering live TAMEMs to heal the diabetic wounds.

A Representative images of the wound closure process were captured during the 21-day in vivo experiments (scale bar: 500 µm; *n*=8, biological replicates).

B Representative images of full-thickness skin samples containing entire wound sites which are staining with H&E at each time point and calculation method of percentage of wound closure (gt: granulation tissue, he: hyperproliferative epithelium, scale bar: 1000 µm; *n*=8, biological replicates).

C The percentage of wound closure was determined at each time point (***P* < 0.01, *****P* < 0.0001 and ns: not significant (*P* > 0.05) vs. the blank control group; *n*=8, biological replicates).

D The percentage of F4/80$^+$ macrophages in CD45$^+$ lymphocyte at day 7 post-wounding (ns: not significant (*P* > 0.05) vs. the blank control group; *n*=5, biological replicates).

E Quantification of MFI for CD206, CCR2, and CD163 in macrophages gating from wound tissues with different treatments on postinjury days 7 (**P* < 0.05, *****P* < 0.0001 and ns: not significant (*P* > 0.05) vs. the blank control group; *n*=5, biological replicates).

F The real-time qPCR analysis of pro-inflammatory genes (*Il1b* and *Ptgs2*) and pro-inflammatory genes

(*Mgl2* and *Clec10a*) in n macrophages gating from wound tissues with different treatments on postinjury days 7 (****$P < 0.0001$ and ns: not significant ($P > 0.05$) vs. the blank control group; n=5, biological replicates).

Data information: Data represent means ± SD. The differences between groups were analyzed using ordinary one-way ANOVA with Tukey's multiple comparison test in (D) and two-way ANOVA with Tukey's multiple comparison test in (C, E, F) in Graph Pad Prism 8.

Appendix Table S1 - Primer sequences used for RT-qPCR.

Genes	Forward	Reverse
Gas6	CCGTTCAGACAGCACCTGGATTG	ACATGCCGTGGTTGATGGTTGG
Spp1	AAGAGCGGTGAGTCTAAGGAGTCC	TGGCTGCCCTTTCCGTTGTTG
Tfrc	CCCGTTGTTGAGGCAGACCTTG	CCTGATGACTGAGATGGCGGAAAC
Ccr2	AGCCTGATCCTGCCTCTACTTGTC	GCCCTGTGCCTCTTCTTCTCATTC
Arg1	CAGAAGAATGGAAGAGTCAG	CAGATATGCAGGGAGTCACC
Mrc1	CTCTGTTCAGCTATTGGACGC	CGGAATTTCTGGGATTCAGCTTC
Nos2	TGCATGGACCAGTATAAGGCAAGC	GCTTCTGGTCGATGTCATGAGCAA
Il6	CTTCCATCCAGTTGCCTTCTTG	AATTAAGCCTCCGACTTGTGAAG
Il8	CAAGGCTGGTCCATGCTCC	TGCTATCACTTCCTTTCTGTTGC
Acta2	GTCCCAGACATCAGGGAGTAA	TCGGATACTTCAGCGTCAGGA
Col1a1	CAACAGTCGCTTCACCTACAGC	GTGGAGGGAGTTTACACGAAGC
Tgfb1	CCTGTCCAAACTAAGGC	GGTTTTCTCATAGATGGCG
Vegfa	GTACCTCCACCATGCCAAGT	TCACATCTGCAAGTACGTTCG
Pdgfb	ATCGCCGAGTGCAAGACGCG	AAGCACCATTGGCCGTCCGA
Actb	GCTGGTCGTCGACAACGGCTC	CAAACATGATCTGGGTCATCTTTTC

Appendix Table S2 - The antibodies used in this study.

Antibodies	Source	Catalogue No.
FITC anti-mouse CD45	BioLegend	Cat # 103108
APC anti-mouse Ly-6G	BioLegend	Cat # 127614
PE/Cyanine7 anti-mouse Ly-6C	BioLegend	Cat # 128018
Brilliant Violet 711™ anti-mouse I-A/I-E	BioLegend	Cat # 107643
PE anti-mouse/human CD11b	BioLegend	Cat # 101208
APC-R700 Rat Anti-Mouse Siglec-F	BD Biosciences	Cat # 565183
Fixable Viability Stain 450	BD Biosciences	Cat # 562247
APC anti-mouse F4/80	BioLegend	Cat # 123116
PE anti-mouse CD206 (MMR)	BioLegend	Cat # 141706
PE anti-mouse CD86	BioLegend	Cat # 159204
PE anti-mouse CD80	BioLegend	Cat # 104707
PE anti-mouse CD163	BioLegend	Cat # 156703
PE anti-mouse CD192 (CCR2)	BioLegend	Cat # 150609
Mouse monoclonal anti-alpha smooth muscle Actin	Abcam	Cat # ab7817
Rabbit monoclonal anti-Collagen I	Abcam	Cat # ab34710
Rabbit monoclonal IκBα (44D4) Rabbit mAb	Cell Signaling	Cat # 4812S
Rabbit monoclonal Phospho-IκBα (Ser32) (14D4) Rabbit mAb	Cell Signaling	Cat # 2859S
Anti-rabbit IgG, HRP-linked Antibody	Cell Signaling	Cat # 7074P2
Anti-mouse IgG, HRP-linked Antibody	Cell Signaling	Cat # 7076P2
Rabbit monoclonal GAPDH (14C10) Rabbit mAb	Cell Signaling	Cat # 2118S
Rabbit monoclonal anti-Mannose Receptor	Abcam	Cat # ab64693
Rabbit monoclonal anti-CD86	Abcam	Cat # ab242142
Rat monoclonal anti-F4/80	Abcam	Cat # ab6640
Rabbit monoclonal TNF-α Rabbit mAb	Cell Signaling	Cat # 11948S
Rat monoclonal anti-IL-6	Abcam	Cat # ab191194
Rabbit polyclonal anti-CD31	Abcam	Cat # ab28364

Appendix Table S3 - The recombinant proteins used in this study.

Recombinant protein	Source	Catalogue No.
Recombinant Murine M-CSF	Peprotech	Cat # 315-02
Recombinant Murine IFN-γ	Peprotech	Cat # 315-05
Recombinant Murine IL-4	Peprotech	Cat # 214-14
Recombinant Murine IL-13	Peprotech	Cat # 210-13
Recombinant Murine Osteopontin	BioLegend	Cat # 763602
Recombinant Murine IL-31	Peprotech	Cat # 210-31
Recombinant Murine TGF-β2	R&D systems	Cat # 7346-B2-005/CF
Recombinant Murine MIP-2 (CXCL2)	Peprotech	Cat # 250-15
Recombinant Murine MCP-2 (CCL8)	Peprotech	Cat # 250-14
Recombinant Murine VEGF-B	R&D systems	Cat # 767-VE-010/CF
Recombinant Murine FGF-basic	Peprotech	Cat # 450-33
Recombinant Murine IL-10	Peprotech	Cat # 210-10
Recombinant Human M-CSF	Peprotech	Cat # 300-25
Recombinant Human IFN-γ	Peprotech	Cat # 300-02
Recombinant Human IL-4	Peprotech	Cat # 200-04
Recombinant Human IL-13	Peprotech	Cat # 200-13
Recombinant Human Osteopontin	Peprotech	Cat # 120-35
Recombinant Human IL-31	Peprotech	Cat # 200-31
Recombinant Human TGF-β2	Peprotech	Cat # 100-35B
Recombinant Human GRO-β (CXCL2)	Peprotech	Cat # 300-39
Recombinant Human MCP-2 (CCL8)	Peprotech	Cat # 300-15
Recombinant Human VEGF-B	Peprotech	Cat # 100-20B
Recombinant Human FGF-basic	Peprotech	Cat # 100-18B
Recombinant Human IL-10	Peprotech	Cat # 200-10
Recombinant Mouse VEGF-164	Biolegend	Cat # 583104
