# Supplementary material for: Tumor‐associated macrophages‐educated reparative macrophages promote diabetic wound healing
Source: EMBO Mol Med. 2022 Dec 21;15(2):e16671. doi: 10.15252/emmm.202216671 (PMC9906426; doi:10.15252/emmm.202216671)
Supplement: Supplementary file 2 — Expanded View Figures PDF [file EMMM-15-e16671-s008.pdf]

Expanded View Figures

Figure EV1. TAMEMs exhibit an anti-inflammatory and pro-healing phenotype.

A Heatmap of differentially expressed immune-related and growth factor transcripts between TAMEMs, M2, M1, and M0 cells, as determined using bulk RNA-seq analysis.
B Gene ontology (GO) enrichment of the upregulated genes in TAMEMs, M2, and M1 versus M0, respectively, as determined using bulk RNA-seq analysis.
C The real-time qPCR analysis of Mrc1, Arg1, Pdgfb, and Ang in differentiated TAMEMs (TAMEMs-Day 0) and TAMEMs cultured in RPMI-1640 complete medium for 4 Days (TAMEMs-Day 4) (ns: not significant ($P > 0.05$); $n = 6$, biological replicates).
D Heatmap of representative upregulated genes in comparison of TAMEMs, M2, and M1, as determined using Single-cell RNA-seq analysis.
E tSNE plot showing the clusters in TAMEMs.
F, G Slingshot pseudotime trajectory analyses of these five cell clusters in TAMEMs shown on a principal component plot (PC1 vs. PC2).
H Expression dynamics of representative marker genes in TAMEMs's five cell clusters via pseudotime trajectory and real-time polymerase chain reaction (PCR) analyses ($n = 3$, biological replicates).
I Predicted lineage schematic of TAMEMs based on the results of pseudotime trajectory and real-time analyses.
J MA plot for DGE analysis between TAMEMs and TAMs. Points that are significantly upregulated with an fdr < 0.05 are in pink and significantly downregulated with an fdr < 0.05 are in blue; all others are in gray.
K Gene expression patterns projected onto tSNE plots of Ly6c in TAMs (scale: log-transformed gene expression).

Data information: Data represent means $\pm$ SD. The differences between groups were analyzed using two-way ANOVA with Tukey's multiple comparison test in (E) in GraphPad Prism 8.

© 2022 The Authors

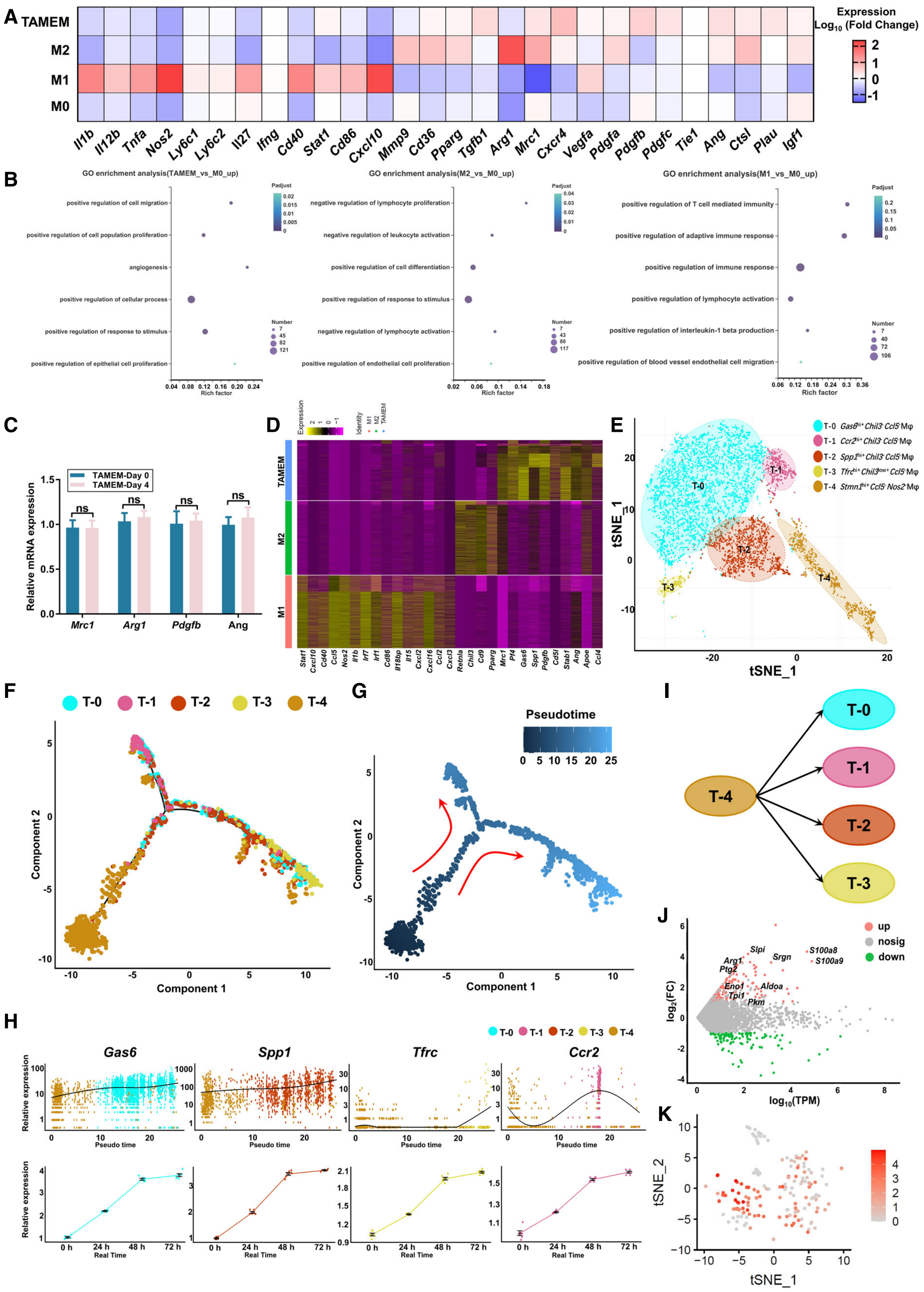

Figure EV1.

 © 2022 The Authors

Figure EV2. Validation of RNA sequencing results at the protein level.

A Analysis of the soluble cytokines expressed in the culture medium of TAMEMs, M2, M1, and M0 by mouse cytokine and angiogenesis array kit.

B Heatmap of gray value statistics of cytokine and angiogenesis array analysis results.

C The real-time qPCR analysis of M2 macrophage genes (Arg1 and Mrc1) in BMDMs treated with CMs from TAMEMs and TAMs for 48 h (***$P < 0.001$, ****$P < 0.0001$ vs. the normal medium group; $n = 6$, biological replicates).

D The proliferation rate of L929 cells pretreated with different CMs was analyzed using the CCK-8 kit (**$P < 0.01$ and ns: not significant; $n = 6$) and ELISA for type I Collagen in L929 cells after 48-h treatment with different CMs (*$P < 0.05$, ***$P < 0.001$ and ns: not significant ($P > 0.05$) vs. the normal medium group; $n = 6$, biological replicates).

Data information: Data represent means ± SD. The differences between groups were analyzed using ordinary one-way ANOVA in and two-way ANOVA with Tukey's multiple comparison test in (C, D) in GraphPad Prism 8.

© 2022 The Authors

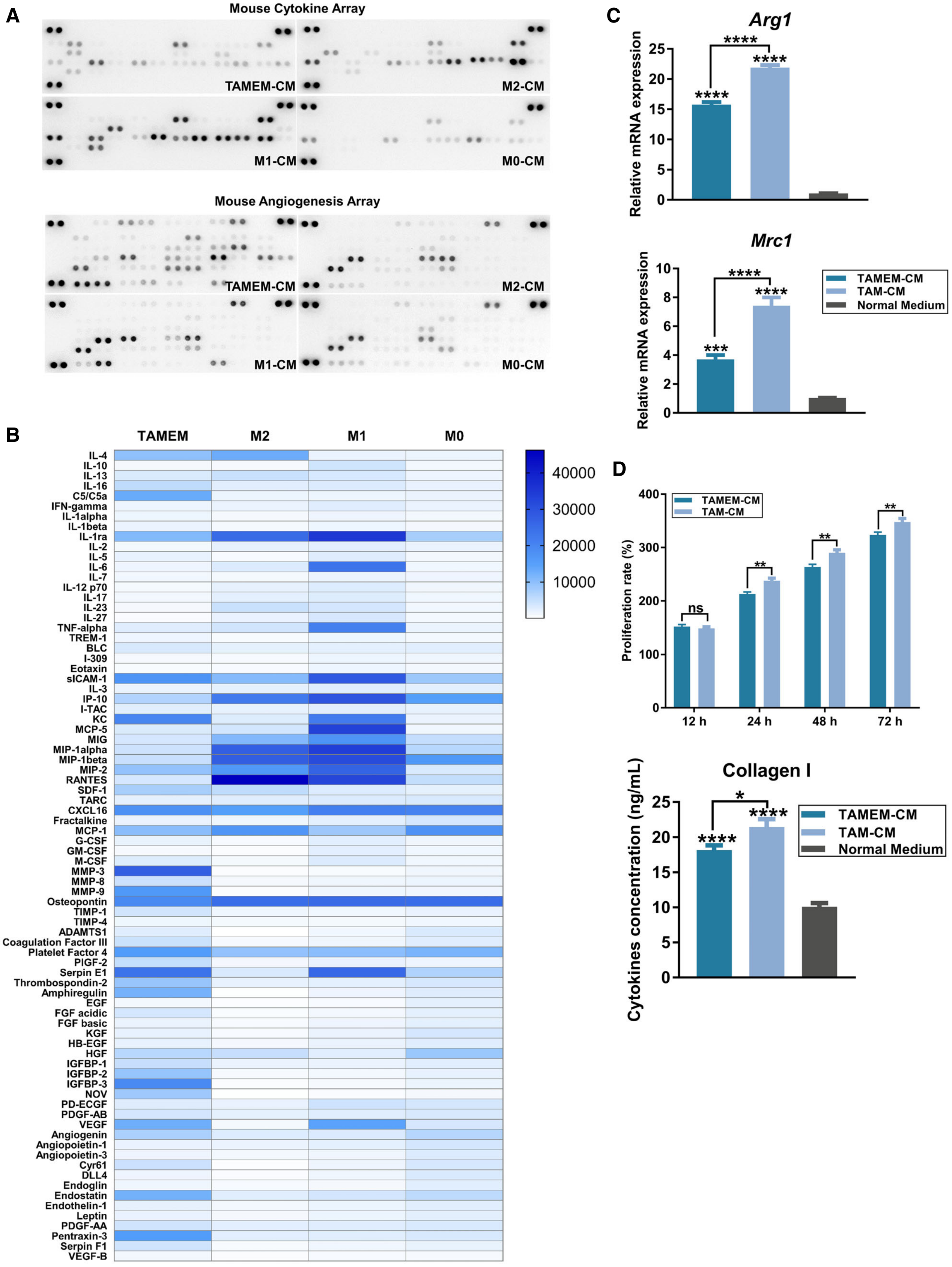

Figure EV2.

 © 2022 The Authors

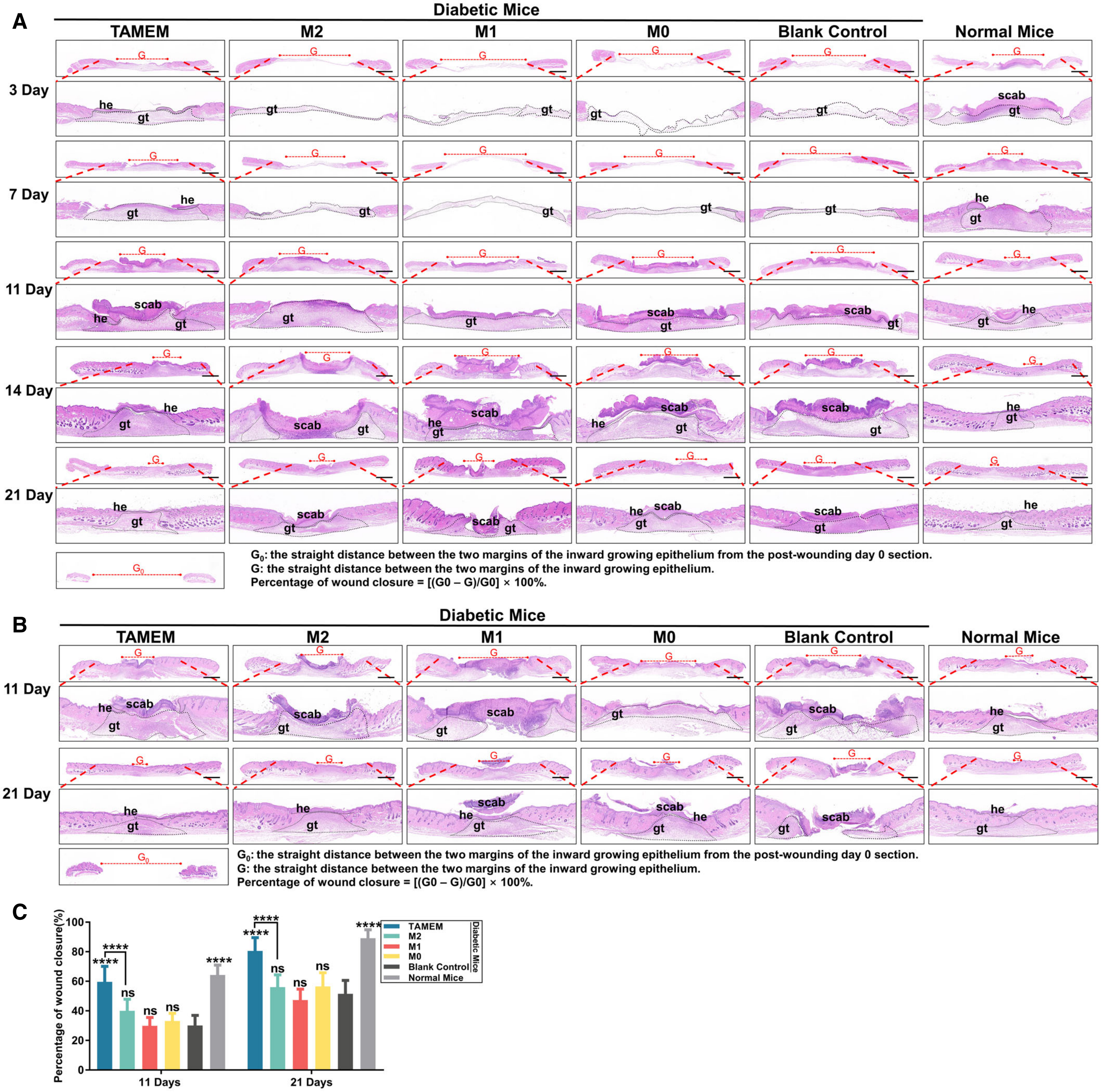

Figure EV3. TAMEMs accelerate the healing of skin wounds in both STZ-induced T1D mice and db/db T2D mice.

A Representative images of full-thickness skin samples containing entire wound sites, which are staining with H&E at each time point and calculation method of percentage of wound closure in STZ-induced T1D mice (gt: granulation tissue, he: hyperproliferative epithelium, scale bar: 1,000 μm; $n = 8$, biological replicates).

B H&E staining for full-thickness skin samples containing the entire wound area on day 11 and 21 in db/db T2D mice (gt: granulation tissue, he: hyperproliferative epithelium, scale bar: 1,000 μm; $n = 8$, biological replicates).

C The percentage of wound closure determined at each time point (****$P < 0.0001$ and ns: not significant ($P > 0.05$) vs. the blank control group; $n = 8$, biological replicates).

Data information: Data represent means ± SD. The differences between groups were analyzed using two-way ANOVA with Tukey's multiple comparison test in (C) in GraphPad Prism 8.

© 2022 The Authors

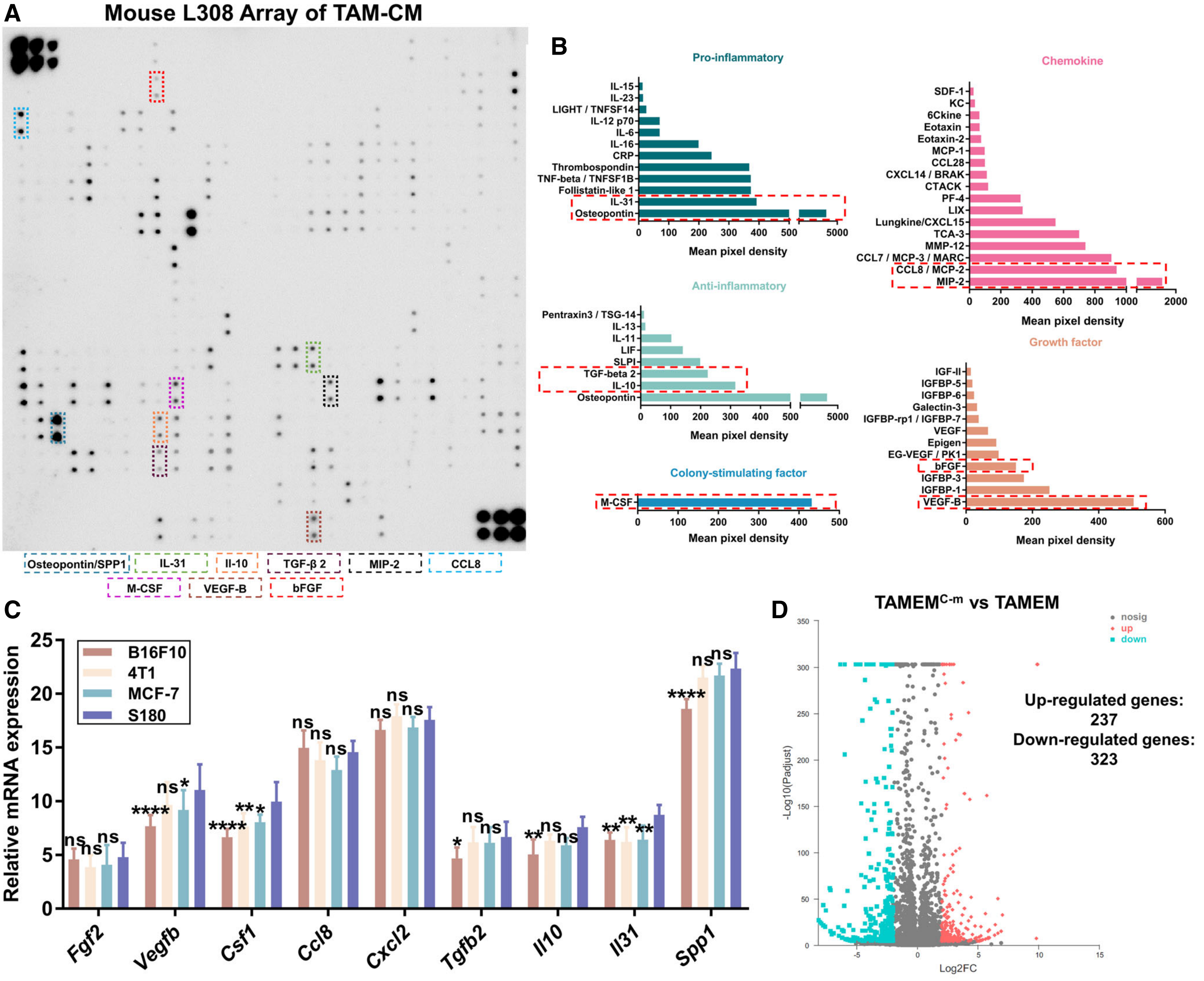

Figure EV4. Identification of a recombinant protein cocktail to replace TAMs in generating TAMEMs.

A Antibody array analysis of cell-free TAMs-CM using a Biotin Label-based L-Series Mouse Antibody Array 308 Membrane Kit that could detect 308 different mouse target proteins, the cytokines that we selected were highlighted in dotted boxes.

B Quantification and categorization of the detected proteins (excluding unlikely ingredients), based on the mean gray value and functions, the cytokines that we selected were highlighted in red dotted boxes.

C The real-time qPCR analysis of genes of the selected nine proteins in TAMs from different tumor models (*$P < 0.05$, **$P < 0.01$, ****$P < 0.0001$ and ns: not significant ($P > 0.05$) vs. S180; $n = 3$, biological replicates).

D Volcano plot for DGE analysis between TAMEMs and TAMEMs$^{C\text{-}m}$.

Data information: Data represent means ± SD. The differences between groups were analyzed using two-way ANOVA with Tukey's multiple comparison test in (C) in GraphPad Prism 8.

 © 2022 The Authors

Figure EV5. Transplantation of cytokine-trained human monocytes (termed TAMEMs^{C-h}) accelerates wound healing in immunocompromised diabetic mice.

A Representative images of full-thickness skin samples containing entire wound sites, which are staining with H&E at each time point and calculation method of percentage of wound closure (gt: granulation tissue, he: hyperproliferative epithelium, scale bar: 1,000 μm; $n = 8$, biological replicates).

B Dual immunofluorescence staining for F4/80 (green) and CD86 (red; day 3), F4/80 (green) and CD206 (red; day 7), and IL-6 (green) and TNF-α (red; day 7) was performed in wound tissues after the administration of different treatments (scale bar: 100 μm; $n = 3$, biological replicates).

C Quantification of the numbers of pro-inflammatory macrophages (CD86-positive cells) and anti-inflammatory macrophages (CD206-positive cells), and the mean gray value of pro-inflammatory cytokines (IL-6 and TNF-α) (**$P < 0.01$, ***$P < 0.001$, ****$P < 0.0001$, and ns: not significant ($P > 0.05$) vs. the blank control group; $n = 3$, biological replicates).

D Representative immunostaining images of CD31 (red; endothelial marker, for new capillary formation) at day 7 and α-SMA (green; smooth muscle marker, for vascular maturation) at day 14, which are higher magnification images of the yellow boxed region in the entire wound sites (gt: granulation tissue, he: hyperproliferative epithelium, scale bar: 100 μm; $n = 8$, biological replicates).

E Quantification of the $CD31^+$ vessels at day 7 and $CD31^+$ $\alpha\text{-SMA}^+$ vessels and $CD31^+$ $\alpha\text{-SMA}^-$ vessels at day 14 (****$P < 0.0001$ and ns: not significant ($P > 0.05$) vs. the blank control group; $n = 8$, biological replicates).

F Orientational distribution based on pixel orientation ($n = 3$, biological replicates).

Data information: Data represent means ± SD. The differences between groups were analyzed using ordinary one-way ANOVA with Tukey's multiple comparison test in (C, E) in GraphPad Prism 8.

© 2022 The Authors

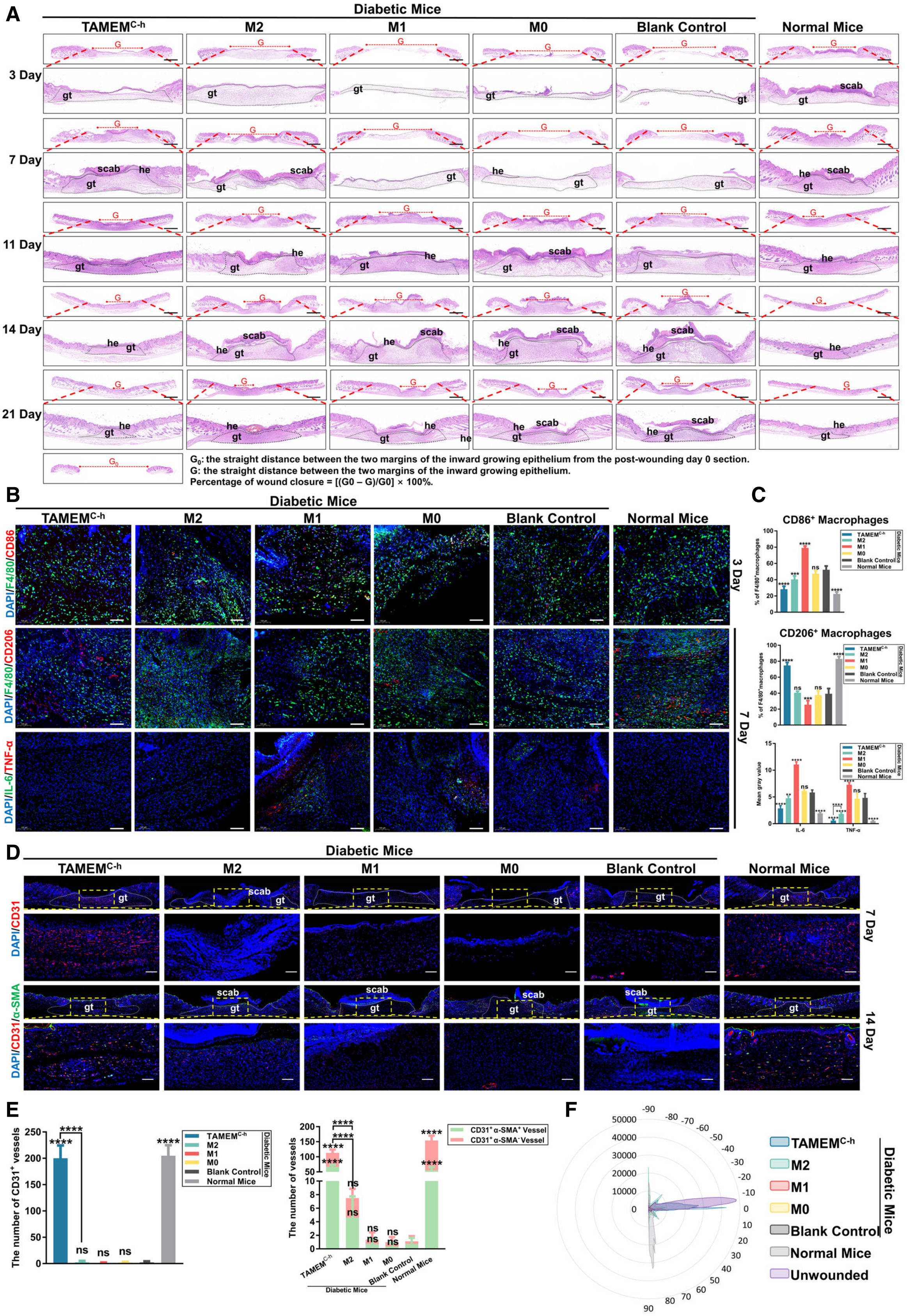

Figure EV5.

 © 2022 The Authors
