# Supplementary material for: Tumor‐associated macrophages‐educated reparative macrophages promote diabetic wound healing
Source: EMBO Mol Med. 2022 Dec 21;15(2):e16671. doi: 10.15252/emmm.202216671 (PMC9906426; doi:10.15252/emmm.202216671)
Supplement: Supplementary file 6 — Table EV1 [file EMMM-15-e16671-s007.docx]

# Table EV1. The proteins list detected in the TAMs-CM.

| **Serum Amyloid A1** | **Soggy-1** | **CCL28** | **IGFBP-5** |
| --- | --- | --- | --- |
| **ICK** | **Frizzled-6** | **EG-VEGF / PK1** | **DKK-1** |
| **Osteopontin** | **P-Selectin** | **CCL1 / I-309 / TCA-3** | **IL-6 R** |
| **LIX** | **Prolactin** | **MCP-1** | **GDF-1** |
| **MMP-2** | **TCCR / WSX-1** | **Epigen** | **Fas Ligand** |
| **CCL8 / MCP-2** | **CCR9** | **AgRP** | **IL-13** |
| **CCL7 / MCP-3 / MARC** | **OX40 Ligand / TNFSF4** | **CCR4** | **IFN-alpha / beta R1** |
| **IL-1 RI** | **IGFBP-1** | **DR3 / TNFRSF25** | **IGF-II** |
| **IL-17R** | **TIMP-2** | **Eotaxin-2** | **IL-23** |
| **MMP-12** | **CRP** | **Progranulin** | **Kremen-2** |
| **TCA-3** | **IFN-gamma R1** | **IL-6** | **TLR3** |
| **ICAM-2 / CD102** | **GFR alpha-3 / GDNF R alpha-3** | **IL-12 p70** | **TL1A / TNFSF15** |
| **Lymphotoxin beta R / TNFRSF3** | **SPARC** | **VEGF** | **IL-13 R alpha 2** |
| **ICAM-1** | **TGF-beta 2** | **Eotaxin** | **GDF-9** |
| **CXCR6** | **WISP-1 / CCN4** | **IL-3 R alpha** | **IL-15** |
| **Osteoactivin / GPNMB** | **IGFBP-2** | **TFPI** | **MFRP** |
| **Lungkine/CXCL15** | **IL-16** | **6Ckine** | **Resistin** |
| **IL-23 R** | **SLPI** | **DAN** | **TLR2** |
| **VEGF-B** | **GDF-8** | **TROY** | **Fit-3 Ligand** |
| **IL-15 R alpha** | **TGF-beta 3** | **Glut2** | **IL-1 alpha** |
| **IL-28 / IFN-lambda** | **CCR6** | **TMEFF1 / Tomoregulin-1** | **MIG** |
| **M-CSF** | **IGFBP-3** | **Dtk** | **TSLP R** |
| **Urokinase** | **CD11b** | **IL-22BP** | **CXCL16** |
| **FGF R5 beta** | **MMP-3** | **IGFBP-rp1 / IGFBP-7** | **IFN-beta** |
| **IL-31** | **Endostatin** | **KC** | **IL-1 R4 / ST2** |
| **Follistatin-like 1** | **b FGF** | **Galectin-3** | **TECK** |
| **TNF-beta / TNFSF1B** | **LIF** | **SDF-1** | **FCrRIIB / CD32b** |
| **Thrombospondin** | **Neuregulin-3 / NRG3** | **LIGHT / TNFSF14** | **ICAM-5** |
| **TGF-beta RII** | **Endocan** | **IGFBP-6** | **IL-10** |
| **TIMP-1** | **FGF R4** | **MMP-24 / MT5-MMP** | **Axl** |
| **IL-27** | **CTACK** | **GDF-3** | **CCR3** |
| **LRP-6** | **Common gamma Chain / IL-2 R gamma** | **Ubiqultin** | **Fractalkine** |
| **TWEAK R / TNFRSF12** | **Fas / TNFRSF6** | **MIP-1 gamma** | **GFR alpha-2 / GDNF R alpha-2** |
| **MIP-2** | **CXCL14 / BRAK** | **IL-31 RA** | **Artemin** |
| **PF-4** | **VE-Cadherin** | **MMP-9** | **CXCR4** |
| **Pentraxin3 / TSG-14** | **IL-11** | **GDF-5** | **LEPTIN(OB)** |
