# Supplementary material for: Tumor‐associated macrophages‐educated reparative macrophages promote diabetic wound healing
Source: EMBO Mol Med. 2022 Dec 21;15(2):e16671. doi: 10.15252/emmm.202216671 (PMC9906426; doi:10.15252/emmm.202216671)
Supplement: Supplementary file 7 — PDF+ [file EMMM-15-e16671-s005.pdf]

Article

TRANSPARENT PROCESS OPEN ACCESS

EMBO Molecular Medicine

Tumor-associated macrophages-educated reparative macrophages promote diabetic wound healing

Ruoyu Mu[1], Zhe Zhang[1,2], Congwei Han[1,3], Yiming Niu[1,3], Zhen Xing[3], Zhencheng Liao[1], Jinzhi Xu[3], Ningyi Shao[4], Guokai Chen[4], Junfeng Zhang[3], Lei Dong[3,*] & Chunming Wang[1,2,5,**]

Abstract

Nonhealing diabetic wounds, with persistent inflammation and damaged vasculature, have failed conventional treatments and require comprehensive interference. Here, inspired by tumor-associated macrophages (TAMs) that produce abundant immunosuppressive and proliferative factors in tumor development, we generate macrophages to recapitulate TAMs' reparative functions, by culturing normal macrophages with TAMs' conditional medium (TAMs-CM). These TAMs-educated macrophages (TAMEMs) outperform major macrophage phenotypes (M0, M1, or M2) in suppressing inflammation, stimulating angiogenesis, and activating fibroblasts *in vitro*. When delivered to skin wounds in diabetic mice, TAMEMs efficiently promote healing. Based on TAMs-CM's composition, we further reconstitute a nine-factor cocktail to train human primary monocytes into TAMEMs$^{C\text{-}h}$, which fully resemble TAMEMs' functions without using tumor components, thereby having increased safety and enabling the preparation of autologous cells. Our study demonstrates that recapitulating TAMs' unique reparative activities in nontumor cells can lead to an effective cell therapeutic approach with high translational potential for regenerative medicine.

Keywords adoptive cell transfer; diabetes; macrophages; tumor-associated macrophages (TAMs); wound healing
Subject Categories Skin; Stem Cells & Regenerative Medicine
DOI 10.15252/emmm.202216671 | Received 29 July 2022 | Revised 29 November 2022 | Accepted 2 December 2022
EMBO Mol Med (2022) e16671

Introduction

Macrophages play a pivotal role in orchestrating the repair of multiple tissue and organs, and their dysfunction abrogates wound healing. A typical case is diabetic wounds that affect 100 million people worldwide (Perez *et al*, 2019), where pro-inflammatory macrophages that initiate healing fail to transit into anti-inflammatory states as they do in normal wounds and further recruit more inflammatory monocytes to prolong inflammation and halt healing (Boniakowski *et al*, 2017; Kimball *et al*, 2018). Delivering reparative macrophages to rectify the local immune niche is a potential strategy, as tested in both murine and human models (Danon *et al*, 1989, 1997; Zuloff-Shani *et al*, 2010; Hu *et al*, 2017; Jayme *et al*, 2020). But what phenotype is optimal for the healing remains an open question (Falanga, 2005; Jetten *et al*, 2014; Yuan *et al*, 2016). Throughout skin repair, macrophages change their phenotypes at the right timing to exert sequential activities, exhibiting a delicate balance of pro- and anti-inflammatory characteristics to drive angiogenesis (Eming *et al*, 2014), promote cell proliferation, and modulate inflammation (Novak & Koh, 2013). Such a dynamic state can hardly be defined by simplified pro- (M1)/anti-inflammatory (M2) phenotypes or up/downregulation of a few genes (Jetten *et al*, 2014; Yuan *et al*, 2016). Identifying the approach to generate macrophages with comprehensive healing activities will significantly help to develop new cell therapies for tissue repair.

Tumor-associated macrophages (TAMs) provide unexpected inspiration to this goal. In tumor development, TAMs play essential and diverse roles in shaping the immunosuppressive, proliferative microenvironment, through paracrine secretion of abundant growth factors and anti-inflammatory cytokines (Hua & Bergers, 2019). These cells promote angiogenesis (Lin & Pollard, 2007), vascularization (Lin *et al*, 2006), and extracellular matrix (ECM) remodeling (Poltavets *et al*, 2018); they also inhibit antitumor responses of T cells and switch other immunocytes into anti-inflammatory phenotypes (Qian & Pollard, 2010; Motz & Coukos, 2011; Dvorak, 2015; Fujimura *et al*, 2018). Such multifold activities adequately mirror the comprehensive functions demanded by nonhealing diabetic wounds. However, although TAMs may serve as a benchmark in functions, they cannot directly be applied as therapeutic cells—

1 Institute of Chinese Medical Sciences & State Key Laboratory of Quality Research in Chinese Medicine, University of Macau, Macau SAR, China
2 Zhuhai UM Science & Technology Research Institute, University of Macau, Hengqin, China
3 School of Life Sciences & State Key Laboratory of Pharmaceutical Biotechnology, Nanjing University, Nanjing, China
4 Department of Biomedical Sciences, Faculty of Health Sciences, University of Macau, Macau SAR, China
5 Department of Pharmaceutical Sciences, Faculty of Health Sciences, University of Macau, Macau SAR, China
*Corresponding author. Tel: +86 25 8981320; E-mail: leidong@nju.edu.cn
**Corresponding author. Tel: +853 88224696; E-mail: cmwang@umac.mo

© 2022 The Authors. Published under the terms of the CC BY 4.0 license.

because delivering tumor-derived cells to patients has obvious safety issues; and, cell therapies prefer autologous transplantation, but diabetic patients without tumors have no TAMs available.

We hypothesized that TAMs could, fully or partially, pass on their reparative features to nonpolarized macrophages through cytokine secretion. As such, nontumor and even autologous macrophages can recapitulate TAMs' functions to promote healing. Our hypothesis was based on the mechanisms by which TAMs consolidate the tumor microenvironment. First, TAMs secrete abundant interleukin (IL)-10 (Sica *et al*, 2000), adrenomedullin (Chen *et al*, 2011), and vascular endothelial growth factors (VEGF; Lai *et al*, 2019) to preserve macrophages' immunosuppressive phenotype through various mechanisms (Sica *et al*, 2000; Chen *et al*, 2011; Lai *et al*, 2019). Similarly, nontumor-derived anti-inflammatory macrophages maintain their phenotype through autocrine secretion of insulin growth factor (IGF)-1 (Tonkin *et al*, 2015). Second, TAMs switch other macrophages (e.g., infiltrated monocytes) into immunosuppressive and pro-angiogenic phenotypes through CCR1/CCL3 axis, as well as TAMs-derived IL-10 and transforming growth factor (TGF)-β (Cassetta & Pollard, 2020). Such "amplification loop" mechanisms (Solinas *et al*, 2009) motivated us to culture nonpolarized macrophages with TAMs' secretion to prepare TAMs-educated macrophages (TAMEMs) that exert TAMs' reparative functions for wound healing.

To verify our hypothesis, we explored the condition to prepare TAMEMs from murine bone marrow-derived macrophages, assessed TAMEMs' phenotype through single-cell RNA sequencing, and examined their regenerative capacities in both type I and II diabetic mouse models. Further, we identified a cocktail of human recombinant proteins mimicking the TAMs' secretion to train human peripheral blood monocytes into cytokine-induced human TAMEMs (TAMEMs$^{C\text{-}h}$), which involved no tumor components throughout the preparation and hence enhanced translational potential.

Results

Preparation of TAMEMs from primary murine macrophages

To prepare macrophages with comprehensive healing functions, we first verified the possibility of recapitulating the reparative features of TAMs in "normal" macrophages—both primarily harvested from mice. First, we established S180 heterotopic tumor model in female C57BL/6J mice and harvested the tumor with 1 cm diameter, which contained the highest proportion of F4/80$^+$CD11b$^+$ cells—hence providing the highest yield of TAMs (Priceman *et al*, 2010). We chose this specific tumor type based on our previous study that its homogenate most potently induced IL-10 secretion in murine BMDMs and type I collagen production in mouse embryonic fibroblasts (MEFs), compared with homogenates from other tumor models (Wang *et al*, 2020). Then, through fluorescence-activated cell sorting (FACS), we sorted TAMs encompassing five subsets gated upon Ly6C and MHC II (Movahedi *et al*, 2010; Appendix Fig S1A). Next, we cultured the harvested TAMs for 15 days (cells morphology; Appendix Fig S1B), collected their supernatant, and used the supernatant to culture primary bone marrow-derived macrophages (BMDMs) for 2 days to obtain TAMs-educated macrophages (TAMEMs). In parallel, we induced BMDMs into classical M1 (100 ng/ml LPS and 40 ng/ml IFN-γ, 2 days) or M2 (40 ng/ml IL-4 and 20 ng/ml IL-13, 2 days; also defined as M2a) phenotype for comparison (Yao *et al*, 2019), as illustrated in Fig 1A.

Tumor-associated macrophages-educated macrophages exhibited characteristics in morphology, growth, and surface marker expression. First, at day 0 (when we obtained TAMEMs and other macrophage subtypes), TAMEMs were as elongated as M2 cells but had more antennae, unlike unpolarized macrophages (M0) in mixed spheroidal and spindle shapes or M1 cells in a round shape. At day 4, TAMEMs kept an elongated morphology while others, including M2, became smaller and spheroidal (Wright-Giemsa staining, Fig 1B). Second, TAMEMs were the only group of the tested macrophages that could proliferate through day 3 and maintained over 50% viability by day 11, when others kept losing viability to below 50% (Fig 1C). Immunofluorescent (IF) staining for Ki67 (Fig 1D) and Incucyte® live-cell monitoring for 48 h (Movie EV1 and Appendix Fig S1C) confirmed that only TAMEMs actively proliferated. Third, TAMEMs expressed a combination of M1 and M2 surface markers. Their M1 markers (CD86, CD80, and MHC II) were in moderate levels, lower than in M1 cells but higher than in M2 cells; their CCR2 and typical M2 markers (CD206 and CD163) were higher than in all other groups, according to flow cytometry (Fig 1E) and IF staining (for CD86 and CD206; Fig 1F). The data suggested that TAMEMs exhibited both similarities and differences between M1 and M2 macrophages, which motivated in-depth bioinformatics analysis as described below.

TAMEMs exhibit an anti-inflammatory and pro-healing phenotype

To assess the phenotype of TAMEMs in detail, we first used bulk RNA sequencing (RNA-seq) to analyze the differential genes between TAMEMs and the other three cells (Fig 2A). Compared with

Figure 1. Preparation of TAMEMs from primary murine macrophages.

- A Schematic diagram of the generation of TAMEMs.
- B Comparison of Wright-Giemsa staining (morphology) of freshly induced (Day 0) TAMEMs and typical macrophage subtypes and their continued cultured for 4 days in RPMI-1640 complete medium (Day 4; scale bar: 10 μm; $n = 3$, biological replicates).
- C The viability curve of TAMEMs and typical macrophage subtypes, with the survival rate presented as a percentage of the initial cell vitality at day 1 (**$P < 0.01$ and ****$P < 0.0001$ vs. the M0 group; $n = 6$, biological replicates).
- D Immunofluorescent staining for Ki67 in TAMEMs and typical macrophage subtypes (scale bar: 10 μm; $n = 3$, biological replicates).
- E Quantification of mean fluorescence intensity (MFI) for CD80, CD86, CD206, CD163, CCR2, and MHC II in TAMEMs and typical macrophage subtypes via flow cytometry (*$P < 0.05$, ***$P < 0.001$, and ****$P < 0.0001$; $n = 6$, biological replicates).
- F Representative immunofluorescent (IF) staining images for CD86 and CD206 in TAMEMs and typical macrophage subtypes (scale bar: 10 μm; $n = 6$, biological replicates).

Data information: Data represent mean ± SD. The differences between groups were analyzed using two-way ANOVA with Tukey's multiple comparison test in (C) and ordinary one-way ANOVA with Tukey's multiple comparison test in (E) in GraphPad Prism 8.

© 2022 The Authors

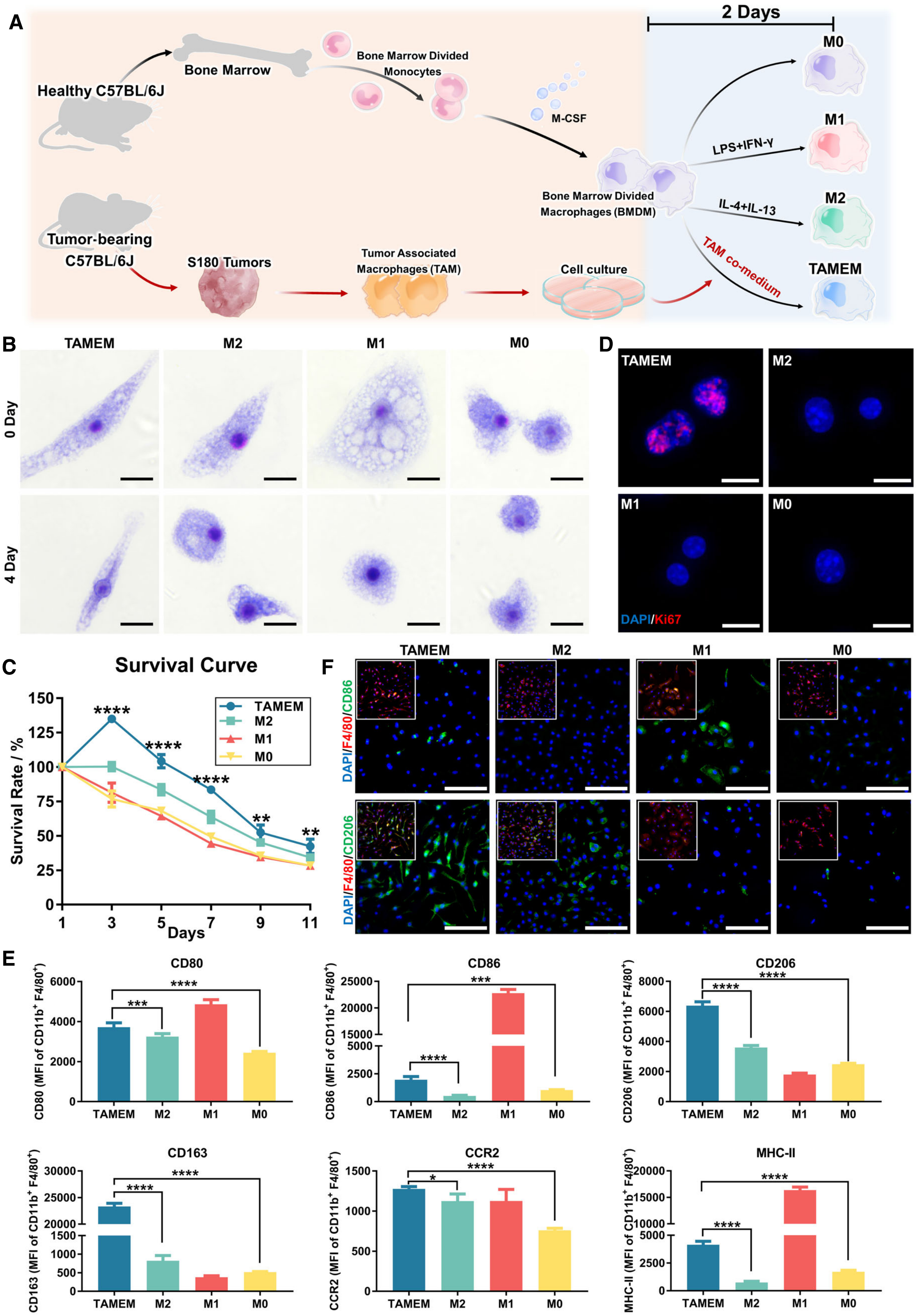
A
2 Days
Healthy C57BL/6J
Bone Marrow
Bone Marrow Divided Monocytes
M-CSF
Bone Marrow Divided Macrophages (BMDM)
M0
M1
LPS+IFN-γ
M2
IL-4+IL-13
TAM co-medium
TAMEM
Tumor-bearing C57BL/6J
S180 Tumors
Tumor Associated Macrophages (TAM)
Cell culture
B
TAMEM
M2
M1
M0
0 Day
4 Day
D
TAMEM
M2
M1
M0
DAPI/Ki67
C
Survival Curve
Survival Rate / %
Days
TAMEM
M2
M1
M0
F
TAMEM
M2
M1
M0
DAPI/F4/80/CD86
DAPI/F4/80/CD206
E
CD80
CD80 (MFI of CD11b+ F4/80+)
CD86
CD86 (MFI of CD11b+ F4/80+)
CD206
CD206 (MFI of CD11b+ F4/80+)
CD163
CD163 (MFI of CD11b+ F4/80+)
CCR2
CCR2 (MFI of CD11b+ F4/80+)
MHC-II
MHC-II (MFI of CD11b+ F4/80+)

Figure 1.

M0, TAMEMs highly expressed chemokines (e.g., *Ccl8* and *Ccl12*), anti-inflammatory markers (e.g., *Arg1* and *Mrc1*), and growth factors (e.g., *Ang* and *Pdgfc*). Compared with M1, TAMEMs expressed more growth factors (e.g., *Pdgfc*, *Fgf1*, and *Hgf*) and anti-inflammatory markers (e.g., *Mrc1* and *Il10*). Compared with M2, TAMEMs expressed more *Cx3cr1* (an important marker for resolving macrophages in diabetic wounds; Boniakowski *et al*, 2017; Burgess *et al*, 2019) and certain growth factors (e.g., *Pdgfc* and *Vegfb*). In addition, TAMEMs expressed a wider variety of growth factors and more types of pro-/anti-inflammatory markers than did M1/M2 cells, as shown in Fig EV1A. The GO enrichment analysis for each group against M0 revealed that TAMEMs exhibited pro-regenerative functions, including promoting cell proliferation, cell migration, and angiogenesis (Fig EV1B). Especially compared with M2 macrophages that are commonly considered to have regenerative phenotypes, TAMEMs presented a similar profile in expressing multiple genes related to wound healing, while producing a greater variety of pro-regenerative growth factors (Fig 2B). TAMEMs maintained their anti-inflammatory and pro-healing phenotype after 4 days of incubation in normal medium without TAMs-CM (Fig EV1C). Also, TAMEMs uniquely highly expressed certain genes such as *Spp1* and *Gas6*.

To further analyze the similarities and differences between TAMEMs and M2/M1 macrophages, we employed high-throughput single-cell RNA sequencing (scRNA-seq) to analyze 5,952 TAMEMs against 6,853 M1 and 7,660 M2 macrophages, detecting 3,463 genes on average in each cell. The data, aligned and projected two-dimensionally into a t-stochastic neighbor embedding (tSNE) plot, revealed distinct gene expression patterns among the three cell types (Fig 2C). The characteristics of TAMEMs could not be defined by typical M1 (CD86 and CD80) and M2 (CD206 and PPARγ) markers. TAMEMs expressed *Mrc1* (encoding CD206) highly but *Pparg* lowly, which differentiated them from a typical M2a subtype; meanwhile their modest levels of *Cd80* and *Cd86* denied their feature as M1 cells (Fig 2D). Differential gene expression (DGE) analyses and GO analyses further suggested that, in TAMEMs, the most enriched GO terms of the top 50 upregulated genes included wound healing, epithelial cell proliferation, angiogenesis, and negatively regulating inflammatory responses (Fig 2E and F). The pattern is partly comparable to that of M2 macrophages, despite with clear differences. Specifically, TAMEMs highly expressed key growth factors (e.g., *Pdgfb* and *Ang*) and a range of immunosuppressive markers—including *Mrc1*, *Pf4* (M2 polarization; Scheuerer *et al*, 2000; Gleissner *et al*, 2010), *Spp1* (M2 polarization; Lamort *et al*, 2019), *Gas6* (inflammatory resolution; Alciato *et al*, 2010; Nepal *et al*, 2019), and *Stab1* (tissue homeostasis; Rantakari *et al*, 2016; Fig EV1D). The results were consistent with the bulk RNA-seq data, suggesting TAMEMs exhibited a distinct, pro-repair and anti-inflammatory phenotype.

Further tSNE analysis helped to understand the heterogeneity of the five subsets (T-0 to T-4) in TAMEMs. Based on unsupervised Seurat clustering, the analyses singled out five clusters with differential expression profiles in TAMEMs (Fig EV1E). T-0 to T-3 clusters lowly expressed the M2 marker/*Chil3* and M1 marker/*Ccl5* and highly expressed *Gas6* (T-0), *Ccr2* (pro-inflammatory gene, T-1), *Spp1* (T-2), and *Tfrc* (iron internalization-related gene, T-3). T-4 lowly expressed M1 markers (*Ccl5* and *Nos2*) yet highly expressed *Stmn1* that functions to accelerate cell motility (Han *et al*, 2017). Then, Slingshot pseudotime analysis helped to sketch the differentiation trajectories within TAMEMs. Pseudotime results indicated a branching lineage in these five clusters with one functionally specialized original cluster (Fig EV1F and G). Both pseudotime analysis and real-time quantitative PCR (RT–qPCR) consistently indicated that the four genes representing T0 to T3 continued rising (Fig EV1H), suggesting these four clusters being terminal and developing from T-4 (Fig EV1I). This result is consistent with the two specific genes of TAMEMs (*Gas6* and *Spp1*) previously discovered in bulk RNA-seq. Understanding such phenotypic features of TAMEMs may provide a guideline for standardizing TAMEMs preparation.

Finally, we verified our hypothesis that TAMEMs partially recapitulated TAMs' reparative characteristics, by comparing their scRNA-seq data. The gene expression profiles of these two cells were similar (Fig 2G), with a high correlation (Pearson $r = 0.89$, Pearson's correlation; Fig 2H). Gene set variation analysis (GSVA) further revealed the functional similarity and difference between TAMs and TAMEMs. Both cells highly expressed genes regulating inflammatory resolution and tissue regeneration. However, TAMs have gene sets enriched in other GO terms involved in inflammatory resolution and tissue regeneration (e.g., positive regulation of IL-10 and VEGF) and the glycolytic process owing to the hypoxic tumor microenvironment. Meanwhile, TAMEMs contain gene sets correlated with GO terms and nonenriched in TAMs (e.g., cholesterol metabolic process; Fig 2I). Meanwhile, DGE analysis revealed that, compared with TAMEMs, the differentially expressed genes in TAMs were mainly related to

Figure 2. TAMEMs exhibit an anti-inflammatory and pro-healing phenotype and partially recapitulate the functions of TAMs.

A MA plot for DGE analysis between TAMEMs and M0, M1, and M2, respectively. Points that are significantly upregulated with an fdr < 0.05 are in pink, significantly downregulated with an fdr < 0.05 are in blue, and all others are in gray. *P*-values for selected genes: TAMEMs versus M0 (*Ccl8*-8.42E-12, *Ccl12*-3.63E-26, *Igfbp6*-3.39E-17, *Socs3*-5.78E-52, *Arg1*-6.75E-11, *Spp1*-8.06E-12, *Tgfbi*-9.26E-29, *Mrc1*-0.00226357, *Ang*-0.00000982, *Pdgfc*-0.000181909), TAMEMs versus M1 (*Mrc1*-6.27E-29, *Ccr2*-8.77E-16, *Igfbp4*-3.11E-125, *Fgf1*–0.000452031, *Pdgfc*-8.65E-25, *Gas6*-4.81E-80, *Spp1*-5.13E-91, *Hgf*-2.91E-14, *Il10*-0.0000101, *Ang*-3.85E-26), TAMEMs versus M2 (*Cx3cr1*–5.1E-18, *Saa3*-5.1E-54, *Igfbp6*-0.000000145, *Gas6*-2.64E-11, *Il10*-0.001405805, *Il16*-0.000000283, *Pdgfc*-0.0000334, *Spp1*-0.0000334, *Pf4*-0.0000388, *Vegfb*-0.046033485).

B Heatmap of differentially expressed immune-related and growth factor transcripts between TAMEMs, M2, and M0 cells, as determined using bulk RNA-seq analysis.

C t-Stochastic neighbor embedding (tSNE) representation of aligned gene expression data in single cells of TAMEMs ($n = 5{,}952$), M2 ($n = 7{,}660$), and M1 ($n = 6{,}853$), each dot represents a single cell.

D Gene expression patterns projected onto tSNE plots of *Cd86*, *Cd80*, *Mrc1*, and *Pparg* (scale: log-transformed gene expression).

E, F Heatmap of the top 50 upregulated genes (E) and top six enriched gene ontology (GO) terms (F) in each macrophage samples classified by Seurat unbiased clustering.

G Gene expression heatmap of complete transcriptome genes in TAMs and TAMEMs.

H Correlation of TAMs with TAMEMs, each dot represents a gene expression (Pearson's correlation analysis).

I Gene set variation analysis (GSVA) and representative GO terms of TAMs and TAMEMs.

© 2022 The Authors

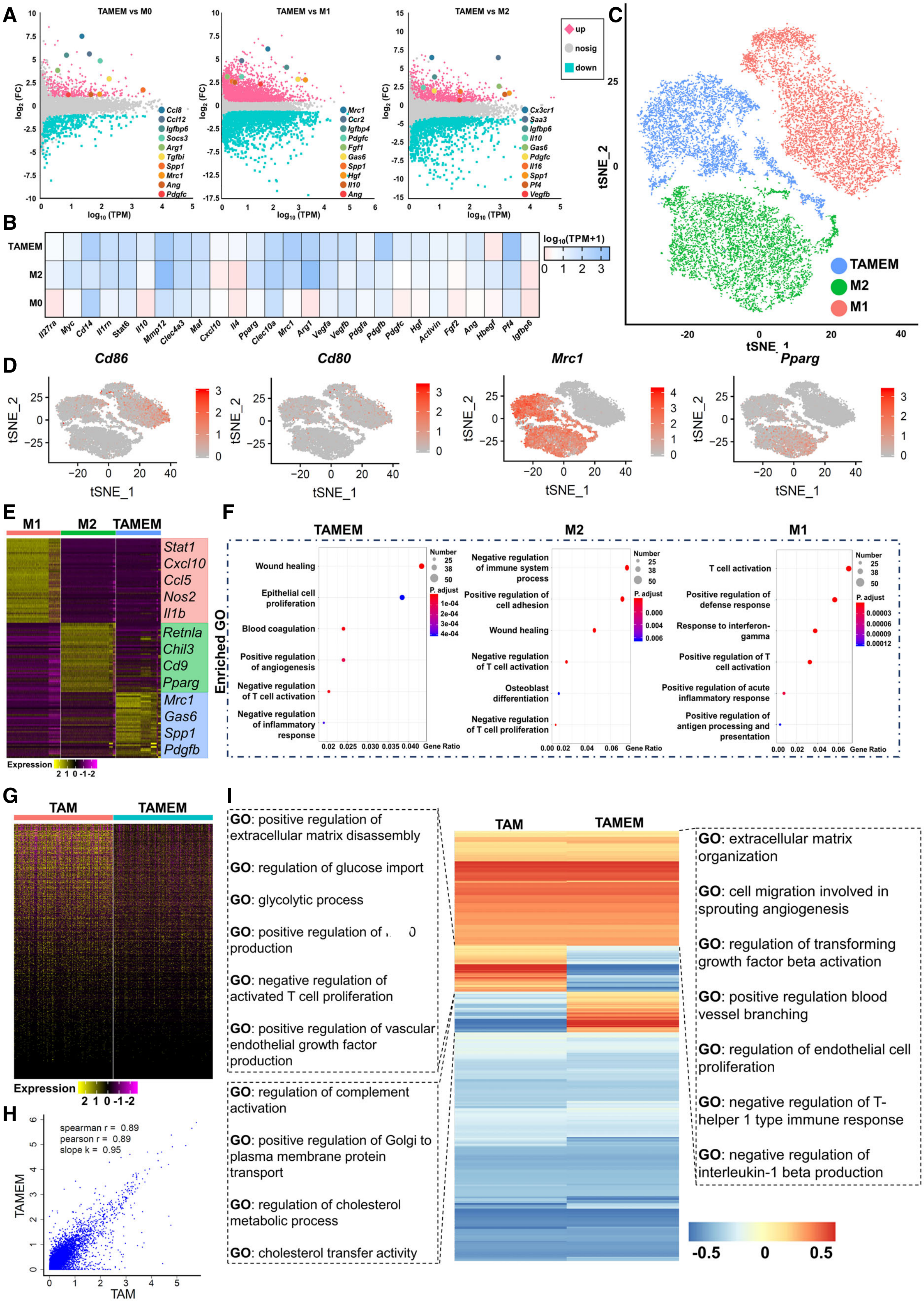
A
TAMEM vs M0
TAMEM vs M1
TAMEM vs M2
up
nosig
down
log2 (FC)
log10 (TPM)
Ccl8
Ccl12
Igfbp6
Socs3
Arg1
Tgfbi
Spp1
Mrc1
Ang
Pdgfc
Mrc1
Ccr2
Igfbp4
Pdgfc
Fgf1
Gas6
Spp1
Hgf
Il10
Ang
Cx3cr1
Saa3
Igfbp6
Il10
Gas6
Pdgfc
Il16
Spp1
Pf4
Vegfb
B
TAMEM
M2
M0
log10(TPM+1)
Il27ra
Myc
Cd14
Il1rn
Stat6
Il10
Mmp12
Clec4a3
Maf
Cxcl10
Il4
Pparg
Clec10a
Mrc1
Arg1
Vegfa
Vegfb
Pdgfa
Pdgfb
Pdgfc
Hgf
Activin
Fgf2
Ang
Hbegf
Pf4
Igfbp6
C
tSNE_2
tSNE_1
TAMEM
M2
M1
D
Cd86
Cd80
Mrc1
Pparg
tSNE_2
tSNE_1
E
M1
M2
TAMEM
Stat1
Cxcl10
Ccl5
Nos2
Il1b
Retnla
Chil3
Cd9
Pparg
Mrc1
Gas6
Spp1
Pdgfb
Expression
F
TAMEM
M2
M1
Enriched GO
Wound healing
Epithelial cell proliferation
Blood coagulation
Positive regulation of angiogenesis
Negative regulation of T cell activation
Negative regulation of inflammatory response
Negative regulation of immune system process
Positive regulation of cell adhesion
Wound healing
Negative regulation of T cell activation
Osteoblast differentiation
Negative regulation of T cell proliferation
T cell activation
Positive regulation of defense response
Response to interferon-gamma
Positive regulation of T cell activation
Positive regulation of acute inflammatory response
Positive regulation of antigen processing and presentation
Number
P. adjust
Gene Ratio
G
TAM
TAMEM
Expression
H
spearman r = 0.89
pearson r = 0.89
slope k = 0.95
TAMEM
TAM
I
GO: positive regulation of extracellular matrix disassembly
GO: regulation of glucose import
GO: glycolytic process
GO: negative regulation of activated T cell proliferation
GO: positive regulation of vascular endothelial growth factor production
GO: regulation of complement activation
GO: positive regulation of Golgi to plasma membrane protein transport
GO: regulation of cholesterol metabolic process
GO: cholesterol transfer activity
TAM
TAMEM
GO: extracellular matrix organization
GO: cell migration involved in sprouting angiogenesis
GO: regulation of transforming growth factor beta activation
GO: positive regulation blood vessel branching
GO: regulation of endothelial cell proliferation
GO: negative regulation of T-helper 1 type immune response
GO: negative regulation of interleukin-1 beta production
-0.5
0
0.5

Figure 2.

glycolysis and tumor metastasis (Fig EV1J). Also, the Ly6C expression in the TAMs scRNA-seq results (Fig EV1K) was consistent with the flow cytometry results (Appendix Fig S1A). These data highlighted the similarity between TAMEMs ("the trainee") and TAMs ("the trainer") in reparative and anti-inflammatory phenotype and indicated their difference in gene expression that is understandable according to their different origins.

TAMEMs mediate inflammatory resolution and stimulate angiogenesis and fibroblast growth *in vitro*

The above data revealed the multifacet pro-healing and anti-inflammatory phenotype of TAMEMs. To validate the results of bioinformatics analysis at the protein level, we used a membrane-based antibody array to detect 78 soluble proteins (including inflammatory factors, chemokines, and growth factors) secreted by TAMEMs (Fig EV2A and B). We selected 22 proteins with significant differences and determined their absolute concentrations using the Luminex Assays system. As shown in Fig 3A, TAMEMs not only secreted a comparable amount of IL-4, but abundantly produced more other immunosuppressive cytokines/chemokines than did M2 macrophages. These included IL-6—known for its both pro- and anti-inflammatory effects but being particularly immunosuppressive in the tumor niche (Liu *et al*, 2017), CXCL1 that is typically overexpressed in tumors to induce myeloid-derived suppresser cells (Hu *et al*, 2021), and IL-16 and CXCL12 known for switching macrophage M2 polarization (Dürr *et al*, 2010; Babazadeh *et al*, 2021). There was also elevated expression of multiple pro-angiogenic cytokines in TAMEMs, including bFGF, PDGF-AA, and VEGF (Fig 3A). These results demonstrated the potential of TAMEMs to promote tissue regeneration, and we continued to examine their functions in cell models *in vitro*.

First, we examined the capability of TAMEMs in resolving inflammation. We treated primary BMDMs with conditional medium (CM) from TAMEMs and typical M0, M1, or M2 macrophages. Flow cytometry analysis, IF staining, and qPCR consistently indicated that the treatment with TAMEMs-CM for 48 h upregulated the expression of M2 markers (CD206 and Arg1) and downregulated the expression of M1 markers (CD86, Il-6, and Nos2) in BMDMs (Fig 3B–D). Notably, TAMEMs-CM was more potent than M2-CM in inducing M2 polarization in BMDMs. Besides immunocytes, fibroblasts' dysfunction also correlates with the prolonged inflammation in diabetic wounds (Wall *et al*, 2008). Thus, we analyzed the influence of TAMEMs on fibroblasts prechallenged with lipopolysaccharide (LPS). Treatment with TAMEMs-CM significantly reduced *Il6* and *Cxcl15* expression in the fibroblasts—to an extent even lower than those treated by M2-CM, via suppressing IκB phosphorylation (Fig 3E–G). These findings validated TAMEMs' capability to resolve inflammation through paracrine signaling.

Then, we assessed the effects of TAMEMs on the proliferation of key stromal cells during wound healing. Expectedly, TAMEMs-CM displayed the highest potency in (i) inducing endothelial (SVEC4-10) tube formation in a Matrigel assay, comparable to that of recombinant VEGF (Fig 3H) and (ii) stimulating fibroblasts (L929) migration in a scratch assay, closing the wound gap over 80% compared with the control group (Fig 3I and J). In addition, TAMEMs-CM-treated fibroblasts proliferated the fastest (over 300% at 72 h; Fig 3K). Further analyses by qPCR (Fig 3L), Western blotting (WB, Fig 3M and N), and IF staining (Fig 3O) consistently demonstrated that TAMEMs-CM most significantly upregulated the expression of α-SMA (encoded by *Acta2*) and type I collagen (encoded by *Col1a1*), two representative markers for activated fibroblasts. These findings, in agreement with the cytokine array data, collectively highlighted the multifacet, pro-healing functions of TAMEMs. In addition,

Figure 3. TAMEMs resolve inflammation and stimulate angiogenesis and fibroblast growth *in vitro*.

- A Quantification of concentrations of 22 soluble proteins from the cytokine and angiogenesis array analysis (**P* < 0.05, ****P* < 0.001, and *****P* < 0.0001; ns: not significant; *n* = 3, biological replicates).
- B, C Flow cytometry analysis (B) and IF staining (C) for CD86 and CD206 in BMDMs that were cultured with conditional medium (CM) from TAMEMs, M2, M1, M0, and normal medium for 48 h (In (B), *****P* < 0.0001; In (C), large and small images are derived from the same cell, the former show the CD86/CD206 channel, while the latter show the merger of F4/80 and CD86/CD206, scale bar: 20 μm; *n* = 6, biological replicates).
- D The real-time qPCR analysis of macrophage polarization genes (M1: *Nos2* and *Il6*; M2: *Arg1* and *Mrc1*) in BMDMs treated with CMs from TAMEMs and other controls for 48 h (**P* < 0.05, ****P* < 0.001, *****P* < 0.0001, and ns: not significant (*P* > 0.05) vs. the normal medium group; *n* = 6, biological replicates).
- E The real-time qPCR analysis of pro-inflammatory cytokines (*Il6* and *Cxcl15*) in fibroblasts (L929) that pretreated with LPS and then cultured with CMs from TAMEMs and other controls for 24 h (**P* < 0.05, ***P* < 0.01, ****P* < 0.001, *****P* < 0.0001, and ns: not significant (*P* > 0.05) vs. the LPS-treated group; *n* = 6, biological replicates).
- F, G Western Blotting analysis of phosphorylated IκB (F) and quantification of gray value (G) in fibroblasts (L929) that pretreated with LPS and then cultured with CMs from TAMEMs and other controls for 24 h (**P* < 0.05, ***P* < 0.01, *****P* < 0.0001, and ns: not significant (*P* > 0.05) vs. the LPS-treated group; *n* = 3, biological replicates).
- H Representative images and quantification of the Matrigel-based angiogenesis assay in mouse vascular endothelial cell (SVEC4-10) treated with CMs from TAMEMs and other controls, VEGF treatment as a positive control (*****P* < 0.0001 and ns: not significant (*P* > 0.05) vs. the normal medium group; scale bar: 100 μm; *n* = 6, biological replicates).
- I Scratch wound assay of L929 cells treated with different CMs for 4 and 8 h (scale bar: 200 μm; *n* = 6).
- J Quantification of the remaining scratch area was performed using an image analysis, the rates are presented as a percentage of the initial scratch area at 0 h (**P* < 0.05, ***P* < 0.01, ****P* < 0.001, *****P* < 0.0001, and ns: not significant (*P* > 0.05) vs. the normal medium group; *n* = 6, biological replicates).
- K The proliferation rate of L929 cells pretreated with different CMs was analyzed using the CCK-8 kit (**P* < 0.05, ***P* < 0.01, *****P* < 0.0001, and ns: not significant (*P* > 0.05) vs. the normal medium group; *n* = 6, biological replicates).
- L–O The real-time qPCR analysis (L), Western Blotting (M) (including quantification of gray value (N)), and IF staining (O) for α-SMA and type I Collagen in L929 cells after 48-h treatment with different CMs (**P* < 0.05, ***P* < 0.01, ****P* < 0.001, *****P* < 0.0001, and ns: not significant (*P* > 0.05) vs. the normal medium group, scale bar: 10 μm; *n* = 3, biological replicates).

Data information: Data represent means ± SD. The differences between groups were analyzed using ordinary one-way ANOVA with Tukey's multiple comparison test in (D, H) and two-way ANOVA with Tukey's multiple comparison test in (A, B, E, G, J–L, N) in GraphPad Prism 8.

 © 2022 The Authors

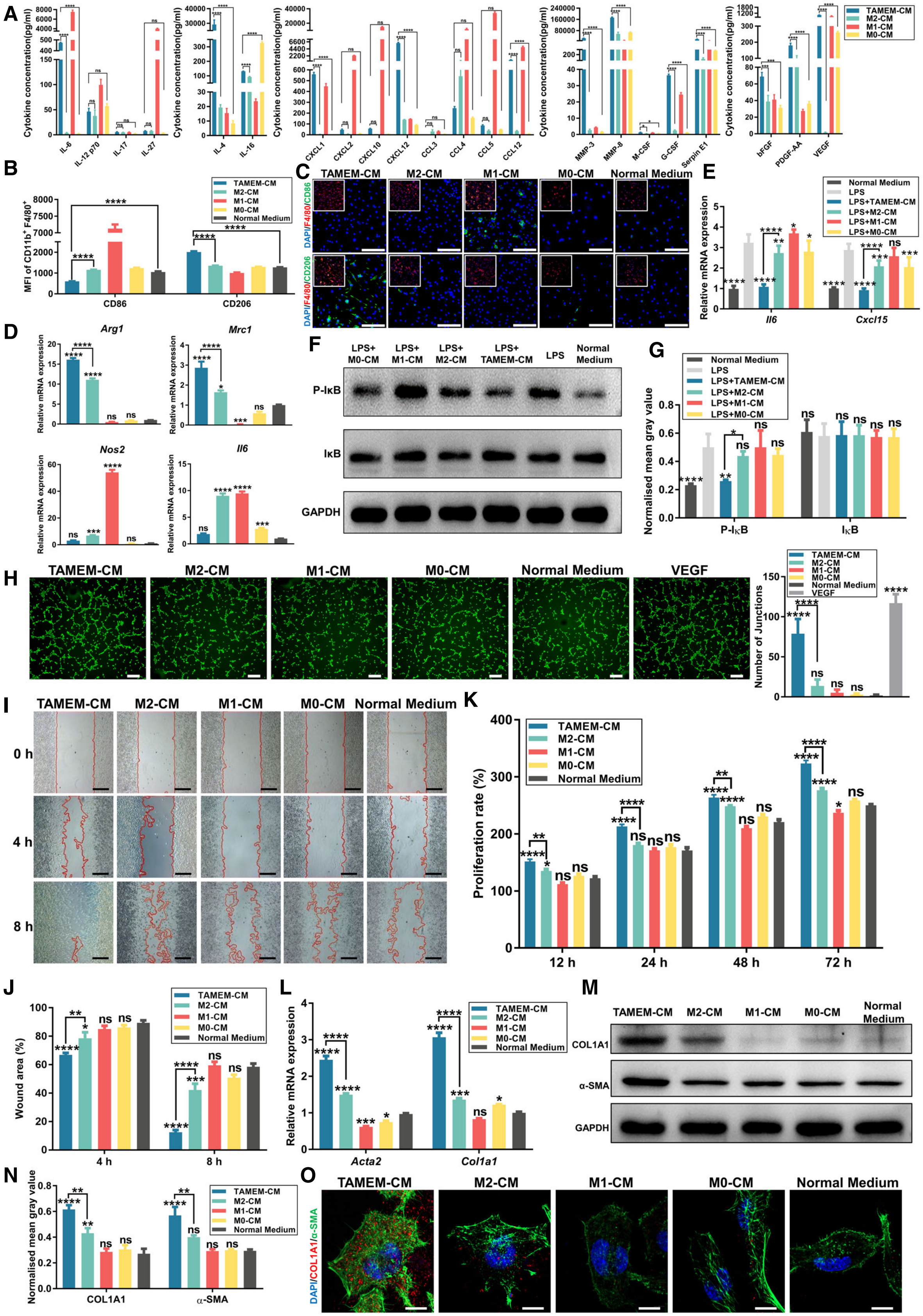
A
B
C
D
E
F
G
H
I
J
K
L
M
N
O
TAMEM-CM
M2-CM
M1-CM
M0-CM
Normal Medium
LPS
VEGF
Cytokine concentration(pg/ml)
MFI of CD11b+ F4/80+
CD86
CD206
Relative mRNA expression
Arg1
Mrc1
Nos2
Il6
Cxcl15
P-IκB
IκB
GAPDH
Normalised mean gray value
Number of Junctions
0 h
4 h
8 h
Proliferation rate (%)
12 h
24 h
48 h
72 h
Wound area (%)
Acta2
Col1a1
COL1A1
α-SMA
DAPI/COL1A1/α-SMA

Figure 3.

TAMs-CM was stronger than TAMEMs-CM in inducing inflammation resolution and fibroblast activation (Fig EV2C and D).

TAMEMs accelerate healing of skin wounds in both type I and II diabetic mice

We next tested whether TAMEMs could exert their immunomodulatory and pro-regenerative functions to promote diabetic wound healing *in vivo*. As illustrated in Fig 4A, we prepared a biomaterial scaffold to deliver TAMEMs to the wound bed. Using electrospinning, we fabricated gelatin—an FDA-approved biomacromolecule with high biocompatibility—into porous, fibrous scaffolds that facilitate the adhesion of TAMEMs. The scaffolds had ~62% porosity and regular fibers of ~1 μm diameter (Appendix Fig S2A) and formed transparent gels when equilibrated in an aqueous solution (Fig 4B). Loading of TAMEMs onto the scaffolds did not affect their morphology (Fig 4C), proliferation (Appendix Fig S2B), or polarization (Appendix Fig S2C) *in vitro*.

Meanwhile, we created a full-thickness excisional splinted wound in streptozotocin (STZ)-induced type I diabetic (T1D) mice (Brem & Tomic-Canic, 2007; Tan & Wahli, 2013; Hu *et al*, 2017), with the level of blood glucose monitored through 15 days after STZ injection (Appendix Fig S2D). Then, we applied the scaffolds laden with the same number of TAMEMs, M0, M1, or M2 cells, which were isolated from C57BL/6 eGFP$^+$ mice (M0) followed by different inductions (others), to the site of diabetic wounds (3×10^5 cells per scaffold; one scaffold per wound). In addition to these cell controls and blank control, we employed an extra control group, in which normal, nondiabetic mice were created with wounds in the same way but received no treatment. This group offered a "normal" healing scenario for comparison. The scaffolds laden with any macrophage group did not affect the level of blood glucose or weight of the experimental mice (Appendix Fig S2E).

Interestingly, TAMEMs maintained the highest viability after grafting, with 130 eGFP$^+$ cells (per 10^5 of all tissue cells from the wound site) viable at day 5, when other groups of macrophages declined to 15 (M2), 3 (M1), and 0 (M0), respectively (Appendix Fig S2F), which correlated with the earlier *in vitro* findings that TAMEMs survived longer than other groups (Fig 1C). Meanwhile, the transplanted TAMEMs gradually migrated to the granulation tissue of the wound (Appendix Fig S2F). To analyze the stability of TAMEMs' phenotype post-transplantation *in vivo*, we detected CD206 and CD86 in the cultured cells (day 0) and those at day 3 post-transplantation by flow cytometry (Appendix Fig S2G: gating strategy; Appendix Fig S2H: histogram; Appendix Fig S2I: MFI statistics). Further, we measured the expression of *Mrc1*, *Arg1*, *Cd86*, and *Nos2* of the cultured cells (day 0) and those isolated from the wounds by FACS at day 3 via qPCR (Fig 4D). These data revealed that TAMEMs maintained anti-inflammatory phenotype even in a severely inflammatory diabetic wound environment, whereas M2 and M0 macrophages significantly upregulated the expression of pro-inflammatory markers (*Cd86* and *Nos2*).

We evaluated the healing outcomes in various aspects. First, the gross view over 21 days showed that TAMEMs-laden scaffolds induced wound closure faster than any other group in diabetic mice, at a comparable rate to that in normal mice (Fig 4E). Second, the histological staining (H&E) of wound sections showed increased healing in the TAMEMs-treated samples, the only group with more than 80% wound closure at day 21 besides the normal mice (Figs EV3A and 4F). It also revealed thickening of the granulation tissue only in the TAMEMs-treated group and normal mice on day 7, when other diabetic groups hardly presented granulation tissue (Fig 4G and H). At day 14, TAMEMs induced a larger area of re-epithelialization (Fig 4G and I) than did other macrophages in diabetic wounds. Third, Masson's trichrome staining revealed more collagen deposition in TAMEMs-treated wound tissues than in other diabetic groups at day 14 (Fig 4G and J). Finally, TAMEMs induced the highest expression of *Col1a1* and *Tgfb1* at the wound sites at day 14 (Fig 4K). Consistently, among all diabetic mice, the highest number of fibroblasts appeared in the wound site of the TAMEMs group, according to flow cytometric analysis for vimentin and CD140a (PDGFR; Fig 4L and M; with gating strategy in Appendix Fig S3D).

Next, we validated the effect of TAMEMs on a type 2 diabetic wound model. Similar to the conditions performed in the T1D mice,

Figure 4. TAMEMs accelerate the healing of skin wounds in both STZ-induced T1D mice. ▶

- A Schematic diagram of the transplantation of TAMEMs and other control cells.
- B Representative physical images of gelatin electrospinning scaffolds in the dry and wet state.
- C Representative SEM images of TAMEMs loaded in gelatin electrospinning scaffolds (scale bar: 20 and 4 μm).
- D Quantification of the expression of macrophage polarization-related genes (pro-inflammatory: *Nos2* and *Cd86*; anti-inflammatory: *Arg1* and *Mrc1*) to compare cultured cells (before transplantation) and those isolated from wounds after 3 days transplantation (after transplantation) (**$P < 0.01$, ****$P < 0.0001$, and ns: not significant ($P > 0.05$); $n = 3$, biological replicates).
- E Representative images of the wound closure process were captured during the 21-day *in vivo* experiments (scale bar: 500 μm; $n = 8$, biological replicates).
- F The percentage of wound closure determined at each time point (****$P < 0.0001$ and ns: not significant ($P > 0.05$) vs. the blank control group; $n = 8$, biological replicates).
- G H&E (day 7 and 14) and Masson's (day 14) staining for full-thickness skin samples containing the entire wound area (scale bar: 500 μm; $n = 8$), the under images are the higher magnification images that indicate an area in the red boxed region in day 7 and 14 (scale bar: 100 μm; $n = 8$, biological replicates) (gt: granulation tissue, he: hyperproliferative epithelium, black arrow: wound edges).
- H–J Quantitative analysis of regenerated granulation (H) on day 7 and Re-epithelialization of wound bed (I) and Masson's trichrome-stained tissue (J) on day 14 (blue for collagen) (***$P < 0.001$, ****$P < 0.0001$, and ns: not significant ($P > 0.05$) vs. the blank control group; $n = 8$, biological replicates).
- K The real-time qPCR analysis of *Cola1* and *Tgfb1* mRNAs in different wound tissues at day 14 postwounding (**$P < 0.01$, ****$P < 0.0001$, and ns: not significant ($P > 0.05$) vs. the blank control group; $n = 3$, biological replicates).
- L, M Representative flow cytometry data (L) and quantification of vimentin and CD140a-positive cells (M) in different wound tissues at day 14 (*$P < 0.05$, ****$P < 0.0001$, and ns: not significant ($P > 0.05$) vs. the blank control group; $n = 8$, biological replicates).

Data information: Data represent means ± SD. The differences between groups were analyzed using two-way ANOVA with Tukey's multiple comparison test in (D, F, K) and ordinary one-way ANOVA with Tukey's multiple comparison test in (H–J, M) in GraphPad Prism 8.

© 2022 The Authors

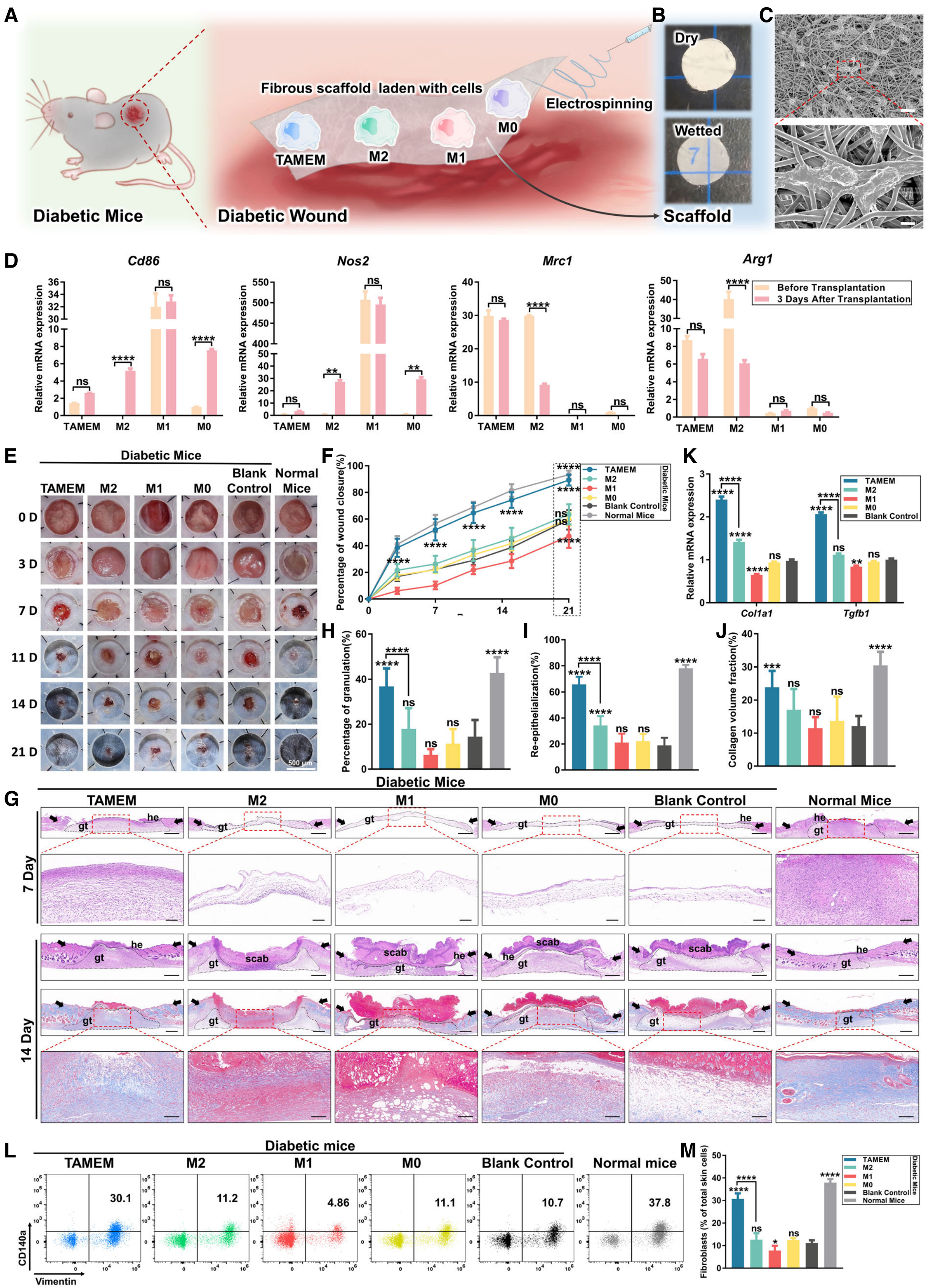
A
Diabetic Mice
Fibrous scaffold laden with cells
TAMEM
M2
M1
M0
Electrospinning
Diabetic Wound
B
Dry
Wetted
Scaffold
C
D
Cd86
Nos2
Mrc1
Arg1
Relative mRNA expression
Before Transplantation
3 Days After Transplantation
E
Diabetic Mice
TAMEM M2 M1 M0 Blank Control Normal Mice
0 D
3 D
7 D
11 D
14 D
21 D
500 μm
F
Percentage of wound closure(%)
TAMEM
M2
M1
M0
Blank Control
Normal Mice
Diabetic Mice
K
Col1a1
Tgfb1
H
Percentage of granulation(%)
I
Re-epithelialization(%)
J
Collagen volume fraction(%)
Diabetic Mice
G
TAMEM M2 M1 M0 Blank Control Normal Mice
7 Day
14 Day
gt
he
scab
L
Diabetic mice
TAMEM M2 M1 M0 Blank Control Normal mice
30.1
11.2
4.86
11.1
10.7
37.8
CD140a
Vimentin
M
Fibroblasts (% of total skin cells)

Figure 4.

we created full-thickness splint wounds in type II diabetic (T2D) BKS-Lepr^{em2Cd479}/Gpt mice (db/db) and applied the scaffolds with different macrophage types. Histological staining of wound sections at 11 and 21 days highlighted the fastest healing in the TAMEMs-treated group, comparable to the normal mice (Fig EV3B, quantification in Fig EV3C).

The above data suggested that TAMEMs delivered in biomatrix scaffolds could efficiently and comprehensively promote the repair of diabetic wounds in both T1D and T2D mouse models. Their reparative functions outperformed other macrophage phenotypes—notably including M2 that is conventionally associated with repairing activities.

TAMEMs resolve inflammation in diabetic wounds

We set out to verify whether the encouraging healing performance of TAMEMs was owing to their restoration of immune balance, since disrupted immune balance is a fundamental pathological factor underlying the nonhealing state of diabetic wounds (Boniakowski *et al*, 2017; Kimball *et al*, 2018). We analyzed the phenotypes of local host macrophages after the implantation of various macrophage types with scaffolds in the T1D mice. To distinguish the transplanted and host macrophages, we specifically obtained the cells for transplantation—TAMEMs, M0, M1, and M2 macrophages—from C57BL/6 eGFP$^+$ mice. Having removed doublets, nonviable cells, and eGFP-labeled cells, we analyzed the expression of CD86 and CD206 in CD11b$^+$F4/80$^+$ macrophages, and the proportion of CD11b$^+$Ly6C^{Hi} monocyte/macrophages (Appendix Fig S3A) in the wound tissues at day 1, 3, and 7 postinjury with flow cytometry.

For pro-inflammatory CD86$^+$ macrophages, they infiltrated the wounds in a relatively high number in all groups by day 1, especially in the M1-treated group, possibly due to increased inflammatory factors and chemokines, and their proportion gradually decreased through day 7 (Fig 5A and Appendix Fig S3B). Their decrease was the most obvious in the TAMEMs group (except for normal mice) from day 1 to 3, suggesting a stronger reversing effect of these cells than other macrophages. For anti-inflammatory CD206$^+$ macrophages, they existed in low numbers in all groups at day 1, but sharply increased in the wounds of TAMEMs-treated group and normal mice from day 1 to 7 (Fig 5B and Appendix Fig S3B). Such a trend revealed a direct and potent effect of TAMEMs in inducing local host macrophages into a resolving phenotype. Notably, delivery of M2 macrophages failed to exhibit such an effect and showed no significant difference from implantation of M0 cells or no treatment in inducing anti-inflammatory macrophages. Besides, in the groups of diabetic mice, only TAMEMs kept suppressing the number of Ly6C^{Hi} inflammatory monocytes through the observation period (~5.61% at day 3; Fig 5C and Appendix Fig S3C), whereas other groups rapidly increased from day 1 to 3 (M2: from ~9.37 to ~12.8%; M1: from ~12.1 to ~14.5%; M0: from ~9.45 to ~12.1%; control: from ~9.3 to ~13.3%). This finding indicated the efficacy of TAMEMs in blocking the second wave of Ly6C^{Hi} infiltration, which is a crucial pathology for the nonhealing status of diabetic wounds (Kimball *et al*, 2018).

Considering the complex macrophage phenotype *in vivo* (Ma *et al*, 2022), we analyzed more genes to demonstrate the immunosuppressive capacity of TAMEMs. First, as shown in Appendix Fig S3E and F, at day 7 postwounding, TAMEMs increased the number of both CCR2$^+$ and CD163$^+$ macrophages, which are critical for angiogenesis (Boniakowski *et al*, 2018) and wound healing (Ferreira *et al*, 2020) but both lacking in diabetic wounds. In addition to these two cell surface markers, we measured the expression of four genes, including *Il1b* and *Ptgs*, whose expression peaks in earlier stages of healing but decreases later, and *Mgl2* and *Clec10a*, which have an opposite trend of expression (Willenborg *et al*, 2021), in macrophages at day 7 postwounding following flow cytometry sorting. As shown in Appendix Fig S3G, TAMEMs decreased the expression of *Il1b* and *Ptgs* in macrophages and increased that of *Mgl2* and *Clec10a*, which are lowly expressed in diabetic wound macrophages.

The IF staining outcomes were consistent about the presence of pro-inflammatory (F4/80$^+$/CD86$^+$) and anti-inflammatory (F4/80$^+$/CD206$^+$) cells in different groups. In TAMEMs-treated wound tissues, the number of the former at day 3 was lower and that of the latter at day 7 was higher than in other diabetic groups. Also, the levels of TNF-α and IL-6, two representative pro-inflammatory cytokines, were significantly decreased in the TAMEMs-treated

Figure 5. TAMEMs resolve inflammation and rebuild vasculature in diabetic wounds.

A, B The MFI of pro-inflammatory macrophages (CD86-positive cells) (A) and anti-inflammatory macrophages (CD206-positive cells) (B) gating from F4/80$^+$ macrophages on postinjury days 1, 3 and 7 (*$P < 0.05$, **$P < 0.01$, ****$P < 0.0001$, and ns: not significant ($P > 0.05$) vs. the blank control group; $n = 5$, biological replicates).

C Ly6C^{Hi} monocytes plotted as a percentage of live cells on postinjury days 1, 3 and 7 (****$P < 0.0001$ and ns: not significant ($P > 0.05$) vs. the blank control group; $n = 5$, biological replicates).

D Quantification of the expression of the angiogenesis-associated *Vegfa* and *Pdgfb* mRNAs in different wound tissues at day 7 postwounding (**$P < 0.01$, ****$P < 0.0001$, and ns: not significant ($P > 0.05$) vs. the blank control group; $n = 3$, biological replicates).

E IF staining for CD31 (red; endothelial marker, for new capillary formation) at day 7 and α-SMA (green; smooth muscle marker, for vascular maturation) at day 14, with both the entire wound region and magnified yellow-box zone (scale bar: 100 μm; $n = 8$, biological replicates) (gt: granulation tissue, he: hyperproliferative epithelium).

F Quantification of the CD31$^+$ vessels at day 7 and CD31$^+$ α-SMA$^+$ vessels and CD31$^+$ α-SMA$^-$ vessels at day 14 (****$P < 0.0001$ and ns: not significant ($P > 0.05$) vs. the blank control group; $n = 8$, biological replicates).

G Representative multiphoton projection images of blood vessels (lectin) in healed skin wound tissue after the administration of different treatments at day 21 postinjury, uninjured skin was analyzed as positive control. Up: the images of the entire sampling area (yellow circled) containing the healed wound (green circled); Down: magnified zone (blue boxed in the upper images; scale bar: 100 μm; $n = 3$, biological replicates).

H, I Orientational distribution (H) and quantification of blood vessel alignment (I), based on pixel orientation (***$P < 0.001$, ****$P < 0.0001$ and ns: not significant ($P > 0.05$) vs. the blank control group; $n = 3$, biological replicates).

Data information: Data represent means ± SD. The differences between groups were analyzed using two-way ANOVA with Tukey's multiple comparison test in (A–D) and ordinary one-way ANOVA with Tukey's multiple comparison test in (F, I) in GraphPad Prism 8.

© 2022 The Authors

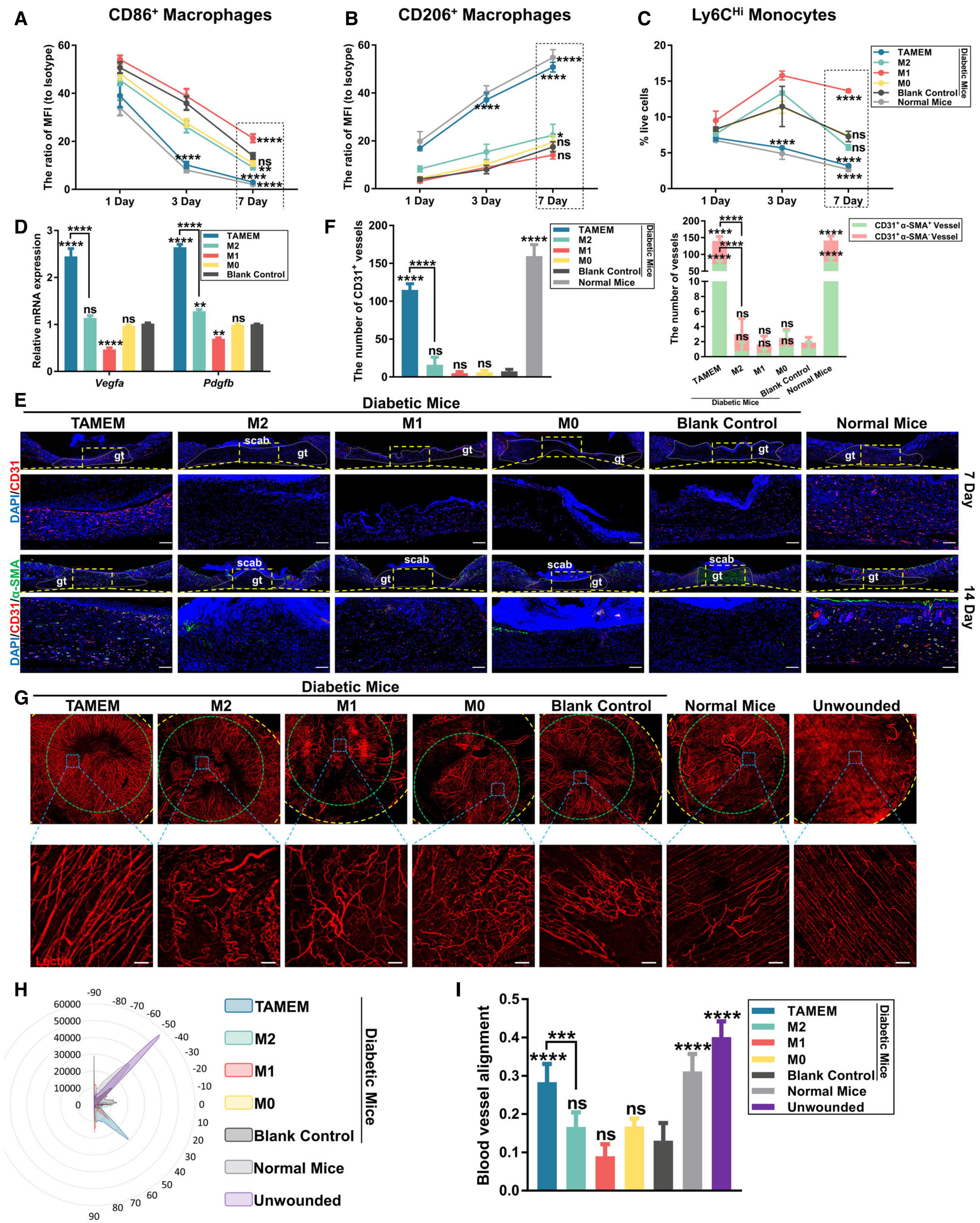

A
CD86+ Macrophages
The ratio of MFI (to Isotype)
1 Day
3 Day
7 Day
B
CD206+ Macrophages
C
Ly6CHi Monocytes
% live cells
TAMEM
M2
M1
M0
Blank Control
Normal Mice
Diabetic Mice
D
Relative mRNA expression
Vegfa
Pdgfb
F
The number of CD31+ vessels
CD31+α-SMA+ Vessel
CD31+α-SMA- Vessel
The number of vessels
E
Diabetic Mice
DAPI/CD31
DAPI/CD31/α-SMA
7 Day
14 Day
scab
gt
G
Unwounded
Lectin
H
I
Blood vessel alignment

Figure 5.

diabetic wounds and comparable to the levels in the wounds of normal mice, indicating the modulation of the local immune niche by these cells (Appendix Fig S4A and B). These findings validated that TAMEMs could efficiently shape the constitution of local host macrophages toward M2-dominant (~80% of total F4/80$^+$, at day 7), which are consistent with the flow cytometry data. This modulation consequently suppressed inflammatory cytokine production to prevent local immune niche from being persistent inflammatory—which is a key pathological factor behind diabetic ulcer.

TAMEMs rebuild vasculature in diabetic wounds

Diabetic vasculature is largely defected by compromised angiogenesis (Okonkwo & Dipietro, 2017) and deformed capillary network (Gurevich *et al*, 2018)—both related to macrophage dysfunction. Therefore, after verifying that TAMEMs promoted diabetic healing through modulating the local immune niche, we were interested in whether they restored vasculature in diabetic wounds.

First, two major growth factors mediating vasculature—VEGF-A on initiating angiogenesis and PDGF-B on stabilizing new capillaries—were found with significantly higher expression in TAMEMs-treated groups than in other groups (Fig 5D).

Second, IF staining revealed that TAMEMs induced a markedly higher expression of CD31 at day 7 (endothelial marker, for new capillary formation) and α-SMA (smooth muscle marker, for vascular maturation) at day 14 than other types of macrophages delivered (Fig 5E; quantification in Fig 5F). Then, we performed a vessel perfusion assay to analyze vascular morphology at day 21 postwounding (Appendix Fig S4C). The outcomes illustrated that the morphology and alignment of the blood vessels in TAMEMs-treated group were more organized and interconnected than those from other diabetic wounds, showing the tendency to resemble normal vasculature in unwounded skin (Fig 5G). The blood vessels in the TAMEMs-treated group were similar to those in the unwounded skin and more aligned than other groups, as revealed by an ImageJ orientational distribution analysis (Fig 5H) and pixel quantification (Fig 5I). These findings revealed the potency of TAMEMs in stimulating revascularization in the wounded skin tissue.

Identification of a recombinant protein cocktail to replace TAMs in generating TAMEMs

Having verified the capabilities of TAMEMs in recapitulating TAM's regenerative functions, we sought to substitute TAMs with a cocktail of recombinant proteins; thereby, we could train human macrophages into reparative TAMEMs without using any tumor-derived cells. We first analyzed the major components of TAM-CM and detected 144 proteins using a protein array (Fig EV4A and Table EV1). After excluding presumably unlikely ingredients (e.g., soluble receptors), we detected 49 soluble proteins and divided them into five categories according to their functions: pro-inflammatory factors, anti-inflammatory factors, colony-stimulating factors, chemokines, and growth factors (Fig EV4B), including osteopontin (OPN), a multifunctional cytokine with both pro-and anti-inflammatory effects (Trostel *et al*, 2018). From each category, two proteins with high abundance and established effects on macrophages were selected (only one protein presented in the category of colony-stimulating factor; Fig EV4B). Specifically, we determined the absolute concentration of these nine proteins by ELISA, including Osteopontin, IL-31 (pro-inflammatory; Kasraie *et al*, 2009), IL-10, TGF-beta 2, M-CSF, MIP-2 (CXLC2, promoting M2 macrophage differentiation; Di Mitri *et al*, 2019), CCL8 (activate CCR2 on macrophages; Chu *et al*, 2014), VEGF-B (M2 polarization; Wheeler *et al*, 2018), and bFGF (M2 polarization; Im *et al*, 2020; Fig 6A). Then, we reconstituted a cocktail comprising these nine cytokines—in the form of recombinant proteins, in separate human and murine sets, and based on the above concentration—in RPMI-1640 medium for further induction.

Besides, like the ones from S180, TAMs from other tumor models—4T1 mouse breast tumor, B16-F10 mouse melanoma, and MCF-7 human breast tumor—also expressed these nine proteins (Fig EV4C). Their levels in all TAMs were consistently higher than in BMDM-M0 but also showed differences. S180-derived TAMs presented the highest levels in most proteins, 4T1- and MCF-7-derived TAMs had comparable levels (though slightly lower in *Il31* and *CSF1*), and B16-F10-derived TAMs appeared moderately weaker—especially in producing IL-10 and VEGF-B. This pattern suggested that these factors were not exclusively produced by S180-derived

Figure 6. Preparation of a nine-factor cocktail and transplantation of TAMEMs$^{C\text{-}h}$. ▶

A The absolute concentration of the selected nine proteins by ELISA (n = 6, biological replicates).
B Gene expression heatmap of complete transcriptome genes in TAMEMs and TAMEMs$^{C\text{-}m}$, as determined using bulk RNA-seq analysis.
C Correlation of TAMEMs with TAMEMs$^{C\text{-}m}$, each dot represents a gene expression (Pearson's correlation analysis), as determined using bulk RNA-seq analysis.
D The real-time qPCR analysis of pro-inflammatory genes (*Il12*, *Tnfa*, *Ly6c*, and *Nos2*), anti-inflammatory genes (*Il10*, *Mrc1*, *Tgfb*, and *Arg1*), and growth factors (*Pdgfb*, *Vegfa*, and *Ang*) to compare TAMEMs and TAMEMs$^{C\text{-}m}$ (ns: not significant ($P > 0.05$); n = 6, biological replicates).
E The real-time qPCR analysis of M2 polarization genes (*Arg1* and *Mrc1*) in BMDMs treated with TAMEMs-CM, TAMEMs$^{C\text{-}m}$-CM, and normal medium for 48 h (****$P < 0.0001$ vs. the normal medium group; n = 6, biological replicates).
F Scratch wound assay of L929 cells treated with TAMEMs-CM, TAMEMs$^{C\text{-}m}$-CM, and normal medium for 6 h, and quantification of the remaining scratch area (the area between the two red dotted lines) was performed using an image analysis, the rates are presented as a percentage of the initial scratch area at 0 h (scale bar: 200 μm, *$P < 0.05$ and ****$P < 0.0001$ vs. the normal medium group, n = 6, biological replicates).
G The proliferation rate of L929 cells pretreated with different CMs was analyzed using the CCK-8 kit (*$P < 0.05$, **$P < 0.01$, ****$P < 0.0001$, and ns: not significant ($P > 0.05$) vs. the normal medium group; n = 6, biological replicates).
H Immunofluorescent staining for α-SMA and type I Collagen in L929 cells after 48-h treatment with different CMs (scale bar: 10 μm; n = 6, biological replicates).
I ELISA for α-SMA and type I Collagen in L929 cells after 48-h treatment with different CMs (*$P < 0.05$, **$P < 0.01$, and ****$P < 0.0001$ vs. the Normal Medium group; n = 6, biological replicates).

Data information: Data represent means ± SD. The differences between groups were analyzed using ordinary one-way ANOVA with Tukey's multiple comparison test in (E, F, I), and two-way ANOVA with Tukey's multiple comparison test in (D, G) in GraphPad Prism 8.

 © 2022 The Authors

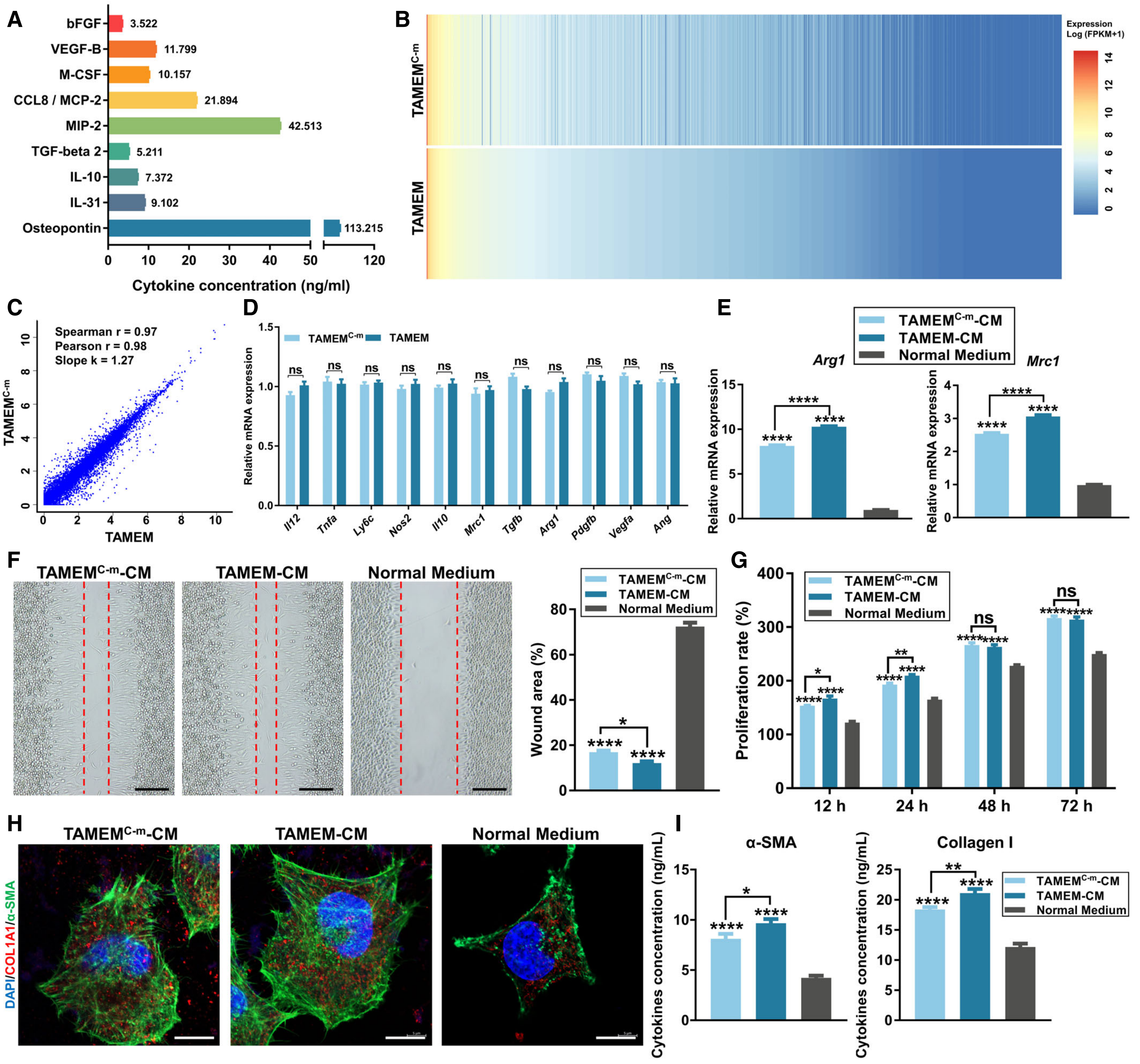

Figure 6.

TAMs. TAMs from other tumors might have a similar feature, though we chose S180 as an optimal protocol in this study.

To assess whether the recombinant protein cocktail could induce macrophages similar to TAMEMs, we cultured BMDMs in the medium containing the cocktail of nine murine recombinant proteins to prepare cocktail-educated, TAMEMs-mimicking murine macrophages (termed TAMEMs$^{C\text{-}m}$). Bulk RNA sequencing proved a high similarity between TAMEMs and TAMEMs$^{C\text{-}m}$ in gene expression (Fig 6B), with a high correlation (Pearson r = 0.98, Pearson's correlational Fig 6C) and fewer differentially expressed genes (Fig EV4D). Real-time qPCR further verified the similar levels of representative pro-inflammatory (e.g., *Il12*, *Tnfa*, and *Nos2*), anti-inflammatory (e.g., *Mrc1*, *Tgfb*, and *Arg1*), and growth factor (e.g., *Pdgfb*, *Vegfa*, and *Ang*) genes in TAMEMs$^{C\text{-}m}$ and in TAMEMs (Fig 6D). Next, TAMEMs$^{C\text{-}m}$ induced M2 polarization of macrophages (Fig 6E), drove the migration (Fig 6F) and proliferation (Fig 6G) of fibroblasts, and upregulated the expression of α-SMA and type I collagen in fibroblasts (IF: Fig 6H, ELISA: Fig 6I), as potently as did TAMEMs. These findings validated the similarity between TAMEMs$^{C\text{-}m}$ and TAMEMs and the use of the recombinant protein cocktail to replace TAM-CM for translational considerations.

In parallel, to assess the necessity of combining these nine factors, we cultured BMDMs in: (i) nine "minus" cocktails that each was ripped of one factor and (ii) nine "added" medium that each was supplemented with only one factor. We compared the morphology of TAMEMs$^{C\text{-}m}$ and the above two categories (18 types) of

induced macrophages against that of TAMEMs. The Incucytes® live-cell imaging data (Movies EV2 and EV3, with two representative time points shown in Appendix Fig S5A) and analysis of the maximum cellular diameters (Appendix Fig S5B) revealed that TAMEMs$^{C\text{-}m}$ replicated TAMEMs morphologically. Nevertheless, the removal of SPP1, MIP-2, CCL8, VEGF-B, or M-CSF protein heavily affected the morphology of the induced cells, and such influence was higher than the loss of IL-31, IL-10, TGF-β2, or bFGF protein. Also, only using any one of the nine recombinant proteins failed to induce BMDMs to exhibit the morphology of TAMEMs. Next, qPCR evaluation of the two specific TAMEMs markers (*Gas6* and *Spp1*) in the above 18 types of induced macrophages further discovered interesting phenomena (Appendix Fig S5C): (i) TAMEMs$^{C\text{-}m}$ highly expressed these two genes; (ii) removing SPP1, MIP-2, CCL8, VEGF-B, or M-CSF proteins downregulated *Gas6* expression, but M-CSF removal did not affect *Spp1* expression; (iii) stripping off IL-31 and IL-10, which had no effect on cell morphology and *Gas6* expression, markedly downregulated *Spp1* expression. Interestingly, individually adding certain cytokines can alter the expression of specific marker genes. For example, adding M-CSF or IL-10 alone upregulated *Gas 6*—but not *Spp1*—expression; adding TGF-β2 alone upregulated *Spp1*—but not *Gas6*—expression; however, removing TGF-β2 did not affect *Spp1* expression. The findings suggested that this nine-factor combination, constituted based on the TAMs' secretion, could adequately induce the desirable reparative macrophages in a cooperative, interlinked manner. The data provided evidence for the next-step preparation of human cells by human recombinant proteins.

Transplantation of "educated" human monocytes accelerates wound healing in diabetic nude mice

To assess whether the recombinant human protein cocktail could produce human macrophages functionally similar to TAMEMs, we purified human monocytes from Primary Peripheral Blood Mononuclear Cells (PBMC, StemExpress, USA) by CD14 magnetic-activated cell sorting (MACS), induced them into macrophages with human M-CSF, and further incubated them with the cocktail. These cocktail-educated, TAMEMs-mimicking human macrophages (termed TAMEMs$^{C\text{-}h}$) were seeded onto the gelatin electrospinning scaffolds and transplanted onto 6-mm full-thickness excisional splinted wounds on the dorsum of immunodeficient mice (BALB/c nude). The scheme is illustrated in Fig 7A. In parallel to serve as controls, we induced human peripheral blood macrophages into classical M1 (LPS and human recombinant IFN-γ) or M2 (human recombinant IL-4 and IL-13) phenotype for comparison.

Like TAMEMs, TAMEMs$^{C\text{-}h}$ improved healing throughout the 21-day period of observation, as demonstrated by the gross view (Fig 7B), quantification of wound closure rate (Figs EV5A and 7C), histological analysis in days 7 and 14 (H&E, Fig 7D), as well as collagen deposition (Masson's trichrome staining, Fig 7D, quantification in Fig 7E). Consistent with the findings from TAMEMs, TAMEMs$^{C\text{-}h}$ potently induced the phenotypic switch of local macrophages by decreasing CD86$^+$ population and increasing CD206$^+$ composition, while suppressing the production of pro-inflammatory cytokines in the wound milieu (Fig EV5B and C). Again, the efficacy of TAMEMs$^{C\text{-}h}$ was superior to any other types of macrophages, including M2, in all the above aspects. Further, TAMEMs$^{C\text{-}h}$ demonstrated the ability to promote angiogenesis and blood vessel maturation with high expression of CD31 and α-SMA (Fig EV5D and E). Also, they normalized the alignment of blood vessels (Fig 7F), as validated by the quantitative analysis (Figs EV5F and 7G). These findings highlighted that TAMEMs$^{C\text{-}h}$, resembling TAMEMs, performed a comprehensively reparative role in restoring local immune balance and normal vasculature, leading to desirable healing outcomes in diabetic wounds.

Finally, we wondered whether directly applying the cocktail could replace delivering live TAMEMs to heal the diabetic wounds. Likewise, we created splinted wounds in T1D mice and applied the scaffolds loaded with either TAMEMs or the cocktail. Nevertheless, both the gross view over 21 days (Appendix Fig S6A) and histological examination (Appendix Fig S6B and C) illustrated that only TAMEMs-laden scaffolds induced efficiently healing; by contrast, the cocktail group had no improvement over the control. Neither the TAMEMs transplantation nor the cocktail application changed the number of macrophages in the wound (Appendix Fig S6D), but the latter failed to induce the phenotypic switch (Appendix Fig S6E) or reverse the inflammatory markers (Appendix Fig S6F) of local macrophages in diabetic wounds. These data suggest that the cocktail is still unable to replace the trained cells and directly induce healing.

Figure 7. Transplantation of TAMEMs$^{C\text{-}h}$ promotes wound healing in immunocompromised diabetic mice. ▶

A Schematic depicting the education and transplantation of TAMEMs$^{C\text{-}h}$.

B Gross view of the wound closure process was captured during the 21-day *in vivo* experiments (scale bar: 500 μm; *n* = 8, biological replicates).

C The percentage of wound closure determined at each time point (****$P < 0.0001$ and ns: not significant ($P > 0.05$) vs. the blank control group; *n* = 8, biological replicates).

D Full-thickness skin samples containing entire wound sites were stained with H&E (day 7 and 14) and Masson (day 14; scale bar: 500 μm, *n* = 8, biological replicates), the under images are the higher magnification images that indicate an area in the red boxed region in day 7 and 14 (scale bar: 100 μm; *n* = 8, biological replicates) (gt: granulation tissue, he: hyperproliferative epithelium, black arrow: wound edges).

E Quantitative analysis of regenerated granulation on day 7 and Masson's trichrome-stained tissues on day 14 (blue for collagen) (**$P < 0.01$, ****$P < 0.0001$, and ns: not significant ($P > 0.05$) vs. the blank control group; *n* = 8, biological replicates).

F Representative multiphoton projection images of blood vessels (lectin) in healed skin wound tissue after the administration of different treatments at day 21 postinjury, uninjured skin was analyzed as positive control (Up: the images of entire sampling sites (the yellow circular region) containing healed wound sites (the green circular region); Down: the images of higher magnification that indicates an area in the blue boxed region in the upper images; scale bar: 100 μm; *n* = 3, biological replicates).

G Quantification of blood vessel alignment, based on pixel orientation (**$P < 0.01$, ****$P < 0.0001$, and ns: not significant ($P > 0.05$) vs. the blank control group; *n* = 3, biological replicates).

Data information: Data represent means ± SD. The differences between groups were analyzed using ordinary one-way ANOVA with Tukey's multiple comparison test in (E, G), and two-way ANOVA with Tukey's multiple comparison test in (C) in GraphPad Prism 8.

 © 2022 The Authors

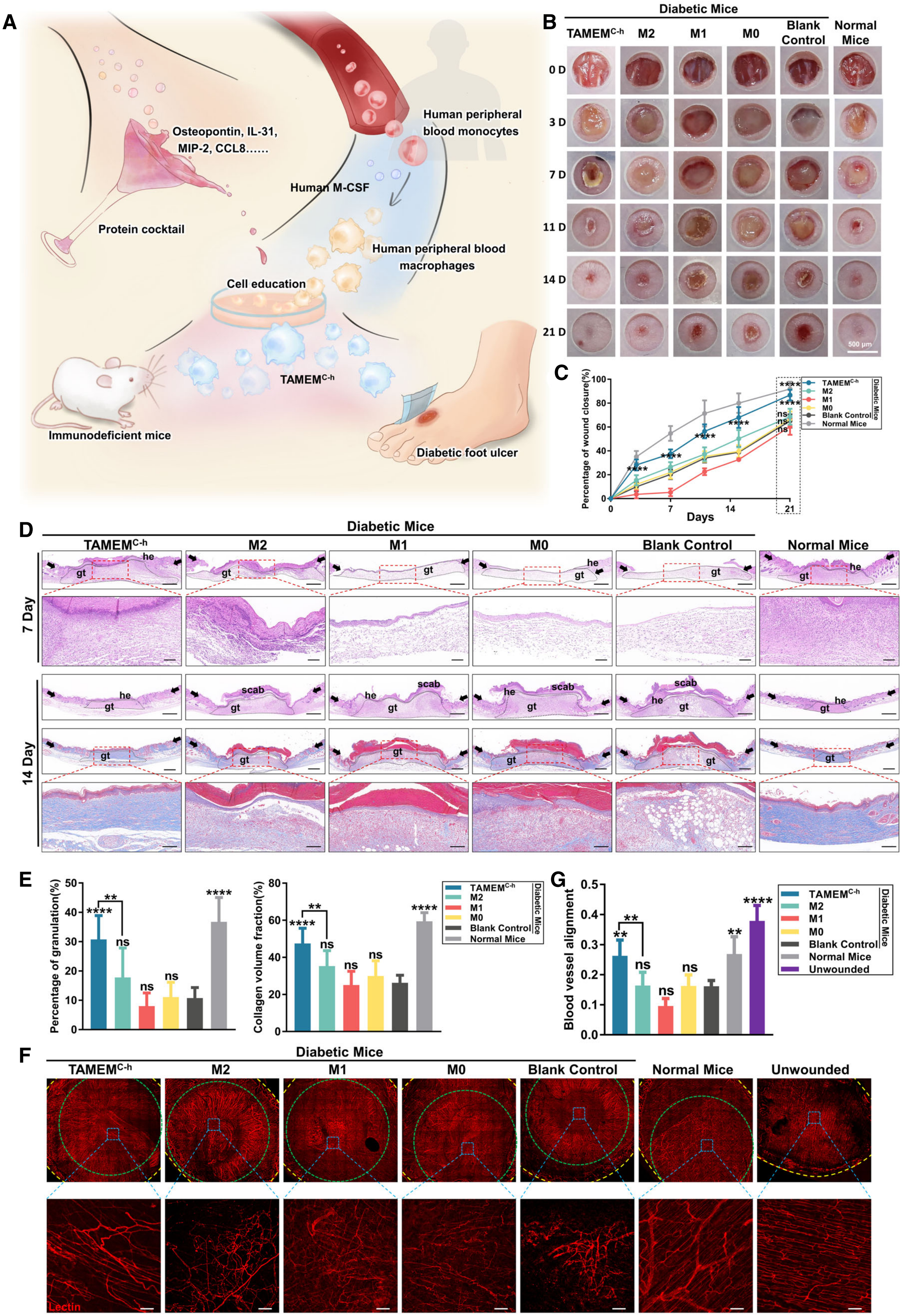
A
Osteopontin, IL-31, MIP-2, CCL8......
Protein cocktail
Human peripheral blood monocytes
Human M-CSF
Human peripheral blood macrophages
Cell education
TAMEM^C-h
Immunodeficient mice
Diabetic foot ulcer
B
Diabetic Mice
TAMEM^C-h M2 M1 M0 Blank Control Normal Mice
0 D
3 D
7 D
11 D
14 D
21 D
500 μm
C
Percentage of wound closure(%)
Days
TAMEM^C-h
M2
M1
M0
Blank Control
Normal Mice
Diabetic Mice
D
Diabetic Mice
TAMEM^C-h M2 M1 M0 Blank Control Normal Mice
7 Day
14 Day
gt
he
scab
E
Percentage of granulation(%)
Collagen volume fraction(%)
G
Blood vessel alignment
Unwounded
F
Diabetic Mice
TAMEM^C-h M2 M1 M0 Blank Control Normal Mice Unwounded
Lectin

Figure 7.

Discussion

In this study, we demonstrated the preparation of new reparative macrophage TAMEMs for diabetic wound healing. Aimed at mimicking the role of TAMs in tumor development, TAMEMs exerted TAMs' key reparative activities in murine diabetic wounds—including modulating inflammation, stimulating angiogenesis, and promoting proliferation. Since TAMEMs were "paracrine educated" by TAMs, the cytokines responsible for this education were further identified to make a cocktail of recombinant proteins, thereby replacing the use of tumor-derived cells toward higher safety. Our findings validate the possibility to recapitulate TAMs' functions in normal macrophages as a new cell therapy approach for regenerative medicine (Fig 8).

Despite progress in advanced wound care and tissue engineering (Cavanagh *et al*, 2005; Hocking, 2012), diabetic wounds remain a substantial medical challenge. Their nonhealing pathology has long been attributed to persistent inflammation (Falanga, 2005) and recently identified to be essential with macrophages—which fail to switch from inflammatory into resolving phenotypes (Sawaya *et al*, 2020). Modulating local inflammation by delivering immunocytes is possible, but questions arise on which type of cells for delivery. Danon and colleagues performed pioneering works to employ macrophages for wound healing in old mice (Danon *et al*, 1989) and human ulcers (Danon *et al*, 1997). Recent studies compared the repairing efficacies of differentially conditioned monocytes/macrophages. For example, human IL-4-induced regulatory macrophages (M2-like) promoted epithelial tissue healing in a mouse colitis model (Jayme *et al*, 2020). Nevertheless, in diabetic skin wounds, delivery of M2 macrophages either fails to promote healing (Jetten *et al*, 2014; Hu *et al*, 2017) or exhibits weaker effects than does nonpolarized M0 cells (Jetten *et al*, 2014, Hu *et al*, 2017). Other studies showed that neither classical pro- (M1) nor typical anti-inflammatory (M2) macrophages were optimal (9, 10). Even for IL-4-trained M2 macrophages that are widely regarded as reparative, the method of induction is not standardized—as another study argues that not only IL-4 (or IL-13) but also the addition of apoptotic cells is needed (Bosurgi *et al*, 2017)—and their effects vary (50). All these valuable findings suggest that the delivered cells should play comprehensive roles rather than being simply pro-/anti-inflammation (Julier *et al*, 2017). Diabetic wounds require multifunctional macrophages to modulate local inflammation, suppress the influx of the second wave of inflammatory monocytes, orchestrate vascularization, and stimulate cell proliferation. Such a comprehensive role can hardly be performed by a typical M1/M2 macrophage subtype. Intriguingly, the way TAMs shape tumor microenvironment provides an unexpected reference. Despite their heterogeneity, the main population of TAMs possess potent immunosuppressive and versatile regenerative features that precisely fulfill the requirements for the macrophages by diabetic wound healing (Franklin *et al*, 2014; Sawaya *et al*, 2020), which inspired us to devise TAMEMs.

We optimized the conditions for preparing TAMEMs based on both our previous work and preliminary tests. Our recent study found that the tumor homogenate from S180 outperformed other tumors (Hepa1-6, 4T1, and B16-F10) in their capability of immunosuppression and promoting tissue regeneration (Wang *et al*, 2020). Indeed, we later showed that TAMs from S180 expressed all the nine factors we eventually used to constitute the cocktail. Since TAMs are a major part of stromal cells that shape the tumor microenvironment (Qian & Pollard, 2010), we selected S180 for tumor inoculation and TAM acquisition. We also compared between bone marrow-derived monocytes (BMDMo) and M-CSF-induced BMDMs and found poor efficacy in training (BMDMo), thus determining to start with macrophages.

Our data validated that TAMEMs recapitulated the reparative features of TAMs and exhibited phenotypes beyond the traditional, *in vitro*-primed M1/M2. Gene analysis showed that though TAMEMs could not fully resemble TAMs, they had a similar transcriptome profile and many of those similar genes are related to tissue repair and immune suppression. TAMEMs highly express mitogenic and angiogenic factors (e.g., VEGF, PDGF, and Ang) and immunosuppressive cytokines (e.g., IL-10, CCL17, and TGF-β), comparable to those in TAMs, which are typical features of the latter in promoting tumor development (Liu & Cao, 2015). This effective recapitulation endows TAMEMs with a comprehensively reparative role that outperforms typical M1 or M2 cells. In our results, M2 macrophages delivered to diabetic wounds fail to exhibit immunosuppressive or reparative functions, though they exert a higher degree of these functions than do their M1 or M0 counterparts, especially *in vitro*. In comparison, TAMEMs express a higher level of growth factors than do M2 macrophages (e.g., *Vegfa*, *Pdgfb*, and *Ang*) and a moderate level of pro-inflammatory genes lower than in M1 but higher than in M2 (e.g., *Cd86*, *Cd80*, and *Ccr2*). Also, TAMEMs induce M2 polarization and resolve inflammation in LPS-challenged fibroblasts, both in a higher capacity than the M2 group. It could be explained that M2 macrophages defined *in vitro* are still different from the diverse macrophage populations involved in tissue repair *in vivo*, which are shaped by myriad factors, undergo a context-dependent phenotypic transition, and co-express M1- and M2a-associated markers in a specific tissue (Novak & Koh, 2013). Further, the higher viability (*in vitro* culture) and longer survival of TAMEMs (after *in vivo* transplantation) than that of M0/M1/M2 macrophages could contribute to their superior effect (Li *et al*, 2016), in addition to their stability in the wound niche. The relatively low plasticity of TAMEMs might be a phenomenon of immune tolerance. Exposure to microbial components, including LPS, can induce macrophage-mediated immune tolerance, resulting in epigenetic changes (Locati *et al*, 2020). Components of TAM-CM could probably trigger epigenetic changes in TAMEMs leading to such tolerance, but more evidence is required in future investigations.

For the translational potential of TAMEMs, we considered about two essential technical aspects. First, although there was no sign of higher expression of tumorigenic genes (e.g., *Raf1* and *Araf*, etc) in TAMEMs and these cells were barely detectable 7-day post-transplantation, their contact with TAM-CM was still a concern for translation. Tumorigenic risk is an important clinical concern given the cases of squamous cell carcinoma development in chronic wounds. Hence, to replace TAM-CM and therefore avoid using any tumor-derived components, we profiled its composition and reconstituted a nine-factor cocktail, which generated TAMEMs$^{C\text{-}m/h}$ that effectively mirrored the functions of TAMEMs. Importantly, TAMEMs$^{C\text{-}h}$ were trained from human peripheral monocyte cells, which verified the possibility of engineering human autologous cells with this approach for future translation. Our finding that TAMEMs$^{C\text{-}h}$ effectively promoted diabetic wound healing in mice

© 2022 The Authors

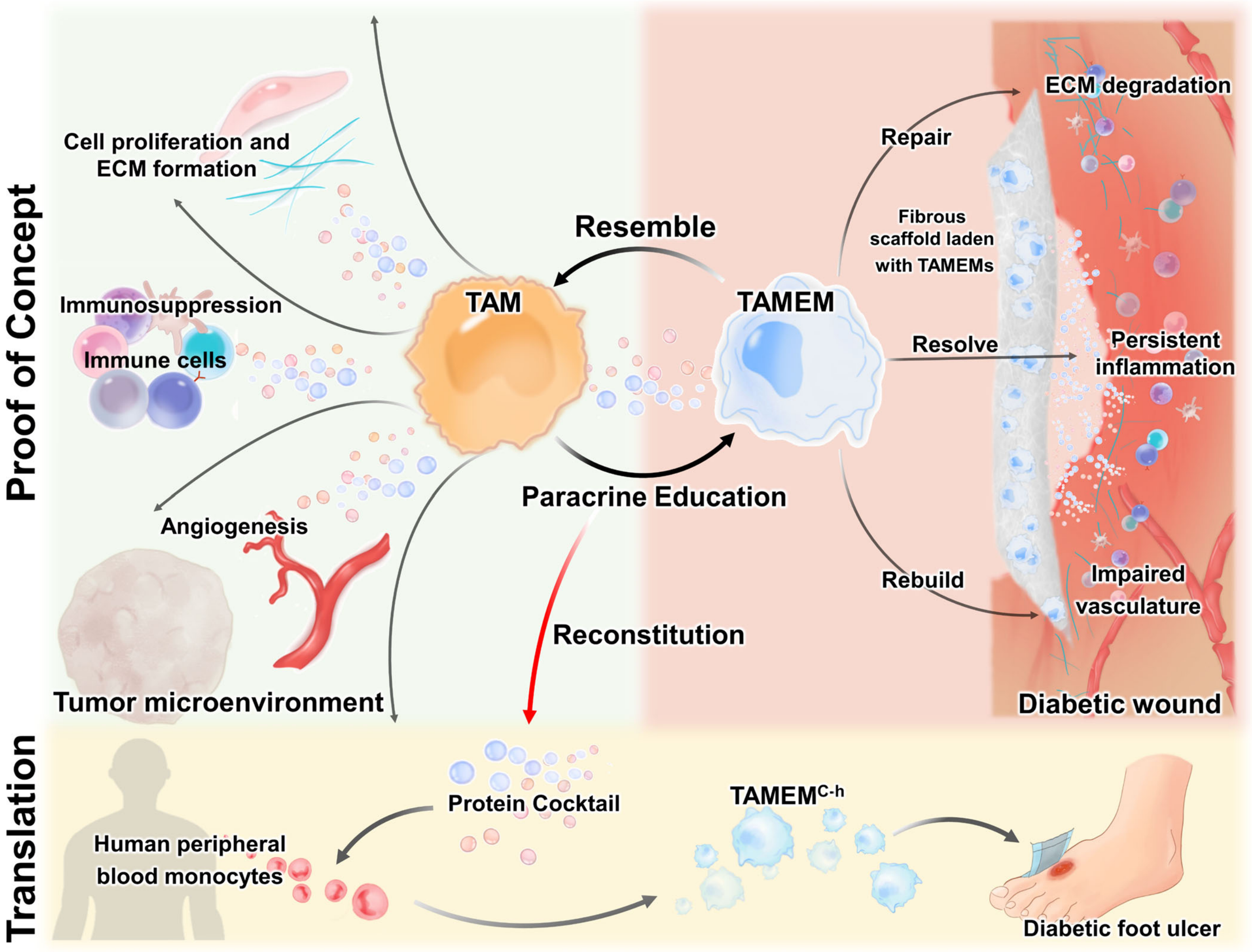

Figure 8. Schematic illustration of translating tumor-associated macrophages (TAMs') functions for diabetic wound healing.

(Up) Preparation of TAMs-educated macrophages (TAMEMs) that recapitulate TAMs' key reparative functions, by culturing normal macrophages with TAMs' conditional medium (TAMs-CM); (Down) Preparation of cytokine-primed human primary macrophages (TAMEMs$^{C\text{-}h}$) resembling the functions of TAMEMs by a nine-factor cocktail reconstituted based on TAMs-CM's composition.

resonated with recent studies that transplanted human immune cells could facilitate the repair of different tissue of mice (Hu *et al*, 2017; Jayme *et al*, 2020), partly thanks to these cytokines involved in chronic inflammatory wound healing, such as VEGF, PDGF, and IL-4, having up to 90% homology between mice and humans (data from MGI, the international database resource for the laboratory mouse). However, our further attempt to replace live cells with the cocktail failed to achieve regenerative outcomes, possibly due to the easy diffusion or degradation of the recombinant proteins exposed to diabetic wounds (Gainza *et al*, 2015). Yet, identifying the cocktail already provided key information to replace tumor-derived components and pursue higher safety. Second, we employed a biomaterials scaffold to deliver TAMEMs (including TAMEMs$^{C\text{-}h}$), providing a three-dimensional biomatrix niche for cell settling and initial physical protection. It is made of gelatin, an FDA-approved biomaterial that is well-known to be biodegradable. We fabricated it into ECM-mimicking fibrous meshes, which showed no adverse effect on macrophage viability or polarization. As an open platform, the composition or type of this scaffold can be customized when TAMEMs are applied for different therapeutic purposes.

Future research may be directed in three ways. The first direction is toward clinical translation. A long-term safety assessment focusing on the immunocytes landscape around the wound is of high significance, and applying human-derived TAMEMs$^{C\text{-}h}$ in more clinically relevant models is desirable. Notably, low cell engraftment is a long-standing mystery for adoptive cell transfer, as observed in the transplantation of stem cells for various therapeutic purposes (Toma *et al*, 2002; Wysoczynski *et al*, 2018); thus, understanding post-transfer cell fate and calculating doses are crucial for translation. Second, TAMs are known for their phenotypic heterogeneity (Qian & Pollard, 2010); more detailed phenotyping of TAMEMs can reveal the correlation between their heterogeneity and their healing

© 2022 The Authors

functions—and more specific functions of different cell clusters. Such information may inspire the development of new reparative macrophages for diseases in various tissue/organs. Third and reversely, the findings in the wound healing model provide valuable information for understanding the basic biology of TAMs in tumor development. For instance, the similarity and differences between TAMs and TAMEMs, as well as the roles of the nine factors, warrant further investigations that may identify new mechanisms underlying TAM's development and function during tumor progression.

Materials and Methods

Reagents

Gelatin (porcine skin, type-A powder), paraformaldehyde, and streptozotocin (STZ) were purchased from Sigma-Aldrich (USA). Collagenase type IV, Collagenase type II, and DNase I were purchased from Sangon Biotech (China). DAPI, Red Blood Cell Lysis Solution, and Citrate Antigen Retrieval solution were provided by Beyotime (China). Mouse CD45 MicroBead Kit and Human CD14 MicroBead Kit were purchased from Miltenyi Biotec (Germany). Proteome Profiler Mouse Cytokine Array Kit and Proteome Profiler Mouse Angiogenesis Array Kit were purchased from R&D Systems (USA). RayBio® L-Series Mouse Antibody Array 308 Membrane Kit was provided by RayBiotech (USA). Corning® Matrigel® Growth Factor Reduced (GFR) Basement Membrane Matrix was provided by Corning (USA).

Primers, antibodies, and recombinant proteins

All primers used for RT–qPCR were synthesized by Sangon Biotech (China), and their sequences were listed in Appendix Table S1. The antibodies and recombinant proteins used in this study are listed in Appendix Tables S2 and S3, respectively.

Cells

Mouse sarcoma cell line (S180), mouse breast cancer cell line (4T1), mouse melanoma cell line (B16-F10), human breast cancer cell line (MCF-7), and human umbilical vein endothelial cell (HUVEC) were purchased from American Type Culture Collection (ATCC). Mouse fibroblast cell lines (L929) were purchased from the Cell Bank of the Chinese academy of sciences. Human primary Peripheral Blood Mononuclear Cells (PBMC) were purchased from StemExpress.

Animals

Female and male C57BL/6J mice (6–8 weeks old), BALB/c mice, and BALB/c Nude mice (6–8 weeks old) were purchased from Vital River Laboratory Animal Technology Co. Ltd (China). C57BL/6J EGFP^{+} mice and BKS-Lepr^{em2Cd479}/Gpt mice (db/db mice; 6–8 weeks old) were purchased from the model animal Research Centre of Nanjing University (China). All animals were housed in a specific-pathogen-free (SPF) animal facility and were treated with controlled light (12-h light/dark cycles), temperature and humidity, and standard chow diet and water available. The experimental animal procedures were reviewed and approved by the University of Macau Animal Research Ethics Committee (approval no. UMARE-037-2017 and UMARE-038-2017) and the Animal Care and Use Committee of Nanjing University (approval no. IACUC-2006003) and were conformed to the Guidelines for the Care and Use of Laboratory Animals published by the National Institutes of Health, USA.

Isolation of tumor-associated macrophages (TAMs) and preparation of TAMs conditional medium (TAMs-CM)

To generate the heterotopic tumor model, mouse sarcoma cell line S180 cells, B16-F10 cells, 4 T1 cells, and MCF-7 cells (1×10^6) were injected subcutaneously into the left armpits of female C57BL/6J mice, female BALB/c mice, and BALB/c Nude mice (6 and 8 weeks of age), respectively. Mice bearing implanted tumors were sacrificed when the sizes of the implanted tumors reached about 1 cm. The tumors were removed, cut into pieces on ice, and immediately transferred into lysis buffer (0.2% collagenase type IV (Sangon Biotech), 0.1% DNase I (Sangon Biotech)) in 1 × HBSS. The sample was digested on a shaker at 90 rpm for 45 min at 37°C. The resulting suspension was filtered through a 70 μm filter, suspended in red blood cell lysis buffer (Beyotime), and incubated on ice for 5 min after washed with ice-cold phosphate-buffered saline pH 7.4 (PBS) and counting. Single-cell suspensions were incubated with 10 μl anti-CD45 magnetic microbeads per 10^7 cells for 20 min at 4–8°C, washed by MACS buffer, and sorted for CD45^{+} leukocytes according to the manufacturer's instructions (Miltenyi Biotec).

For purifying of TAMs, CD45^{+} leukocytes were blocked with 1% bovine serum albumin (BSA) and incubated with the fluorescence-conjugated monoclonal antibodies specific for the following cell surface markers in the dark for 30 min at 4°C: CD45, CD11b, Ly6G, Ly6C, MHC II (BioLegend), and SiglecF (BD Biosciences), followed by flow-sorted on a FACsAria cytometer (BD Biosciences). Gating strategies are shown in Appendix Fig S1A. A part of the resulting TAMs from S180 tumor were prepared for single-cell RNA sequencing, and the remainder were plated in a 75 cm^2 flask containing 30 ml RPMI-1640 media supplemented with 10% FBS and 1% penicillin/streptomycin and then cultured in incubator with 5% CO_2 at 37°C for 15 days. Culture supernatants (TAMs-CM) were collected by collecting the supernatant and centrifuging at 12,000 rpm for 10 min to remove particles and cell debris, subsequently filter-sterilizing through a 0.22 μm Millex-GP syringe filter (Millipore), and then storing at −80°C until further use.

Generation of tumor-associated macrophages-educated macrophages (TAMEMs) and collection of conditional medium from TAMEMs, M2, M1, and M0

Bone marrow-derived macrophages (BMDMs) were obtained and cultured as described previously (Toda *et al*, 2021). Briefly, bone marrow was flushed from mice femurs with ice-cold PBS containing 1% penicillin–streptomycin. After filtering using cell strainers of 70 μm and lysing using red blood cell lysis buffer (Beyotime), BMDMs were differentiated in RPMI-1640 containing 10% fetal bovine serum (FBS), 1% penicillin/streptomycin, and 20 ng/ml recombinant murine M-CSF (Peprotech) for 7 days.

For TAMEMs generation, differentiated BMDMs (M0) were replated and incubated with complete culture medium containing 20% TAMs-CM for 48 h. As control groups, M0 were stimulated for 48 h with: (i) 100 ng/ml LPS and 40 ng/ml IFN-γ for M1 activation

© 2022 The Authors

or (ii) 40 ng/ml IL-4 and 20 ng/ml IL-13 for M2 activation. Next, differentiated TAMEMs, M1, M2, and M0 were washed twice, gently but adequately with PBS, and the old media containing the stimuli were removed. Then, depending on different experimental needs, the cells were (A) directly processed for cellular analysis; (B) cultured in the RPMI-1640 complete medium for another 48 h for collection of supernatant; or (C) cultured in the RPMI-1640 complete medium for another 96 h for observing cell morphology or other purposes.

For Choice B, all culture supernatants (termed conditional medium) were collected after 48 h of culture, followed by centrifuge at 12,000 rpm for 10 min to remove particles and cell debris, and subsequently filter-sterilized through a 0.22 μm Millex-GP syringe filter.

Profiling of soluble factors by mouse cytokine array kit and mouse angiogenesis array kit

To investigate the paracrine functions of TAMEMs, the soluble components of M0-CM, M1-CM, M2-CM, and TAMEMs-CM were analyzed by membrane-based antibody assay kit for 78 kinds of different soluble factors (R&D, USA; including Proteome Profiler Mouse Cytokine Array kit and Mouse Angiogenesis Array Kit) according to the manufacturers' protocols.

Cytokine absolute concentration determination with Luminex® Assays system

To quantify absolute concentrations of selected 22 cytokines, the soluble components of M0-CM, M1-CM, M2-CM, and TAMEMs-CM were analyzed by Luminex® Assay (R&D, USA) according to the manufacturers' protocols.

RNA isolation and quantitative real-time qPCR

Total RNA from cells or tissues was extracted with Trizol (Invitrogen) using standard procedures and then reverse transcribed into cDNA using GoScript™ Reverse Transcriptase according to the manufacturer's instructions (Promega). Quantitative real-time PCR analysis was performed using a GoTaq® qPCR Master Mix Kit (Promega) in an ABI 7300 Fast Real-time PCR System (Applied Biosystems, FosterCity, CA). The relative mRNA expression level of each gene was normalized with the β-actin in the same sample. Primer sequences are listed in Appendix Table S1.

Treatment of M0, fibroblast (L929), and mouse lymph node endothelial cells (SVEC4-10) with TAMEMs-conditioned medium

For the macrophage paracrine stimulation assay, M0, fibroblast (L929), and SVEC4-10 were separately incubated with normal medium, M0-conditioned medium (M0-CM), M1-conditioned medium (M1-CM), M2-conditioned medium (M2-CM), and TAMEMs-conditioned medium (TAMEMs-CM) prepared as described previously. The medium in all dishes was refreshed daily, and cells were cultured at 37°C. Cells were subjected to subsequent analyses.

RNA sequencing

Total RNA was extracted as described in supplementary methods, and quality was determined using an Agilent 2100 Bioanalyzer. RNA-sequencing libraries were constructed using a Seq-Star™ Rapid RNASeq Kit (Illumina, California, USA). The cDNA in each sample was sequenced on the Illumina Hiseq4000 (Shanghai Meiji Biomedical Technology Inc., Shanghai, China). For single-cell RNA sequencing (scRNA-seq), TAMEMs, M2, M1, and TAMs that sorted above were resuspended in HBSS containing 0.04% BSA buffer. Single-cell libraries were generated via the Chromium Controller and the Single Cell 30 Reagent Kit v3 (10× Genomics, Pleasanton, CA) according to the manufacturer's instructions and then sequenced on an Illumina HiSeq4000 platform (LC-BIO Biotech ltd, Hangzhou, China). After obtaining the digital gene expression data matrix, we used Seurat3.0 (https://satijalab.org/seurat/) for dimension reduction, unbiased clustering, and cell types identification. R Bioconductor package Monocle was used in the pseudotime analysis.

Electrospinning fabrication of gelatin fibers

Gelatin (Sigma-Aldrich, 80 mg) was dissolved in trifluoroethanol (TFE, J&K®, China; 1 ml). The solution was filled into a syringe and kept in a syringe pump attached with the electrospinning instrument (Tongli Weina, China). The fibers were collected with an aluminum foil collector plate (7 × 7 cm). The distance between the syringe and the collector was set at 15 cm, with a 15 kV voltage and flow rate of 1 ml/h. The obtained fibers were crosslinked into scaffolds by using the glutaraldehyde vapor (0.25%, w/v) for 30 min at room temperature, followed by washing with 1 M glycine aqueous solution to block the unreacted glutaraldehyde.

Giemsa stain

To observe the cell morphology of TAMs, TAMEMs, and other cells, Giemsa staining was performed according to the manufacturers' protocols (Abcam, Giemsa Stain Kit). Briefly, spread cells on glass slides and slides were then air-dried for 10 min prior to staining. Immerse slides in Giemsa staining solution for 10–15 min. After removing the excess staining, soak the slides in PBS buffer until no excess staining solution flows out. Before taking pictures with the microscope, wipe the back of the slide in a vertical position and set it to dry and xylene clear for 10 min.

Cellular proliferation assay

To examine the survival curve of TAMEMs and other control cells, CCK-8 assay was performed. Briefly, after removing the stimuli-containing medium, the cells were washed with PBS twice, reseeded in 96-well plates at an initial density of 4,000 cells/well, and cultured in the RPMI-1640 complete medium for 1, 3, 5, 7, 9, and 11 days. To each well, 10 μl of CCK-8 reagent was added and incubated for 1 h at 37°C according to the manufacturer's protocol. The absorption was measured at 450 nm, and the reference wavelength was 600 nm. Cell proliferation rates are reported as a percentage of the absorbance measured in the test samples relative to the control experiment without treatment.

Wound healing assay

For the migration assay, the wound healing assay was done. The cell gap is created by using the 2 Well Culture-Inserts purchased

© 2022 The Authors

from ibidi (Wisconsin, USA). Briefly, choose a suitable Culture-Insert in 24-well plates. Seed and culture L929 cells until they form an optically confluent monolayer. Let the cells grow for approximately 24 h and then remove the Culture-Insert to create the gap. Subsequently, the media were replaced with normal medium, M0-CM, M1-CM, M2-CM, or TAMEMs-CM. Photographs were taken at 0, 4, and 8 h in an inverted microscope; then, the wound area was analyzed by ImageJ software.

Tube formation assay of SVEC4-10

To examine the ability of the angiogenesis of TAMEMs, a tube formation assay was performed using the μ-Slide Angiogenesis purchased from ibidi. The SVEC4-10 cells were seeded on the Matrigel (Corning) and treated with normal medium, M0-CM, M1-CM, M2-CM, TAMEMs-CM, or VEGF. After incubation at 37°C for 4 h, the cells were stained with Calcein AM, followed by imaging of the tube formation.

Western blotting

Protein extractions and western blotting were performed to test the function of TAMEMs *in vitro*. Proteins were separated by 10–15% SDS–PAGE and transferred to a polyvinylidene fluoride membrane. The blots were blocked with blocking buffer (5% BSA or skim milk in PBS) and incubated with diluted primary antibodies- α-SMA, collagen type I (Col I; Abcam), IκBα, and Phospho-IκBα (Cell Signaling) at 4°C with gentle shaking overnight. After three times washing with TBST (50 mM Tris. pH 7.4, 150 mM NaCl, 0.05% Tween 20), the blots were incubated with HRP-conjugated anti-rabbit, or anti-mouse (Cell Signaling) for 1 h at room temperature. After washing, bands were visualized with fluorography using an enhanced chemiluminescence system.

Diabetic wound healing models in mice and TAMEMs transplantation

For Type 1 Diabetes (T1D) models, male C57BL/6J mice (6 and 8 weeks of age; Vital River Laboratory Animal Technology Co. Ltd., Beijing China) were intraperitoneally injected with streptozotocin (STZ; 50 mg/kg, Sigma-Aldrich), for 5 consecutive days after a 12 h fast. The mice were considered diabetic when their blood glucose levels consistently exceeded 14 mmol/l. Two weeks after the initiation of the STZ treatment, a full-thickness excisional splinted wound (6 mm in diameter) was created on the dorsal section with a punch (Tan & Wahli, 2013). For Type 2 Diabetes (T2D) models, db/db mice were selected and wounds were created as described above. Diabetic mice were randomly divided into five groups: group I: Blank control; group II: TAMEMs treatment; group III: M2 treatment; group IV: M1 treatment; group V: M0 treatment. TAMEMs, M2, M1, and M0 were seeded (in PBS) onto gelatin electrospinning fibers and transplanted (3×10^5 cells per wound) onto 6-mm full-thickness wounds on the dorsum of experimental group mice, respectively (Hu *et al*, 2017). Group I, which received blank gelatin electrospinning fibers without cells, served as a sham control group. Wounds were also generated in the skin of healthy C57BL/6J mice and treatment with blank gelatin electrospinning fibers without cells as a normal mice control. Transparent dressings (TegadermTM Film, 3M) were applied on all wounds and changed every other day. Digital images of the wound were captured with a camera (Nikon) at each time point (days 0, 3, 7, 11, 14, and 21) and analyzed using ImageJ software.

Vessel perfusion assay

For the analysis of blood vessel regression during the resolution phase of wound repair, DyLight® 649-labeled tomato lectin (VECTOR) was injected into diabetic wounded mice by intravenous (5 mg/kg). Ten minutes later, wound site tissues were fixed in 4% paraformaldehyde (PFA, Sigma-Aldrich) for 24 h at room temperature in the dark. Two-photon imaging with z-compensation was performed to detect the blood vessel in the wound site. Multiphoton images were generated on a Zeiss fluorescence microscope (LSM 980 NLO, ZEISS). Digital images of the wound blood vessels were analyzed by ImageJ software, and the orientational distribution of blood vessels was quantified by Orientational J (a plugin of ImageJ).

Direct application of recombinant protein cocktail in diabetic wounds

A full-thickness excisional splinted wound (6 mm in diameter) was created on the dorsal section with a punch in STZ-induced T1D mice. Diabetic mice were randomly divided into three groups: group I: Blank control, which received blank gelatin electrospinning fibers without cells; group II: TAMEMs treatment, which received gelatin electrospinning fibers with 3×10^5 TAMEMs cells; group III: Cocktail treatment, which received blank gelatin electrospinning fibers without cells, but infiltrated with recombinant protein cocktails (use the same concentration as inducing TAMEMs$^{C\text{-}m}$). Digital images of the wound were captured with a camera (Nikon) at each time point (days 0, 7, 14, and 21) and analyzed using ImageJ software.

Histology and tissue analysis

For the histological analysis, tissues were placed in 4% PFA for 24 h at room temperature and then embedded in paraffin. Wound tissues were sectioned at 5 μm thickness and stained with a Haematoxylin-Eosin Staining kit (Jiancheng Bioengineering Institute). For collagen staining, Masson's Staining Kit (Solarbio Science) was used according to the manufacturer's instructions.

Immunofluorescence staining and quantification

For immunofluorescence staining *in vitro*, cells were fixed for 20 min and permeabilized for 15 min at room temperature with 4% PFA and 0.3% Triton X-100 in PBS, respectively. Then, cells were blocked with PBS containing 5% BSA at room temperature for 1 h, followed by incubation with primary antibody overnight at 4°C. Then, cells were washed three times with PBS and incubated for 2 h at room temperature with anti-rabbit or anti-mouse secondary antibodies. For immunofluorescence staining of wound tissues, paraffin sections were deparaffinized and rehydrated and then subjected to a heat-mediated antigen retrieval step using citrate antigen retrieval solution (pH 6.0; Beyotime). The slides were blocked with 5% BSA in PBS for 1 h at room temperature and then incubated with primary antibody at 4°C overnight. After three washes with PBS, slides were incubated for 1 h at

© 2022 The Authors

room temperature with corresponding secondary antibodies (Life Technologies). The slides were mounted with coverslips after counterstained with DAPI for 5 min and then examined with a Zeiss fluorescence microscope. The expression of specific proteins be semiquantitatively analyzed by detecting the mean gray value of a certain fluorescence in the immunofluorescence photographs. The gray value of each pixel represents the fluorescence intensity of the point, and the mean gray value of a certain fluorescence channel on the immunofluorescence photograph can be quantified using ImageJ.

Flow cytometry

For flow cytometry analysis *in vitro*, cells were digested to single-cell suspension, followed by the incubation with 1% BSA containing the specific fluorescence-conjugated monoclonal antibodies for 30 min on ice. For the flow cytometry analysis of wound tissue, wounds were excised and cut into pieces on ice and then gently dissociated in lysis buffer (2.5 mg/ml collagenase type II (Sangon Biotech), 2.5 mg/ml collagenase type IV (Sangon Biotech), and 0.5 mg/ml DNase I (Sangon Biotech)) in 1 × HBSS. The resulting cells were passed through a 70 μm cell strainer and suspended in red blood cell lysis buffer. Thereafter, cells were used for specific fluorescence-conjugated monoclonal antibodies staining, followed by flow cytometry assessment (Attune® NxT Flow Cytometer).

Quantitative real-time qPCR for cells transplanted in wounds

Following the application of macrophages to excised skin wounds, tissue was harvested from the delivery site 3 days after surgery, minced, and digested using collagenase type II and collagenase type IV for 1 h at 37°C. To stop the reaction, cells were centrifuged and resuspended as a single-cell suspension in buffer (PBS with 1% BSA). GFP^+ cells were sorted as single cells using a FACsAria cytometer (BD Biosciences). Cultured macrophages were sorted as a control population. Total RNA from transplanted cells was isolated using the RNeasy 96 Kit (QIAGEN) following the manufacturer's instructions. Then, reverse transcribed into cDNA using GoScript™ Reverse Transcriptase according to the manufacturer's instructions (Promega). Quantitative real-time PCR analysis was performed using a GoTaq® qPCR Master Mix Kit (Promega) in an ABI 7300 Fast Real-time PCR System (Applied Biosystems, FosterCity, CA). The relative mRNA expression level of each gene was normalized with the β-actin in the same sample.

SEM of TAMEMs located on gelatin electrospinning fibers

3×10^4 TAMEMs were seeded on gelatin electrospinning fibers (6 × 6 mm) overnight and then fixed in 2.5% glutaraldehyde solution for 24 h in 4°C. After rinsing with buffer, cells were dehydrated with graded alcohol (30, 50, 70, 90, and 100% for 10 min each), followed by freeze-drying. Subsequently, the samples underwent fixation on SEM stubs and gold sputter coating. Then, images of TAMEMs on electrospinning fiber were captured using a field emission SEM microscope (LEO1530VP) in a high-vacuum environment.

Biosafety assessment of gelatin electrospinning fibers

M0 (BMDMs) were obtained by culturing bone marrow-derived cells with M-CSF for 7 days, and TAMEMs were obtained by culturing M0 with TAMs-CM for another 2 days. After that, M0 and TAMEMs are divided into two groups, respectively: one group seeded on the culture plate and the other on the electrospun scaffolds, followed by culture in the same RPMI-1640 complete medium for 2 days. At the end of these 2 days, the change folds of M1 and M2 markers in both scaffold-cultured and plate-cultured cells were all compared with plate-cultured M0, as analyzed by qPCR, using *t*-test.

Antibody protein array of TAMs-CM

An antibody array (RayBio® Biotin Label-based L-Series Mouse Antibody Array 308 Membrane Kit, RayBiotech) was performed to detect 308 different mouse target proteins of TAMs-CM, including cytokines, chemokines, adipokines, growth factors, proteases, soluble receptors, soluble adhesion molecules, and other proteins (Table EV1). Detailed experimental procedures and protein array list see https://www.raybiotech.com/files/manual/Antibody-Array/AAM-BLM-1A.pdf. The result was analyzed with RayBio® Antibody Array Analysis Tool using a normalization according to positive control densities after background subtraction.

Preparation of the recombinant protein cocktail

The above proteins in TAMs-CM identified by antibodies array were divided into five categories according to their functions. In each category, two proteins (except only one for colony-stimulating factor) were selected, which had macrophage-inductive functions and high abundance. These nine factors were quantified using ELISA: Osteopontin (113.215 ng/ml), IL-31 (9.102 ng/ml), IL-10 (7.372 ng/ml), TGF-beta 2 (5.211 ng/ml), M-CSF (10.157 ng/ml), MIP-2 (42.513 ng/ml), CCL8 (21.894 ng/ml), VEGF-B (11.799 ng/ml), and bFGF (3.522 ng/ml). Based on these concentrations, we reconstituted the cocktail in the RPMI-1640 to mimic TAMs-CM—with recombinant proteins of mouse and human origins, for training BMDMs into $TAMEMs^{C\text{-}m}$ and human $CD14^+$ monocytes into $TAMEMs^{C\text{-}h}$, respectively.

Generation of cocktail-educated, TAMEMs-mimicking mouse macrophages ($TAMEMs^{C\text{-}m}$) and human macrophages ($TAMEMs^{C\text{-}h}$), and collection of conditional medium from $TAMEMs^{C\text{-}m}$

For $TAMEMs^{C\text{-}m}$ generation, BMDMs (M0) were cultured in a complete RPM1-1640 medium containing 20% murine recombinant protein cocktail for 48 h. For the generation of 18 other control macrophages, BMDMs were cultured in a complete RPM1-1640 medium containing in: (i) nine "minus" cocktails that each was ripped of one factor and (ii) nine "added" medium that each was supplemented with only one factor for 48 h. For $TAMEMs^{C\text{-}h}$ generation, frozen human primary peripheral blood mononuclear cells (PBMC; StemExpress (USA)) were incubated with anti-CD14 magnetic microbeads (Miltenyi Biotec) according to the manufacturer's instructions for purifying the monocytes. Then, these monocytes were differentiated into human peripheral macrophages (M0) after culturing in a complete RPMI-1640 medium containing 20 ng/ml recombinant human M-CSF (Peprotech) for 7 days. Finally, the differentiated human peripheral macrophages were induced into $TAMEMs^{C\text{-}h}$ after replating and incubating with a complete RPMI-1640 medium containing 20% human recombinant protein cocktail for 48 h. As control

© 2022 The Authors

The paper explained

Problem

Diabetes causes chronic nonhealing wounds that affect 60 million people worldwide. Existing approaches for wound healing, which may be effective for normal wounds, have gained limited success under diabetic circumstances. A key biological obstacle is with the local immune cells: the early macrophages that initiate healing fail to transit into pro-repair phenotypes as they do in normal wounds, leading to multifacet cell dysfunction. It remains unknown which type of immune cells is optimal for delivery to the wound milieu for rectifying the local immune niche.

Results

We assume that tumor-associated macrophages (TAMs), which play essential roles in shaping the tumor microenvironment through paracrine secretion of abundant growth factors and cytokines, could be an unconventional inspiration. We generate TAMs-educated macrophages (TAMEMs) to recapitulate TAMs' reparative functions, exerting TAMs' key reparative activities in murine diabetic wounds—including modulating inflammation, stimulating angiogenesis, and promoting proliferation. For translational considerations, we further identify a nine-factor cocktail to train human primary monocytes as substitutes of TAMEMs, avoiding use of any tumor-derived components, which efficiently promote diabetic wound healing in immunodeficient mice.

Impact

Our data demonstrate the possibility to recapitulate TAMs' comprehensive reparative functions in normal macrophages to promote diabetic wound healing, thereby proposing a new type of therapeutic cells for regenerative medicine.

groups, differentiated human peripheral macrophages were stimulated for 48 h with 100 ng/ml LPS and 40 ng/ml IFN-γ (human) for M1 activation, and 40 ng/ml IL-4 (human) and 20 ng/ml IL-13 (human) for M2 activation, respectively. For conditional medium collection, differentiated TAMEMs$^{C\text{-}m}$, M1, M2, and M0 were washed twice with PBS after removing the medium containing stimulants and then cultured in the RPMI-1640 complete medium for another 48 h. All the cultural supernatants (termed conditional medium) were collected after 48 h of culture, followed by centrifuge at 12,000 rpm for 10 min to remove particles and cell debris, and subsequently filter-sterilized through a 0.22 μm Millex-GP syringe filter.

Statistics

The results are expressed as mean ± standard deviation (SD). All data were statistically analyzed in Prism software (GraphPad). Differences between two groups were analyzed using a two-tailed unpaired *t*-test, and differences between multiple groups were compared using one-way analysis of variance (ANOVA) with Dunnett's tests. Two-way ANOVA with Dunnett's multiple comparisons test and two-way ANOVA with Sidak's multiple comparisons test was also used in this study. A value of $P \leq 0.05$ was considered significant; "ns" stands for "not significant."

Data availability

The raw sequencing data of RNA-seq have been deposited to the Sequence Read Archive database and registered with BioProject as BioProject ID PRJNA898592 https://www.ncbi.nlm.nih.gov/bioproject/PRJNA898592; data available from SRP406418.

Expanded View for this article is available online.

Acknowledgements

This study was funded by The Science and Technology Development Fund, Macau SAR (File no. 0097/2019/A2 and 0001/2021/AKP), National Natural Science Foundation of China (32022088, 32230056, and 31961160701), Shenzhen-Hong Kong-Macau Science and Technology Program Category C (SGDX20201103092803014) of the Shenzhen Science and Technology Innovation Commission, and the University of Macau (MYRG2020-00084-ICMS). The authors thank Mr Guangpan (Carlos) Zhang for preliminary tests, Ms Huiqun Zhou for technical guidance, and members of the Macau Society for Stem Cell Research (MSSCR) for inspiring discussion.

Author contributions

Ruoyu Mu: Conceptualization; data curation; investigation; visualization; methodology; writing – original draft; writing – review and editing. **Zhe Zhang:** Conceptualization; investigation. **Congwei Han:** Data curation; investigation. **Yiming Niu:** Data curation; formal analysis. **Zhen Xing:** Data curation. **Zhencheng Liao:** Visualization. **Jinzhi Xu:** Investigation. **Ningyi Shao:** Supervision; methodology. **Guokai Chen:** Supervision; methodology. **Junfeng Zhang:** Resources; supervision; methodology. **Lei Dong:** Resources; data curation; supervision; writing – original draft; project administration. **Chunming Wang:** Conceptualization; supervision; funding acquisition; writing – original draft; project administration; writing – review and editing.

Disclosure and competing interests statement

The authors declare that they have no conflict of interest.

References

Alciato F, Sainaghi PP, Sola D, Castello L, Avanzi GC (2010) TNF-alpha, IL-6, and IL-1 expression is inhibited by GAS6 in monocytes/macrophages. *J Leukoc Biol* 87: 869 – 875

Babazadeh S, Nassiri SM, Siavashi V, Sahlabadi M, Hajinasrollah M, Zamani-Ahmadmahmudi M (2021) Macrophage polarization by MSC-derived CXCL12 determines tumor growth. *Cell Mol Biol Lett* 26: 30

Boniakowski AE, Kimball AS, Jacobs BN, Kunkel SL, Gallagher KA (2017) Macrophage-mediated inflammation in normal and diabetic wound healing. *J Immunol* 199: 17 – 24

Boniakowski AE, Kimball AS, Joshi A, Schaller M, Davis FM, denDekker A, Obi AT, Moore BB, Kunkel SL, Gallagher KA (2018) Murine macrophage chemokine receptor CCR2 plays a crucial role in macrophage recruitment and regulated inflammation in wound healing. *Eur J Immunol* 48: 1445 – 1455

Bosurgi L, Cao YG, Cabeza-Cabrerizo M, Tucci A, Hughes LD, Kong Y, Weinstein JS, Licona-Limon P, Schmid ET, Pelorosso F *et al* (2017) Macrophage function in tissue repair and remodeling requires IL-4 or IL-13 with apoptotic cells. *Science* 356: 1072 – 1076

Brem H, Tomic-Canic M (2007) Cellular and molecular basis of wound healing in diabetes. *J Clin Investig* 117: 1219 – 1222

Burgess M, Wicks K, Gardasevic M, Mace KA (2019) Cx3CR1 expression identifies distinct macrophage populations that contribute differentially to inflammation and repair. *Immunohorizons* 3: 262 – 273

© 2022 The Authors

Cassetta L, Pollard JW (2020) Tumor-associated macrophages. *Curr Biol* 30: R246 – R248

Cavanagh PR, Lipsky BA, Bradbury AW, Botek G (2005) Treatment for diabetic foot ulcers. *Lancet* 366: 1725 – 1735

Chen P, Huang Y, Bong R, Ding Y, Song N, Wang X, Song X, Luo Y (2011) Tumor-associated macrophages promote angiogenesis and melanoma growth via adrenomedullin in a paracrine and autocrine manner. *Clin Cancer Res* 17: 7230 – 7239

Chu HX, Arumugam TV, Gelderblom M, Magnus T, Drummond GR, Sobey CG (2014) Role of CCR2 in inflammatory conditions of the central nervous system. *J Cereb Blood Flow Metab* 34: 1425 – 1429

Danon D, Kowatch MA, Roth GS (1989) Promotion of wound repair in old mice by local injection of macrophages. *Proc Natl Acad Sci USA* 86: 2018 – 2020

Danon D, Madjar J, Edinov E, Knyszynski A, Brill S, Diamantshtein L, Shinar E (1997) Treatment of human ulcers by application of macrophages prepared from a blood unit. *Exp Gerontol* 32: 633 – 641

Di Mitri D, Mirenda M, Vasilevska J, Calcinotto A, Delaleu N, Revandkar A, Gil V, Boysen G, Losa M, Mosole S *et al* (2019) Re-education of tumor-associated macrophages by CXCR2 blockade drives senescence and tumor inhibition in advanced prostate cancer. *Cell Rep* 28: 2156 – 2168

Dürr C, Pfeifer D, Claus R, Schmitt-Graeff A, Gerlach UV, Graeser R, Krüger S, Gerbitz A, Negrin RS, Finke J *et al* (2010) CXCL12 mediates immunosuppression in the lymphoma microenvironment after allogeneic transplantation of hematopoietic cells. *Cancer Res* 70: 10170 – 10181

Dvorak HF (2015) Tumors: wounds that do not heal-redux. *Cancer Immunol Res* 3: 1 – 11

Eming SA, Martin P, Tomic-Canic M (2014) Wound repair and regeneration: mechanisms, signaling, and translation. *Sci Transl Med* 6: 265sr6

Falanga V (2005) Wound healing and its impairment in the diabetic foot. *Lancet* 366: 1736 – 1743

Ferreira DW, Ulecia-Morón C, Alvarado-Vázquez PA, Cunnane K, Moracho-Vilriales C, Grosick RL, Cunha TM, Romero-Sandoval EA (2020) CD163 overexpression using a macrophage-directed gene therapy approach improves wound healing in *ex vivo* and *in vivo* human skin models. *Immunobiology* 225: 151862

Franklin RA, Liao W, Sarkar A, Kim MV, Bivona MR, Liu K, Pamer EG, Li MO (2014) The cellular and molecular origin of tumor-associated macrophages. *Science* 344: 921 – 925

Fujimura T, Kambayashi Y, Fujisawa Y, Hidaka T, Aiba S (2018) Tumor-associated macrophages: therapeutic targets for skin cancer. *Front Oncol* 8: 3

Gainza G, Villullas S, Pedraz JL, Hernandez RM, Igartua M (2015) Advances in drug delivery systems (DDSs) to release growth factors for wound healing and skin regeneration. *Nanomed Nanotechnol Biol Med* 11: 1551 – 1573

Gleissner CA, Shaked I, Little KM, Ley K (2010) CXC chemokine ligand 4 induces a unique transcriptome in monocyte-derived macrophages. *J Immunol* 184: 4810 – 4818

Gurevich DB, Severn CE, Twomey C, Greenhough A, Cash J, Toye AM, Mellor H, Martin P (2018) Live imaging of wound angiogenesis reveals macrophage orchestrated vessel sprouting and regression. *EMBO J* 37: e97786

Han G, Wu Z, Zhao N, Zhou L, Liu F, Niu F, Xu Y, Zhao X (2017) Overexpression of stathmin plays a pivotal role in the metastasis of esophageal squamous cell carcinoma. *Oncotarget* 8: 61742 – 61760

Hocking AM (2012) Mesenchymal stem cell therapy for cutaneous wounds. *Adv Wound Care* 1: 166 – 171

Hu MS, Walmsley GG, Barnes LA, Weiskopf K, Rennert RC, Duscher D, Januszyk M, Maan ZN, Hong WX, Cheung AT *et al* (2017) Delivery of monocyte lineage cells in a biomimetic scaffold enhances tissue repair. *JCI Insight* 2: e96260

Hu J, Zhao Q, Kong LY, Wang J, Yan J, Xia X, Jia Z, Heimberger AB, Li S (2021) Regulation of tumor immune suppression and cancer cell survival by CXCL1/2 elevation in glioblastoma multiforme. *Sci Adv* 7: eabc2511

Hua Y, Bergers G (2019) Tumors vs. chronic wounds: an immune cell's perspective. *Front Immunol* 10: 2178

Im JH, Buzzelli JN, Jones K, Franchini F, Gordon-Weeks A, Markelc B, Chen J, Kim J, Cao Y, Muschel RJ (2020) FGF2 alters macrophage polarization, tumour immunity and growth and can be targeted during radiotherapy. *Nat Commun* 11: 4064

Jayme TS, Leung G, Wang A, Workentine ML, Rajeev S, Shute A, Callejas BE, Mancini N, Beck PL, Panaccione R *et al* (2020) Human interleukin-4-treated regulatory macrophages promote epithelial wound healing and reduce colitis in a mouse model. *Sci Adv* 6: eaba4376

Jetten N, Roumans N, Gijbels MJ, Romano A, Post MJ, De Winther MPJ, Van Der Hulst RRWJ, Xanthoulea S (2014) Wound administration of M2-polarized macrophages does not improve murine cutaneous healing responses. *PLoS One* 9: e102994

Julier Z, Park AJ, Briquez PS, Martino MM (2017) Promoting tissue regeneration by modulating the immune system. *Acta Biomater* 53: 13 – 28

Kasraie S, Niebuhr M, Werfel T (2009) Interleukin (IL)-31 induces pro-inflammatory cytokines in human monocytes and macrophages following stimulation with staphylococcal exotoxins. *Allergy* 65: 712 – 721

Kimball A, Schaller M, Joshi A, Davis FM, Dendekker A, Boniakowski A, Bermick J, Obi A, Moore B, Henke PK *et al* (2018) Ly6C hi blood monocyte/macrophage drive chronic inflammation and impair wound healing in diabetes mellitus. *Arterioscler Thromb Vasc Biol* 38: 1102 – 1114

Lai YS, Wahyuningtyas R, Aui SP, Chang KT (2019) Autocrine VEGF signalling on M2 macrophages regulates PD-L1 expression for immunomodulation of T cells. *J Cell Mol Med* 23: 1257 – 1267

Lamort A-S, Giopanou I, Psallidas I, Stathopoulos GT (2019) Osteopontin as a link between inflammation and cancer: the thorax in the spotlight. *Cell* 8: 815

Li L, Chen X, Wang WE, Zeng C (2016) How to improve the survival of transplanted mesenchymal stem cell in ischemic heart? *Stem Cells Int* 2016: 9682757

Lin EY, Pollard JW (2007) Tumor-associated macrophages press the angiogenic switch in breast cancer. *Cancer Res* 67: 5064 – 5066

Lin EY, Li JF, Gnatovskiy L, Deng Y, Zhu L, Grzesik DA, Qian H, Xue XN, Pollard JW (2006) Macrophages regulate the angiogenic switch in a mouse model of breast cancer. *Cancer Res* 66: 11238 – 11246

Liu Y, Cao X (2015) The origin and function of tumor-associated macrophages. *Cell Mol Immunol* 12: 1 – 4

Liu Q, Yu S, Li A, Xu H, Han X, Wu K (2017) Targeting interlukin-6 to relieve immunosuppression in tumor microenvironment. *Tumour Biol* 39: 1010428317712445

Locati M, Curtale G, Mantovani A (2020) Diversity, mechanisms, and significance of macrophage plasticity. *Annu Rev Pathol* 15: 123 – 147

Ma RY, Black A, Qian BZ (2022) Macrophage diversity in cancer revisited in the era of single-cell omics. *Trends Immunol* 43: 546 – 563

Motz GT, Coukos G (2011) The parallel lives of angiogenesis and immunosuppression: cancer and other tales. *Nat Rev Immunol* 11: 702 – 711

Movahedi K, Laoui D, Gysemans C, Baeten M, Stange G, Van Den Bossche J, Mack M, Pipeleers D, In'T Veld P, De Baetselier P *et al* (2010) Different tumor microenvironments contain functionally distinct subsets of

© 2022 The Authors

macrophages derived from Ly6C(high) monocytes. *Cancer Res* 70: 5728 – 5739

Nepal S, Tiruppathi C, Tsukasaki Y, Farahany J, Mittal M, Rehman J, Prockop DJ, Malik AB (2019) STAT6 induces expression of Gas6 in macrophages to clear apoptotic neutrophils and resolve inflammation. *Proc Natl Acad Sci USA* 116: 16513 – 16518

Novak ML, Koh TJ (2013) Macrophage phenotypes during tissue repair. *J Leukoc Biol* 93: 875 – 881

Okonkwo U, Dipietro L (2017) Diabetes and wound angiogenesis. *Int J Mol Sci* 18: 1419

Perez F, Martinez F, Rodriguez L, Cid B, Zamudio O, Martinez B, Mollinedo M, Rodriguez S, Castañeda M, Garza V (2019) Current therapeutic strategies in diabetic foot ulcers. *Medicina* 55: 714

Poltavets V, Kochetkova M, Pitson SM, Samuel MS (2018) The role of the extracellular matrix and its molecular and cellular regulators in cancer cell plasticity. *Front Oncol* 8: 431

Priceman SJ, Sung JL, Shaposhnik Z, Burton JB, Torres-Collado AX, Moughon DL, Johnson M, Lusis AJ, Cohen DA, Iruela-Arispe ML *et al* (2010) Targeting distinct tumor-infiltrating myeloid cells by inhibiting CSF-1 receptor: combating tumor evasion of antiangiogenic therapy. *Blood* 115: 1461 – 1471

Qian BZ, Pollard JW (2010) Macrophage diversity enhances tumor progression and metastasis. *Cell* 141: 39 – 51

Rantakari P, Patten DA, Valtonen J, Karikoski M, Gerke H, Dawes H, Laurila J, Ohlmeier S, Elima K, Hübscher SG *et al* (2016) Stabilin-1 expression defines a subset of macrophages that mediate tissue homeostasis and prevent fibrosis in chronic liver injury. *Proc Natl Acad Sci USA* 113: 9298 – 9303

Sawaya AP, Stone RC, Brooks SR, Pastar I, Jozic I, Hasneen K, O'Neill K, Mehdizadeh S, Head CR, Strbo N *et al* (2020) Deregulated immune cell recruitment orchestrated by FOXM1 impairs human diabetic wound healing. *Nat Commun* 11: 4678

Scheuerer B, Ernst M, Dürrbaum-Landmann I, Fleischer J, Grage-Griebenow E, Brandt E, Flad HD, Petersen F (2000) The CXC-chemokine platelet factor 4 promotes monocyte survival and induces monocyte differentiation into macrophages. *Blood* 95: 1158 – 1166

Sica A, Saccani A, Bottazzi B, Polentarutti N, Vecchi A, van Damme J, Mantovani A (2000) Autocrine production of IL-10 mediates defective IL-12 production and NF-kappa B activation in tumor-associated macrophages. *J Immunol* 164: 762 – 767

Solinas G, Germano G, Mantovani A, Allavena P (2009) Tumor-associated macrophages (TAM) as major players of the cancer-related inflammation. *J Leukoc Biol* 86: 1065 – 1073

Tan NS, Wahli W (2013) Studying wound repair in the mouse. *Curr Protoc Mouse Biol* 3: 171 – 185

Toda G, Yamauchi T, Kadowaki T, Ueki K (2021) Preparation and culture of bone marrow-derived macrophages from mice for functional analysis. *STAR Protoc* 2: 100246

Toma C, Pittenger MF, Cahill KS, Byrne BJ, Kessler PD (2002) Human mesenchymal stem cells differentiate to a cardiomyocyte phenotype in the adult murine heart. *Circulation* 105: 93 – 98

Tonkin J, Temmerman L, Sampson RD, Gallego-Colon E, Barberi L, Bilbao D, Schneider MD, Musarò A, Rosenthal N (2015) Monocyte/macrophage-derived IGF-1 orchestrates murine skeletal muscle regeneration and modulates autocrine polarization. *Mol Ther* 23: 1189 – 1200

Trostel J, Truong LD, Roncal-Jimenez C, Miyazaki M, Miyazaki-Anzai S, Kuwabara M, McMahan R, Andres-Hernando A, Sato Y, Jensen T *et al* (2018) Different effects of global osteopontin and macrophage osteopontin in glomerular injury. *Am J Physiol Renal Physiol* 315: F759 – F768

Wall IB, Moseley R, Baird DM, Kipling D, Giles P, Laffafian I, Price PE, Thomas DW, Stephens P (2008) Fibroblast dysfunction is a key factor in the non-healing of chronic venous leg ulcers. *J Invest Dermatol* 128: 2526 – 2540

Wang L, Wang C, Wang Z, Gan J, Liu C, Xia S, Niu Y, Chen D, Zhang J, Dong L (2020) Transforming the spleen into a liver-like organ *in vivo*. *Sci Adv* 6: eaaz9974

Wheeler KC, Jena MK, Pradhan BS, Nayak N, Das S, Hsu CD, Wheeler DS, Chen K, Nayak NR (2018) VEGF may contribute to macrophage recruitment and M2 polarization in the decidua. *PLoS One* 13: e0191040

Willenborg S, Sanin DE, Jais A, Ding X, Ulas T, Nüchel J, Popović M, MacVicar T, Langer T, Schultze JL *et al* (2021) Mitochondrial metabolism coordinates stage-specific repair processes in macrophages during wound healing. *Cell Metab* 33: 2398 – 2414

Wysoczynski M, Khan A, Bolli R (2018) New paradigms in cell therapy: repeated dosing, intravenous delivery, immunomodulatory actions, and new cell types. *Circ Res* 123: 138 – 158

Yao Y, Xu X-H, Jin L (2019) Macrophage polarization in physiological and pathological pregnancy. *Front Immunol* 10: 792

Yuan R, Geng S, Chen K, Diao N, Chu HW, Li L (2016) Low-grade inflammatory polarization of monocytes impairs wound healing. *J Pathol* 238: 571 – 583

Zuloff-Shani A, Adunsky A, Even-Zahav A, Semo H, Orenstein A, Tamir J, Regev E, Shinar E, Danon D (2010) Hard to heal pressure ulcers (stage III-IV): efficacy of injected activated macrophage suspension (AMS) as compared with standard of care (SOC) treatment controlled trial. *Arch Gerontol Geriatr* 51: 268 – 272

License: This is an open access article under the terms of the Creative Commons Attribution License, which permits use, distribution and reproduction in any medium, provided the original work is properly cited.

© 2022 The Authors
