# Supplementary material for: Tumor‐associated macrophages‐educated reparative macrophages promote diabetic wound healing
Source: EMBO Mol Med. 2022 Dec 21;15(2):e16671. doi: 10.15252/emmm.202216671 (PMC9906426; doi:10.15252/emmm.202216671)
Supplement: Supplementary file 8 [file EMMM-15-e16671-s006.pdf]

EMBO Molecular Medicine

Tumor-associated macrophages-educated reparative macrophages promote diabetic wound healing

Ruoyu Mu, Zhe Zhang, Congwei Han, Yiming Niu, Zhen Xing, Zhencheng Liao, Jinzhi Xu, Ningyi Shao, Guokai Chen, Junfeng Zhang, Lei Dong, and Chunming Wang
DOI: 10.15252/emmm.202216671

Corresponding author: Chunming Wang (cmwang@umac.mo) , Lei Dong (leidong@nju.edu.cn)

Review Timeline:

Submission Date:	29th Jul 22
Editorial Decision:	16th Aug 22
Revision Received:	7th Nov 22
Editorial Decision:	28th Nov 22
Revision Received:	29th Nov 22
Accepted:	2nd Dec 22

Editor: Jingyi Hou

Transaction Report:

(Note: With the exception of the correction of typographical or spelling errors that could be a source of ambiguity, letters and reports are not edited. Depending on transfer agreements, referee reports obtained elsewhere may or may not be included in this compilation. Referee reports are anonymous unless the Referee chooses to sign their reports.)

16th Aug 2022

Dear Prof. Wang,

Thank you again for submitting your work to EMBO Molecular Medicine. We have now heard back from the three referees who agreed to evaluate your manuscript. As you will see from the reports below, the referees acknowledge the potential interest of the study. Still, they raise a series of concerns, which we would ask you to address in a revision of the manuscript.

Without reiterating all the points raised in the reviews below, some of the more substantial issues are the following:
- Referees #1 and #2 had several questions/concerns regarding the experimental procedures, which need to be carefully addressed.
-Issues regarding controls, statistics, replicates, figure quality, and wound healing experiments must be convincingly addressed.

All other issues raised by the referees need to be satisfactorily addressed as well. Please feel free to contact me in case you would like to discuss in further detail any of the issues raised by the referees.

We would welcome the submission of a revised version within three months for further consideration. Please note that EMBO Molecular Medicine strongly supports a single round of revision. As acceptance or rejection of the manuscript will depend on another round of review, your responses should be as complete as possible.

EMBO Molecular Medicine has a "scooping protection" policy, whereby similar findings that are published by others during review or revision are not a criterion for rejection. Should you decide to submit a revised version, I do ask that you get in touch after three months if you have not completed it to update us on the status.

We are aware that many laboratories cannot function at full efficiency during the current COVID-19/SARS-CoV-2 pandemic and have therefore extended our "scooping protection policy" to cover the period required for a full revision to address the experimental issues. Please let me know should you need additional time, and also if you see a paper with related content published elsewhere.

Please read below for important editorial formatting and consult our author's guidelines for proper formatting of your revised article for EMBO Molecular Medicine.

I look forward to receiving your revised manuscript.

Use this link to login to the manuscript system and submit your revision: https://embomolmed.msubmit.net/cgi-bin/main.plex

Sincerely,
Jingyi

Jingyi Hou
Editor
EMBO Molecular Medicine

***** Reviewer's comments *****

Referee #1 (Comments on Novelty/Model System for Author):

Mu et al show that treatment of bone marrow-derived macrophages (BMDM) with conditioned medium (CM) from tumor-associated macrophages (TAMs) derived from a mouse sarcoma tumor acquire immunosuppressive, pro-angiogenic and fibroblast-activating properties. Remarkably, this results in promotion of wound healing in healing-impaired diabetic mice. They further identified nine factors that are present in the TAM-conditioned medium, which - when combined - have similar properties. Finally, they show that the same factors can induce healing-promoting activities in human macrophages.

This is an interesting study with translational relevance. However, there are also some problems with the manuscript as listed below:

General comment:
The quality of the figures needs improvement. The labelling is too small and in some cases it is not even possible to read the label on the screen at high magnification.

Specific comments:

1. Page 7: The authors used an S180 heterotypic tumor model for their studies. They explain later why they chose this model, but this should be explained at the beginning. In general, one would like to know if secretion of these pro-healing factors (at least the nine which were chosen for further studies) is a general feature of TAMs. Are they also secreted by TAMs from other mouse and human tumors? This should be tested for at least 2-3 other tumors (e.g. by PCR). If this is not a general feature of TAMs, the authors should not generally use the word "TAM-educated macrophages", but rather be more precise.

2. It is clear from the protocol that BMDM were cultured for 2 days in TAM-CM or in the other media. I assume that the CM were then removed and all cells were then cultured under the same conditions. This should be mentioned. Were the cells extensively washed after the removal of the TAM-CM? Some of the factors may bind to the matrix and may still be present. Please clarify.

3. Related to this question: It is mentioned on page 6 that the macrophages have a different cell shape at day 2 - is this day 2 after addition of the TAM-CM or day 2 after removal of the TAM-CM?

4. It is not clear at what time point the bulk and scRNA-seq experiments were performed. Two days after addition of the TAM-CM or later (e.g. 2 days after the TAM-CM was removed?). Please clarify. This is important, because it is not clear how long the healing-promoting activities are maintained after removal of the TAM-CM. This issue is even more relevant for the Luminex assay and the functional experiments with the CM - it is important that all factors that are present in the TAM-CM were removed.

How does the TAMEM-CM compare to TAM-CM in the different functional assays?

5. The Western blots shown in Fig. 3 should be performed in triplicate and the results should be quantified.

6. The wound healing experiments are particularly interesting and important. However, it is unclear how the authors guaranteed that the biomaterial scaffold remains on the wounds - they obviously did not use a dressing. The authors should specifically mention that they used macrophages from eGFP-expressing mice. This allows to follow their survival (Fig. S4F). In addition, they should show eGFP fluorescence in wound sections - this will show where the transplanted macrophages are located in the wounds at the different stages of healing.

7. The photomicrographs of the histological wound stainings (in all figures!) are of poor quality. They are also far too small and it is impossible to see the wounds in the overview. Please show larger wound pictures that include the complete wound bed and both wound edges (up to the first hair follicle). The wound edges should be indicated with arrows. In addition, the different parts of the wound should be labeled (e.g. G for granulation tissue, SC for scab etc).

8. Figure 4: An important control is missing - wounds from healthy mice treated with TAMEM scaffolds. Does this further promote healing in healthy mice? The extent of re-epithelialisation (Fig. 4F) should be quantified based on the histological stainings.

9. Fig. 4: The higher expression of Col1a1 and Tgfb1 together with the higher expression of a-SMA suggest a pro-fibrotic function of the TAMEMs. Therefore, it would be important to look at a later time point to determine if TAMEMs promote scar formation.

10. Page 18: Please add a reference at the end of the first paragraph.

11. The stainings in Fig. 5 and 6 are also not convincing. Please show larger photomicrographs that include the complete wound (see above). The number of CD31+ vessels should be quantified as well as the number of stabilized vessels, which are CD31+/a-SMA+. In addition, the number of a-SMA+ cells outside of the vessels (myofibroblasts) should be quantified based on the same stainings.

12. The selection and use of 9 cytokines as a replacement for the TAMEM-CM is a very good idea and strategy. Please verify that BMDM have indeed receptors for these factors.

13. Regarding the TAMEMC-m I have the same question as for the TAMEM-CM: It seems that the nine factors are still present in the CM that was used for experiments - please clarify. Does TAMEMC-m that is obtained after removal of these nine factors (and then conditioned for 2 days) still induce e.g. Spp1 and Gas6 expression?

14. Discussion: The use of TAMEM for the treatment of wounds is clearly better than the use of tumor-derived cells with regard to safety. However, TAMEMs could still have pro-tumorigenic functions (they express various pro-tumorigenic proteins..). This should at least be mentioned. This is relevant, because squamous cell carcinoma development in chronic wounds is an important clinical problem.

Referee #1 (Remarks for Author):

This is an interesting manuscript, but it requires major revision as listed in my comments to the reviewers. In particular, the wound healing experiments are not fully convincing.

Referee #2 (Comments on Novelty/Model System for Author):

It is concerning that all of the in vitro experiments and many of the in vivo experiments were conducted with only n=3 replicates, and also surprising that the results were statistically significant with such a low replicate number considering the highly variable nature of some of these assays. Repeating at least some of these experiments is strongly recommended.

Referee #2 (Remarks for Author):

This is an extremely creative and interesting study involving using TAMs to promote a pro-reparative phenotye in macrophages with therapeutics effects in murine models of diabetic wound healing. The manuscript would be improved with increased rigor and in-depth discussion of some surprising findings, as detailed below:

What was the rationale for culturing TAMs for 15 days prior to collection of conditioned media to use to prepare TAMEMs? Macrophages are well known to be highly plastic, so presumably they lost their TAM phenotype upon such a long culture period.

The comparison to TAMs in Fig. 2G, H, and I is quite important, so more details should be provided. For example, how many

differentially epxressed genes were there between TAMEMs and TAMs? It would be helpful to combine this analysis with that shown in Fig. 2A, so that the TAMEMs are directly compared to M0, M1, M2, and TAMs, to answer the question of whether the TAMEMs functionally resemble TAMs more closely than M1 or M2.

Fig. 3- In these experiments, conditioned media from TAMEMs (which were cultured in conditioned media from TAMs) or M0, M1, and M2 macrophages was added to new BMDMs. The question is whether the conditioned media was generated after first removing the polarizing stimuli to allow generation of conditioned media in the absence of these stimuli (i.e. TAM-conditioned media for the TAMEMs, IFNg+LPS for M1, and IL4+IL13 for M2). If not, then the results would be expected to be influenced by both the macrophage-conditioned media as well as the polarizing cytokines in which they were originally cultured. These same effects would influence the results of experiments with fibroblasts and endothelial cells too. This is a critical detail as all of these cytokines (and TAM-conditioned media) are well known to influence fibroblasts.

Fig. 3F- it is surprising that mouse macrophage-conditioned media caused human HUVECs to increase tube formation. Was the positive control human or mouse VEGF? Another important detail that is not listed in the methods or supplement is for how long this assay was conducted. The Matrigel assay is notoriously finicky and time dependent.

It is concerning that all of the in vitro experiments and many of the in vivo experiments were conducted with only n=3 replicates, and also surprising that the results were statistically significant with such a low replicate number considering the highly variable nature of some of these assays. Repeating at least some of these experiments is strongly recommended.

It is extremely surprising that culture of M0s or TAMEMs in electrospun gelatin scaffolds did not influence their phenotype (as measured by 2 M1 markers and 2 M2 markers) as shown in Suppl. Fig. 4C. At what time point was that experiment conducted? Were the scaffold-seeded cells compared to baseline M0's and TAMEM's, or to those cells cultured at the same time points? Also, it looks like scaffold-seeded cells could be different but the results did not achieve statistical significance because it was only n=3. This would not be considered a major concern because the authors did assess the phenotype of transplanted macrophages following in vivo transplantation (Fig. S4G) except that the authors are currently misrepresenting their findings (e.g. page 14: "Loading of TAMEMs onto the scaffolds did not affect their morphology (Fig. 4C), proliferation (fig. S4B), or polarization (fig. S4C).").

The analysis of how pre-polarization state influenced the resultant phenotype 3 days after transplantation (Supp. Fig. 4I, J) is super interesting and novel. I recommend moving to a main figure and thoroughly describing the results in the text.

The authors should add discussion on how it is possible that the TAMEMs were less plastic than M0, M1, or M2, which is shown in Suppl. Fig. 4I, J.

It looks like statistical comparisons between the wound healing effects of TAMEMs shown in Figs. 5 and 6 are made in comparison to the blank control but not to the other groups. Were wound healing, granulation tissue, vasculature, etc., significantly different between the TAMEMs and M2 groups?

It is very surprising that human macrophages accelerated mouse wound healing (Fig. 6). This point should be discussed.

Referee #3 (Comments on Novelty/Model System for Author):

This is an interesting study providing proof principle data for educating macrophages to promote wound healing with have potentials to be tested clinically. The models are appropriate and data seems solid.

Referee #3 (Remarks for Author):

The current manuscript investigated the role of TAM educated macrophages (TAMEMs) in wound healing of both type I and type II diabetic mouse models. The authors generated an in vitro assay to induce macrophages to TAMEMs using a mixture of cytokine. Although the similarity of TAMs and wound healing macrophages has been noticed previously, it provides an interesting finding, maybe for the first time, showed that TAMEMs have a strong wound healing function in promoting diabetic tissue repair. Bulk RNAseq and scRNAseq further supported their findings. This study also provided a new approach to induce wound healing macrophages in vitro, which is promising for clinical application. The following concerns need to be addressed to further improve the current manuscript:

1. Ly6G-Ly6C+ cells are generally recognized as monocytes. In figure S1A the author labelled Ly6C+ MHCIIhi cells as TAMs. Although the author cited a published paper (Movahedi K, et al, 2010), the original paper did not provide conclusive data showing that these cells are macrophages, not monocytes. GIMSA staining should be performed to confirm that these cells are indeed macrophages to support the nomenclature. At the same time, it may worth showing Ly6C expression in different subsets in scRNAseq data.
2. M1/M2 nomenclature is oversimplified when describing macrophage phenotypes in vivo as demonstrated by numerous

studies and recent reviews. The author provided 1 marker for M2-phenotype to claim that "TAMEMs could efficiently shape the constitution of local host macrophages towards M2-dominant" in figure S6A&B. A more comprehensive phenotypic characterization is required to define the TAMEMs, TAMEMs induced/recruited local host macrophages and compare with wound healing macrophages.
3. In figure S9, the author showed the cocktail treatment is not as efficient as TAMEMs in wound healing. Any possible reason for this effect? Maybe the number and phenotype of macrophages around the wound were different, which can be quantified using FACS?

Minor points
1. Clarify the methods and the number of biological replication for the detection and quantification in figure 1C
2. Add representative genes for TAMEMs in figure 2E
3. The figure legend and figure title are different in Figure 5C. (Legend: monocytes, title: macrophages)
4. For figure 6B, a volcano plot with the number of up/down-regulated genes could be used to show the difference between TAMEM and TAMEMC-M
5. Some figures, especially microscope images are in low resolution.

Referee #1 (Comments on Novelty/Model System for Author):

Mu et al show that treatment of bone marrow-derived macrophages (BMDM) with conditioned medium (CM) from tumor-associated macrophages (TAMs) derived from a mouse sarcoma tumor acquire immunosuppressive, pro-angiogenic and fibroblast-activating properties. Remarkably, this results in promotion of wound healing in healing-impaired diabetic mice. They further identified nine factors that are present in the TAMs-conditioned medium, which - when combined - have similar properties. Finally, they show that the same factors can induce healing-promoting activities in human macrophages.

This is an interesting study with translational relevance. However, there are also some problems with the manuscript as listed below:

General comment:
The quality of the figures needs improvement. The labelling is too small and in some cases it is not even possible to read the label on the screen at high magnification.
We appreciate the suggestion and have improved our figure quality in the revised manuscript.

Specific comments:

Q1. Page 7: The authors used an S180 heterotypic tumor model for their studies. They explain later why they chose this model, but this should be explained at the beginning. In general, one would like to know if secretion of these pro-healing factors (at least the nine which were chosen for further studies) is a general feature of TAMs. Are they also secreted by TAMs from other mouse and human tumors? This should be tested for at least 2-3 other tumors (e.g. by PCR). If this is not a general feature of TAMs, the authors should not generally use the word "TAMs-educated macrophages", but rather be more precise.
A1: We thank the reviewer for these valuable suggestions and have made the following changes accordingly:

1) We have moved the reason for choosing the S180 model (based on findings from our previous study, *Science Advances, 2020*[1]) to the **first paragraph** of Results, immediately following the Introduction in the revised manuscript. We concisely report that the tumor homogenate from S180 outperformed the homogenate from several other tumors, such as Hepa1-6 (hepatoma), 4T1(breast cancer), and B16-F10 (melanoma), in inducing the expression of IL-10 in murine BMDMs and type I collagen in mouse embryonic fibroblasts (MEFs);
2) As the reviewer suggests, **we have added new data** by testing the expression of the **9** proteins in TAMs from three other tumors – two mouse models (4T1 breast tumor and B16-F10 melanoma) and one human model (MCF-7 breast cancer), as shown in the new **Fig EV4C**. All these TAMs expressed these 9 proteins. Their levels were consistently higher than in BMDM-M0 in general but also showed differences. S180-derived TAMs presented the highest levels in most proteins, 4T1- and MCF-7-derived TAMs had comparable levels (though slightly lower in Il31 and CSF1), and B16-F10-oderived TAMs appeared weaker – especially in *Il10* and *Vegfb*

– albeit not far too lower. This pattern suggested that these factors were not exclusively produced by S180-derived TAMs. TAMs from other tumors might also have a similar feature (as a common advantage), though we chose S180 as an optimal protocol in this study.

Q2. It is clear from the protocol that BMDM were cultured for 2 days in TAMs-CM or in the other media. I assume that the CM were then removed and all cells were then cultured under the same conditions. This should be mentioned. Were the cells extensively washed after the removal of the TAMs-CM? Some of the factors may bind to the matrix and may still be present. Please clarify.

A2: We apologize for not having clearly described the experimental process in the original manuscript. **We have now improved the description** in the revised experimental methods.

1) Our practice was: first, we cultured primary BMDMs for 2 days in the medium containing TAMs-CM or other stimuli, to obtain TAMEMs or other macrophage subsets, respectively. Next, we removed the old medium, **washed the cells twice gently but adequately with PBS**, and made one of the three choices:
    A. directly processed the cells for bulk/sc RNAseq, FACs, IF, etc, for cell analysis; or
    B. added the same RPMI-1640 complete medium, and cultured for another **2 days** (for collection of supernatant); or
    C. added the same RPMI-1640 complete medium, and cultured for another **4 days** (for observing cell morphology and other purposes).
2) As the reviewer is concerned, after Choice B), i.e., **2 days** in fresh medium, we collected the medium as TAMEM-CM, M1-CM, M2-CM, and M0-CM for subsequent functional analyses;
3) We believe that binding of these factors to the matrix was minimal, because the two-time wash (gentle but thorough) could well remove them. In many other studies, cell stimuli were also removed by washing twice with PBS (*Nature Methods, 2006*[2], *Clinical Cancer Research, 2007*[3]), which is a commonly accepted practice. Our subsequent experiments also identified nine soluble proteins as the effective factors, which should be readily rinsed off by our protocol.

Q3. Related to this question: It is mentioned on page 6 that the macrophages have a different cell shape at day 2 - is this day 2 after addition of the TAMs-CM or day 2 after removal of the TAMs-CM?

A3: We have improved the description in **our revised manuscript**.

1) This Day 2, as termed in the **original Fig 1B**, referred to BMDMs having been cultured in TAMs-CM for 2 days. At the end of 'this day 2', we removed all CM, washed the cells twice gently with PBS, and cultured them in RPMI-1640 complete medium for another 4 days to obtain TAMEMs and other control cells (please also refer to the Choice C of the 'three choices' in our response to Q2 above). So, we obtained all cells on 'day 6' in our original method of description;
2) Given the confusion caused by this description, we thought it better to nominate the day of acquiring TAMEMs as 'day 0'. So, we changed the original 'day 2' to 'day 0'

– the day we obtained TAMEMs and control macrophages after 2 days of CM simulation – and the original 'day 6' to 'day 4', as shown in the **new Fig 1B**.

We hope the new description made the timeline clearer to the Reviewer and readers.

Q4. It is not clear at what time point the bulk and scRNA-seq experiments were performed. Two days after addition of the TAMs-CM or later (e.g. 2 days after the TAMs-CM was removed?). Please clarify. This is important, because it is not clear how long the healing-promoting activities are maintained after removal of the TAMs-CM. This issue is even more relevant for the Luminex assay and the functional experiments with the CM - it is important that all factors that are present in the TAMs-CM were removed. How does the TAMEMs-CM compare to TAMs-CM in the different functional assays?

A4: We are sorry for the unclarity and wish to clarify below:

1) The bulk and scRNA-seq analysis of TAMEMs were performed two days after BMDMs were incubated in TAMs-CM and washed with PBS twice. Please refer to the Choice A of our 'three choices' stated in our response to Q2 above for our protocols;
2) TAMEMs could maintain the morphology (the new **Fig 1B**) and phenotype (the new **Fig EV1C**), after the removal of TAMs-CM and subsequent culture in RPMI-1640 complete medium for 4 days (Choice C of our 'three choices');
3) The TAMEM-CM, M2-CM, M1-CM, and M0-CM for the Luminex assay and the functional experiments were collected after 2 days of culturing TAMEMs or other control cells with the same complete medium (Choice B of our 'three choices');
4) To answer the last question of the reviewer, we **have added new experiments** to compare the functions of TAMs-CM and TAMEM-CM. As the new **Fig EV2C** and **D** show, TAMs-CM was stronger than TAMEM-CM in inducing immunosuppression and activating fibroblasts.

Q5. The Western blots shown in Fig. 3 should be performed in triplicate and the results should be quantified.

A5: We have re-performed the Western blots with 3 biological replicates and performed a statistical analysis in the new **Fig 3G** and **N**.

Q6. The wound healing experiments are particularly interesting and important. However, it is unclear how the authors guaranteed that the biomaterial scaffold remains on the wounds - they obviously did not use a dressing. The authors should specifically mention that they used macrophages from eGFP-expressing mice. This allows to follow their survival (Fig. S4F). In addition, they should show eGFP fluorescence in wound sections - this will show where the transplanted macrophages are located in the wounds at the different stages of healing.

A6: We thank the reviewer for these valuable questions and our responses are below:

1) We apologize for not having described the experimental details clearly in the original manuscript. In fact, we applied the transparent dressing (Tegaderm™ Film, 3M) on the wound. If we did not use the dressing, the scaffolds would fall off. The reviewer assumed that we did not use a dressing, possibly because we peeled it off when taking

photos (otherwise it reflected light and affected image clarity). We changed the dressing every other day. We have revised it in the revised manuscript;

2) We have **added new experiments** to analyze the localization of transplanted eGFP$^+$ cells at different stages of wound healing. As shown in **Appendix Fig S2F**, at day 1 post-wounding, most eGFP$^+$ cells loaded on the electrospun scaffolds were concentrated on the surface of the wound. At day 3, the eGFP$^+$ cells gradually migrated to the newly formed granulation tissue, though some of them were still in the scar tissue on the surface of the wound. Eventually, all the eGFP$^+$ cells on the wound surface entered the granulation tissue. The transplanted cells gradually decreased, as observed such as at day 5. In the description of **Appendix Fig S2F**, we have especially mentioned the use of C57BL/6 eGFP$^+$ mice to obtain cells for transplantation.

Q7. The photomicrographs of the histological wound stainings (in all figures!) are of poor quality. They are also far too small and it is impossible to see the wounds in the overview. Please show larger wound pictures that include the complete wound bed and both wound edges (up to the first hair follicle). The wound edges should be indicated with arrows. In addition, the different parts of the wound should be labeled (e.g. G for granulation tissue, SC for scab etc).

A7: This is an important reminder. We have **updated all photomicrographs** of the histological wound staining and added relevant labels as recommended by the reviewer, including the new **Fig 4G, Fig 5E, Fig 7D, Fig EV3A, Fig EV3B, Fig EV5A, Fig EV5D, and Appendix Fig S6B.**

Q8. Figure 4: An important control is missing - wounds from healthy mice treated with TAMEM scaffolds. Does this further promote healing in healthy mice? The extent of re-epithelialisation (Fig. 4F) should be quantified based on the histological staining.

A8:

1) As early as the beginning of this project, we had already validated the efficacy of TAMEMs in healthy mice, as shown in **Fig. R1** below. These data, observed also through 14 days, were consistent with our findings from both Type I and II diabetic mice models, showing that TAMEMs more efficiently promoted wound healing than did other macrophage subtypes. However, considering two factors that – i) the goal of this project focuses on overcoming the pathological obstacles with diabetic wounds (which is an unmet medical challenge), while healing under normal physiological conditions has more and simpler solutions; and ii) the data set (including Appendix Figures) of this paper is already very large – we decided not to show this very early part of tests. The readers of this paper can read this peer-review document and communicate with us should they be interested in these preliminary experiments;

Figure for reviewers removed.

2) We have added the quantification of re-epithelialization based on the histological staining, as shown in the new **Fig 4I**.

Q9. Fig. 4: The higher expression of Col1a1 and Tgfb1 together with the higher expression of a-SMA suggest a pro-fibrotic function of the TAMEMs. Therefore, it would be important to look at a later time point to determine if TAMEMs promote scar formation.
A9: We thank the reviewer for this suggestion and have **added new experiments** to see if TAMEMs promote scar formation. As shown in **Fig. R2A**, TAMEMs did not cause more scar formation than the situation in the blank control till a prolonged period of 42 days. As shown in **Fig. R2B**, the transplanted TAMEMs were hardly detectable at the wound site on day 7 post-transplantation. These findings support that TAMEMs mainly work in the early healing stages.

Figure for reviewers removed.

Q10. Page 18: Please add a reference at the end of the first paragraph.

A10: We have added a new reference (*Arteriosclerosis, Thrombosis, and Vascular Biology, 2018*[4]) in the revised manuscript.

Q11. The staining in Fig. 5 and 6 are also not convincing. Please show larger photomicrographs that include the complete wound (see above). The number of CD31+ vessels should be quantified as well as the number of stabilized vessels, which are CD31+/a-SMA+. In addition, the number of a-SMA+ cells outside of the vessels (myofibroblasts) should be quantified based on the same staining.

A11: We appreciate these valuable suggestions. We have **updated all staining** in the new Fig. 5 and 6 and replaced the fluorescence gray value with the number of blood vessels to quantify neovascular and mature vessels in the new **Fig 5F** and **Fig EV5E**.

Q12. The selection and use of 9 cytokines as a replacement for the TAMEM-CM is a very good idea and strategy. Please verify that BMDM have indeed receptors for these factors.

A12: This is an excellent suggestion! We have thoroughly acquired evidence for these factors:

1) We have first figured out the receptors corresponding to each cocktail component as:
    a. OPN receptors include two families: integrin and CD44; the former include alpha(v)beta(3), alpha(v)beta(1), alpha(v)beta(5), alpha(9)beta(1), and alpha(4)beta(1) [5];
    b. IL-31 sends signals through a receptor complex made of IL-31RA and oncostatin M receptor β (OSMRβ) expressed in immune and epithelial cells [6];
    c. the receptor of IL-10 is IL-10Ra;
    d. TGFβ2 can be recognized by TGFβR3 [7];
    e. MIP-2 functions through the chemokine receptor CXCR2;
    f. CCL8 elicits its effects by binding to several different cell surface receptors called chemokine receptors. These receptors include CCR1, CCR2B, CCR3 and CCR5 [8];
    g. Macrophage colony-stimulating factor (M-CSF), is a secreted cytokine which causes hematopoietic stem cells to differentiate into macrophages, it binds to the colony stimulating factor 1 receptor (CSF1R);
    h. VEGFB is one of the VEGF family, it can only bind VEGFR1 [9];
    i. The receptors for members of the fibroblast growth factor (FGF) family of proteins are fibroblast growth factor receptors (FGFR). Of these receptors, FGF2 has the highest affinity for FGFR1, 2 and 3b [10].
2) Then, we have checked our **RNA-seq data** for unpolarized macrophages (M0) and quantified the expression of these receptors. As shown in **Fig. R3** below, unpolarized macrophages express the receptors for the cocktail components. Meanwhile, several previous studies supported our findings (*Journal of Biological Chemistry, 1996*[11]; *Journal of Leukocyte Biology, 2001*[12]; *Developmental & Comparative Immunology, 2020*[13]).

Figure for reviewers removed.

Q13. Regarding the TAMEMC-m I have the same question as for the TAMEM-CM: It seems that the nine factors are still present in the CM that was used for experiments - please clarify. Does TAMEMC-m that is obtained after removal of these nine factors (and then conditioned for 2 days) still induce e.g. Spp1 and Gas6 expression?

A13:

1) As we replied to Q2 and Q3 above, we obtained $TAMEMs^{C\text{-}m}$ or other control cells by culturing BMDMs for 2 days in the medium containing recombinant protein cocktail or other stimuli. After that, we removed the supernatant, washed the cells gently but thoroughly twice with PBS, and then added RPMI-1640 complete medium for further experiments. The nine soluble factors should not exist in the latter medium;
2) We have **added new experiments** to investigate whether TAMEMs could maintain phenotypic stability, mainly the expression of *Spp1* and *Gas6*, after the removal of recombinant proteins. Like the TAMEMs, $TAMEMs^{C\text{-}m}$ could maintain the phenotype (**Fig. R4**) after removing recombinant protein cocktail and culturing in RPMI-1640 complete medium for 4 days.

Figure for reviewers removed.

Q14. Discussion: The use of TAMEM for the treatment of wounds is clearly better than the use of tumor-derived cells with regard to safety. However, TAMEMs could still have pro-tumorigenic functions (they express various pro-tumorigenic proteins..). This should at least be mentioned. This is relevant, because squamous cell carcinoma development in

chronic wounds is an important clinical problem.

A14: This is an insightful question!

1) Cell therapy has a promising future in regenerative medicine, but its tumorigenicity is of substantial concern, e.g. pluripotent stem cells [14]. Therefore, it is crucial to assess TAMEMs in this regard. We found that, although TAMEMs expressed certain genes related to oncogenic activities, such as *Raf1*, *Araf*, *Rab5b*, etc, the level was not significantly higher than in 'common' macrophages subtypes M0, M1 and M2. Meanwhile, we tested the remaining number of transplanted cells on day 7 after transplantation, as shown in **Fig. R2B** above. There was almost no detectable transplanted TAMEMs on day 7. Such data suggest that the tumorigenic risk of the transplanted TAMEMs forming its own tumors is very low.
2) However, in designing our study towards future translation, we also considered avoiding even the minimal tumorigenic risk – this was why we decided to identify the action factors and reconstitute a cocktail. By using this cocktail to train to-be-delivered macrophages (TAMEMs^{C}), we can avoid the therapeutic cells from directly contacting any tumor-derived components, thereby increasing the safety of this technology and potential for clinical application.
3) Combining our findings and the reviewer's suggestions, we have added more discussion in the revised manuscript.

Referee #1 (Remarks for Author):

This is an interesting manuscript, but it requires major revision as listed in my comments to the reviewers. In particular, the wound healing experiments are not fully convincing.

We totally agree and thank the reviewer for these suggestions, which have greatly improved our study.

Referee #2 (Comments on Novelty/Model System for Author):

It is concerning that all of the in vitro experiments and many of the in vivo experiments were conducted with only n=3 replicates, and also surprising that the results were statistically significant with such a low replicate number considering the highly variable nature of some of these assays. Repeating at least some of these experiments is strongly recommended.

We thank the reviewer and have added new replicate experiments, as described below.

Referee #2 (Remarks for Author):

This is an extremely creative and interesting study involving using TAMs to promote a pro-reparative phenotype in macrophages with therapeutics effects in murine models of diabetic wound healing. The manuscript would be improved with increased rigor and in-depth discussion of some surprising findings, as detailed below:

Q1. What was the rationale for culturing TAMs for 15 days prior to collection of conditioned media to use to prepare TAMEMs? Macrophages are well known to be highly plastic, so presumably they lost their TAMs phenotype upon such a long culture period.

A1:

(1) As mentioned in the manuscript, we developed our protocol of harvesting and culturing TAMs in reference to the method by van Ginderachter's group (*Cancer Research* 2010) with minor revisions. We defined TAMs into five sub-populations: i) $Ly6C^{hi}MHC\ II$ Monocytes, ii) $Ly6C^{hi}MHCII^{int}$ TAMs, iii) $Ly6C^{int}MHCII^{hi}$ TAMs, iv) $Ly6C^{low}MHCII^{hi}$ TAMs and v) $Ly6C^{low}MHC\ II^{low}$ TAMs. $Ly6C^{hi}$ monocytes as precursors of TAMs differentiate into $Ly6C^{int}$ TAMs after 6 days and all differentiate into $Ly6C^{low}MHCII^{hi}$ TAMs and $Ly6C^{low}MHCII^{low}$ TAMs after 12 days. Importantly, the literature also states that $Ly6C^{low}MHCII^{low}$ TAMs showed strong pro-angiogenic activity and $Ly6C^{low}MHCII^{hi}$ TAMs with M1 macrophage genotype has low antigen presentation ability, and can effectively inhibit T cell proliferation and activation, showing the ability to inhibit inflammatory response. So, we finally set the culture time at 15 days for collecting more pro-angiogenic and anti-inflammatory components, as it is better to culture sorted TAMs for more than 12 days to obtain more $Ly6C^{low}MHC\ II^{hi}$ TAMs and $Ly6C^{low}MHC\ II^{low}$ TAMs, but at the same time to avoid too long isolation time which leads to the death of primary cells releasing some apoptotic signals.

(2) It was also confirmed in our pre-test that IL-10 in the TAMs culture supernatant gradually increased time (1, 5, 10, and 15 days) and that apoptosis occurred beyond 15 days of culture.

Q2. The comparison to TAMs in Fig. 2G, H, and I is quite important, so more details should be provided. For example, how many differentially epxressed genes were there between TAMEMs and TAMs? It would be helpful to combine this analysis with that shown in Fig. 2A, so that the TAMEMs are directly compared to M0, M1, M2, and TAMs, to answer the question of whether the TAMEMs functionally resemble TAMs more closely than M1 or M2.

A2: As the reviewer suggests, we have **added new data** about the differential gene analysis between TAMEMs and TAMs. Compared with TAMEMs, TAMs highly expressed many genes related to glycolysis and tumor metastasis, as shown in the **Fig EV1J.** TAMEMs and TAMs shared fewer differentially expressed genes (257) than 706 between TAMEMs and M0, 5178 between TAMEMs and M1, and 1756 between TAMEMs and M2, which is consistent with the correlation analysis in **Fig 2H**. This part of the data, derived from single-cell RNA sequencing, were less suitable to be combined with Fig. 2A which are data from bulk RNA sequencing. We therefore present it in Supplementary data.

Q3. Fig. 3- In these experiments, conditioned media from TAMEMs (which were cultured in conditioned media from TAMs) or M0, M1, and M2 macrophages was added to new BMDMs. The question is whether the conditioned media was generated after first removing the polarizing stimuli to allow generation of conditioned media in the absence of these stimuli (i.e. TAMs-conditioned media for the TAMEMs, IFNg+LPS for M1, and IL4+IL13 for M2). If not, then the results would be expected to be influenced by both the macrophage-conditioned

media as well as the polarizing cytokines in which they were originally cultured. These same effects would influence the results of experiments with fibroblasts and endothelial cells too. This is a critical detail as all of these cytokines (and TAM-conditioned media) are well known to influence fibroblasts.

A3: Yes, the conditional media was generated after first removing the polarizing stimuli. As we explained in response to Reviewer 1's Q2 above, our practice was: TAMEMs or M0, M1, and M2 macrophages were obtained by culturing primary BMDMs for 2 days in the medium containing TAMs-CM or other stimuli (LPS and IFN-γ for M1, and IL4 and IL13 for M2), respectively. Then, we removed the old medium (containing stimuli), washed the cells twice with PBS, added the same RPMI-1640 complete medium, and cultured for another 2 days for collecting the conditional media. Thus, the conditional medium used for further experiments was free of the inducers.

Q4. Fig. 3F- it is surprising that mouse macrophage-conditioned media caused human HUVECs to increase tube formation. Was the positive control human or mouse VEGF? Another important detail that is not listed in the methods or supplement is for how long this assay was conducted. The Matrigel assay is notoriously finicky and time dependent.

A4: This is a very interesting question!

1) The positive control was mouse VEGF, to maintain consistency in our experiments. According to the manufacturer's instructions for the mouse VEGF recombinant protein we used, it can promote human-origin HUVEC proliferation dose dependently (Biolegend, Cat # 583104). Also, other scientists often observed that mouse-derived VEGF can stimulate human cells (e.g. *Nature Communications, 2021*[15]). This is reasonable considering that mouse and human VEGF receptors are highly homologous, up to 85%[16];
2) Despite such literature/knowledge evidence, **this question triggered our curiosity to do an additional experiment**. We repeated the tube formation assay using **mouse**-derived endothelial cells (SVEC4-10), as shown in the new **Fig 3H**. Unsurprisingly, the **mouse**-derived macrophage CM promoted tube formation better in **mouse** SVEC4-10 than in human HUVEC. We have therefore replaced the previous data from HUVEC with data from SVEC4-10, updated in **Fig 3H.**
3) The HUVECs was seeded on the Matrigel (Corning) and treated with different CM. After incubation at 37 °C for 4 h, the cells were stained with Calcein AM, followed by observation and imaging of the tube formation. As we have changed the cells to SVEC4-10 in this revision, we have updated the description in the **revised Methods**.

Q5. It is concerning that all of the in vitro experiments and many of the in vivo experiments were conducted with only n=3 replicates, and also surprising that the results were statistically significant with such a low replicate number considering the highly variable nature of some of these assays. Repeating at least some of these experiments is strongly recommended.

A5:

1) Having re-checked the replications of all experiments, we **have repeated many *in vitro*** experiments to fulfil **6 biological** replicates, including: the viability curve of TAMEMs and typical macrophages subtypes (updated in the new **Fig 1C**), the

immunofluorescent staining and flow cytometry analysis for CD86 and CD206 in TAMEMs and typical macrophages subtypes (updated in the new **Fig 1E** and **F**), the immunofluorescent staining, flow cytometry analysis, and the real-time qPCR analysis for CD86 and CD206 in BMDMs that were cultured with conditional medium (CM) from TAMEMs, M2, M1, M0, and normal medium (updated in the new **Fig 3B-D**), the real-time qPCR analysis of *Il6* and *Cxcl15* in fibroblasts (L929) that pre-treated with LPS and then cultured with CM from TAMEMs and other controls (updated in the new **Fig 3E**), and some functional assays of TAMEMs-CM, such as the migration, proliferation, and the production of collagen of L929 cells treated with different CMs (updated in the new **Fig 3I-L** and **O**).

2) For *in vivo* tests, most had already had 8 replicates, such as all animal experiments on diabetic wound healing (including T1D/T2D and immunodeficient murine diabetic models) (e.g. **Fig 4E-G, Fig 5E, etc.**). Flow cytometry ones had 5 replicates (flow cytometry analysis of macrophage phenotypes in diabetic wounds) (e.g. **Fig 5A-C, etc.**). Only RT-qPCR and vessel perfusion assay had 3 replicates (e.g. **Fig 4K, Fig 5D Fig 5G, etc.**).

Q6. It is extremely surprising that culture of M0s or TAMEMs in electrospun gelatin scaffolds did not influence their phenotype (as measured by 2 M1 markers and 2 M2 markers) as shown in Suppl. Fig. 4C. At what time point was that experiment conducted? Were the scaffold-seeded cells compared to baseline M0's and TAMEM's, or to those cells cultured at the same time points? Also, it looks like scaffold-seeded cells could be different but the results did not achieve statistical significance because it was only n=3. This would not be considered a major concern because the authors did assess the phenotype of transplanted macrophages following in vivo transplantation (Fig. S4G) except that the authors are currently misrepresenting their findings (e.g. page 14: "Loading of TAMEMs onto the scaffolds did not affect their morphology (Fig. 4C), proliferation (fig. S4B), or polarization (fig. S4C).").

A6:

1) Yes, the scaffold-seeded cells were compared to those cells cultured at the same time points. The detailed procedure was: we obtained M0 (BMDMs) by culturing bone marrow-derived cells with M-CSF for 7 days and TAMEMs by continuing to culture M0 with TAMs-CM for 2 days. After that, M0 and TAMEMs are divided into two groups, respectively: one group seeded on the culture plate and the other on the electrospun scaffolds, followed by culture in the same RPMI-1640 complete medium for 2 days. At the end of these 2 days, the change folds of M1 and M2 markers in both scaffold-cultured and plate-cultured cells were all compared with plate-cultured M0, as analyzed by qPCR, using *t*-test. More details of the group comparison are given in the legend of **Appendix Fig S2C**;

2) We have re-performed this part of experiment and included 6 biological replicates in a group, as shown in the updated **Appendix Fig S2C**.

Q7. The analysis of how pre-polarization state influenced the resultant phenotype 3 days after transplantation (Supp. Fig. 4I, J) is super interesting and novel. I recommend moving to a main figure and thoroughly describing the results in the text.

A7: We thank the reviewer for the appreciation. We have reorganized this part of data (the original fig. S4J) and moved part of them in the new **Fig 4D** in the revised manuscript, taking into consideration both the data importance and the image layout for presentation. We have also thoroughly described the results the revised manuscript.

Q8. The authors should add discussion on how it is possible that the TAMEMs were less plastic than M0, M1, or M2, which is shown in Suppl. Fig. 4I, J.

A8: This is another insightful question. We have added relevant discussion in the revised manuscript and elaborate a bit here for exchanging ideas with the reviewer and potential readers.

1) First, diabetic wounds have a collectively pro-inflammatory environment rich in various inflammatory cytokines and possibly pathogens. In such environment, naive macrophages (M0) are prone to activation. M2 macrophages are also susceptible to switching into inflammatory phenotypes by inflammatory stimuli or bacterial pathogens [17]. This is consistent with what we have observed (Fig 4D and **Appendix Fig S2**I).
2) Meanwhile, we also found that the transplanted M1 macrophages are less responsive to diabetic wound inflammation. We suspect that the reason for this phenomenon is immune tolerance. Exposure to microbial or bacterial lipopolysaccharide (LPS) and other microbial components can enhance macrophage-mediated immune tolerance, resulting in epigenetic changes [18].
3) Interestingly, we also found a similar phenomenon of immune tolerance on TAMEMs. So, we speculate that one or some components of TAMs-CM alone or cooperatively change the epigenetics of TAMEM, thus causing the tolerance or stability of TAMEM. In the following work, we will detect the epigenetic changes of TAMEMs and explore the reasons for establishing immune tolerance and stability.

Q9. It looks like statistical comparisons between the wound healing effects of TAMEMs shown in Figs. 5 and 6 are made in comparison to the blank control but not to the other groups. Were wound healing, granulation tissue, vasculature, etc., significantly different between the TAMEMs and M2 groups?

A9: We are grateful to this important reminder and have added the statistical difference analysis between TAMEMs and M2 to these figures in the revised manuscript. The data showed that these outcomes were significantly different between these two groups.

Q10. It is very surprising that human macrophages accelerated mouse wound healing (Fig. 6). This point should be discussed.

A10: We also found it interesting and have added relevant discussion in the revised manuscript.

1) It has been shown that mice can support the survival of transplanted human immune cells, and in turn the transplanted cells exert biological functions in mice. Other

scientists have also demonstrated that transplanted **human** immune cells could function during repair of multiple tissue types in mice, e.g. in the skin [19] and colon [20] of mice. For instance, McKay et al. showed that **human** interleukin 4-treated regulatory macrophages promoted epithelial repair and reduce colitis **in mice** [20].

2) One possible reason is that, in our experiments, macrophages exerted repairing activities through secretion of a plethora of cytokines and growth factors. It is already known that many key cytokines involved in wound healing, such as VEGF, PDGF and IL-4, are up to 90% homologous in mice and humans, according to data from MGI (The international database resource for the laboratory mouse). This is also why mice, which share 99% genes and common pathologies with humans, are a good scientific animal model [21].

We thank Reviewer 2 again for triggering our thoughts and interesting discussion.

<u>Referee #3</u> (Comments on Novelty/Model System for Author):

This is an interesting study providing proof principle data for educating macrophages to promote wound healing with have potentials to be tested clinically. The models are appropriate and data seems solid.

We thank the reviewer for the encouraging comments.

Referee #3 (Remarks for Author):

The current manuscript investigated the role of TAM educated macrophages (TAMEMs) in wound healing of both type I and type II diabetic mouse models. The authors generated an in vitro assay to induce macrophages to TAMEMs using a mixture of cytokine. Although the similarity of TAMs and wound healing macrophages has been noticed previously, it provides an interesting finding, maybe for the first time, showed that TAMEMs have a strong wound healing function in promoting diabetic tissue repair. Bulk RNAseq and scRNAseq further supported their findings. This study also provided a new approach to induce wound healing macrophages in vitro, which is promising for clinical application. The following concerns need to be addressed to further improve the current manuscript:

Q1. Ly6G-Ly6C+ cells are generally recognized as monocytes. In figure S1A the author labelled Ly6C+ MHCIIhi cells as TAMs. Although the author cited a published paper (Movahedi K, et al, 2010), the original paper did not provide conclusive data showing that these cells are macrophages, not monocytes. GIMSA staining should be performed to confirm that these cells are indeed macrophages to support the nomenclature. At the same time, it may worth showing Ly6C expression in different subsets in scRNAseq data.

A1:

(1) We thank the reviewer for pointing out the accurate definition of TAMs in this context. We agree with the reviewer that $Ly6C^{+}$ cells are generally recognized as monocytes. So the $Ly6C^{hi}MHC\ II^{-}$ TAMs sorted by the Ginderachter's group-based method could be

monocytes – but all eventually differentiate into the two populations of macrophages ($Ly6C^{low}MHC\ II^{hi}$ TAMs and $Ly6C^{low}MHC\ II^{low}$). We have added the GIMSA staining to confirm that the suspended monocytes would differentiate into adherent macrophages after 15 days in culture (**Appendix Fig S1B**).

(2) We also present the Ly6c expression in scRNAseq data derived from freshly isolated TAMs, as shown in the **Fig EV1K**. The results were also consistent with the flow cytometry results, which included $Ly6C^{Hi}$, $Ly6C^{Int}$, and $Ly6C^{Low}$ cells.

Q2. M1/M2 nomenclature is oversimplified when describing macrophage phenotypes in vivo as demonstrated by numerous studies and recent reviews. The author provided 1 marker for M2-phenotype to claim that "TAMEMs could efficiently shape the constitution of local host macrophages towards M2-dominant" in figure S6A&B. A more comprehensive phenotypic characterization is required to define the TAMEMs, TAMEMs induced/recruited local host macrophages and compare with wound healing macrophages.

A2: As suggested by the reviewer, the phenotype of macrophages in the physiological state is far more complex than that of M1/M2 macrophages induced in vitro. Thus, a single marker is insufficient to describe the phenotype of macrophages induced by transplanted TAMEMs.

1) We have **added new data** on analyzing six markers – including two surface markers and four non-surface-protein genes – to define the phenotype of TAMEMs induced macrophages in situ in wounds, compared with the macrophages involved in the wound healing process in normal/diabetic mice. Here, we have added CCR2 and CD163, which were used to define TAMEMs in **Fig 1E**. $CCR2^{+}$ macrophages are critical for angiogenesis in tissue repair [22], and CD163 is also considered to be a typical marker of M2, while $CD163^{+}$ macrophages also play an important role in wound healing [23]. As shown in the new **Appendix Fig S3E** and **F**, the addition of TAMEMs increases the number of both of these macrophages, which are lacking in diabetic mice during wound healing, in on day 7 after wounding.
2) In addition to cell membrane proteins, we have measured the expression of 4 genes, including *Il1b* and *Ptgs*, whose expression peaks in earlier stages of healing but decreases later, and *Mgl2* and *Clec10a*, which have an opposite trend of expression [24], in macrophages at day 7 post wound healing that were sorted out by flow sorting. As shown in the new **Appendix Fig S3G**, TAMEMs addition reduces the expression of *Il1b* and *Ptgs* in macrophages, which are highly expressed in diabetic wound macrophages, and increases the expression of *Mgl2* and *Clec10a* in macrophages, which are lowly expressed in diabetic wound macrophages. These data better validate our view that TAMEMs could efficiently shape the constitution of local host macrophages towards immunosuppressive.

Q3. In figure S9, the author showed the cocktail treatment is not as efficient as TAMEMs in wound healing. Any possible reason for this effect? Maybe the number and phenotype of macrophages around the wound were different, which can be quantified using FACS?

A3: This is an insightful assumption. We are glad to share our findings here:

1) We developed cell therapy instead of using cytokine cocktails, considering the complicated and challenging microenvironment of diabetic wounds. The one-time

delivery of cytokines in the form of recombinant proteins supplied in the cocktail can rapidly degrade and diffuse *in vivo*, which is a common issue, and cannot sustain to direct the phenotypic change of local macrophages;

2) We have **added new data** to support our speculation, as shown in the **new Appendix Fig S6D-F**. First, on day 7 of wound healing, when the transplanted TAMEMs were barely detectable, we examined the number of macrophages in the wound and showed that neither the addition of TAMEMs nor the cocktail changed the number of macrophages (**Appendix Fig S6D**). We next examined changes in the macrophage phenotype at the wound on day 7 of wound healing, while adding two membrane surface markers as indicators of M2-like macrophages in addition to CD206, as suggested by the reviewer in Q2. As shown in new **Appendix Fig S6E**, the addition of TAMEMs at day 7 post injury increased the expression of these markers, which are deficient in diabetic mice during wound healing, and the addition of the cocktail did not reverse this deficiency. Finally, as we responded to Q2, we also examined the expression of *Il1b*, *Ptgs*, *Mgl2*, and *Clec10a* in macrophages at the wound site at day 7 of wound healing. As shown in the new **Appendix Fig S6F**, TAMEMs addition reduces the expression of *Il1b* and *Ptgs* in macrophages, which are highly expressed in diabetic wound macrophages, and increases the expression of *Mgl2* and *Clec10a* in macrophages, which are lowly expressed in diabetic wound macrophages, but the cocktail failed to do.

Minor points

Q4. Clarify the methods and the number of biological replication for the detection and quantification in figure 1C

A4: We have clarified the experimental method in the **revised Methods** and the number of biological replicates in the legend. There are 3 biological replicates in the original Fig. 1C, but as requested by reviewer 2, we repeated the experiment applying **6 biological replicates** and updated in the new **Fig 1C**.

Q5. Add representative genes for TAMEMs in figure 2E

A5: We have added the representative genes for TAMEMs in the updated **Fig 2E**.

Q6. The figure legend and figure title are different in Figure 5C. (Legend: monocytes, title: macrophages)

A6: We thank the reviewer for this reminder. We have corrected 'macrophages' in the figure title to 'monocytes'.

Q7. For figure 6B, a volcano plot with the number of up/down-regulated genes could be used to show the difference between TAMEM and TAMEMC-M

A7: This is a great suggestion. We have added a volcano plot showing the difference genes between TAMEMs and TAMEMs$^{C\text{-}m}$ in the new **Fig EV1J**. There were relatively few differentially expressed genes between TAMEMs and TAMEMs$^{C\text{-}m}$, including 237

up-regulated genes and 323 down-regulated genes, which is consistent with the results of the correlation analysis between these two macrophages (**Fig 6C**).

Q8. Some figures, especially microscope images are in low resolution.
A8: We have improved all images with this issue, such as **Fig 4G**, **Fig 5E**, **Fig 7D**, **Fig EV3A**, **Fig EV3B**, **Fig EV5D**, and **Appendix Fig S6B**.

Finally, we wish to thank all the three reviewers for their expert opinions and valuable suggestions, which have helped us greatly improve this work.

References for this letter of response

1. Wang, L.; Wang, C.; Wang, Z.; Gan, J.; Liu, C.; Xia, S.; Niu, Y.; Chen, D.; Zhang, J.; Dong, L., Transforming the spleen into a liver-like organ in vivo. *Sci Adv* **2020,** *6* (24), eaaz9974.
2. Wang, G. G.; Calvo, K. R.; Pasillas, M. P.; Sykes, D. B.; Häcker, H.; Kamps, M. P., Quantitative production of macrophages or neutrophils ex vivo using conditional Hoxb8. *Nat Methods* **2006,** *3* (4), 287-93.
3. Forssell, J.; Oberg, A.; Henriksson, M. L.; Stenling, R.; Jung, A.; Palmqvist, R., High macrophage infiltration along the tumor front correlates with improved survival in colon cancer. *Clin Cancer Res* **2007,** *13* (5), 1472-9.
4. Kimball, A.; Schaller, M.; Joshi, A.; Davis, F. M.; Dendekker, A.; Boniakowski, A.; Bermick, J.; Obi, A.; Moore, B.; Henke, P. K.; Kunkel, S. L.; Gallagher, K. A., Ly6C Hi Blood Monocyte/Macrophage Drive Chronic Inflammation and Impair Wound Healing in Diabetes Mellitus. *Arteriosclerosis, Thrombosis, and Vascular Biology* **2018,** *38* (5), 1102-1114.
5. Xie, Y.; Sakatsume, M.; Nishi, S.; Narita, I.; Arakawa, M.; Gejyo, F., Expression, roles, receptors, and regulation of osteopontin in the kidney. *Kidney Int* **2001,** *60* (5), 1645-57.
6. Rabenhorst, A.; Hartmann, K., Interleukin-31: a novel diagnostic marker of allergic diseases. *Curr Allergy Asthma Rep* **2014,** *14* (4), 423.
7. Mittl, P. R.; Priestle, J. P.; Cox, D. A.; McMaster, G.; Cerletti, N.; Grütter, M. G., The crystal structure of TGF-beta 3 and comparison to TGF-beta 2: implications for receptor binding. *Protein Sci* **1996,** *5* (7), 1261-71.
8. Ge, B.; Li, J.; Wei, Z.; Sun, T.; Song, Y.; Khan, N. U., Functional expression of CCL8 and its interaction with chemokine receptor CCR3. *BMC Immunol* **2017,** *18* (1), 54.
9. Lal, N.; Puri, K.; Rodrigues, B., Vascular Endothelial Growth Factor B and Its Signaling. *Front Cardiovasc Med* **2018,** *5*, 39.
10. Liekens, S.; Leali, D.; Neyts, J.; Esnouf, R.; Rusnati, M.; Dell'Era, P.; Maudgal, P. C.; De Clercq, E.; Presta, M., Modulation of fibroblast growth factor-2 receptor binding, signaling, and mitogenic activity by heparin-mimicking polysulfonated compounds. *Mol Pharmacol* **1999,** *56* (1), 204-13.

11. Tao, N.; Wagner, S. J.; Lublin, D. M., CD36 is palmitoylated on both N- and C-terminal cytoplasmic tails. *J Biol Chem* **1996,** *271* (37), 22315-20.
12. Kaufmann, A.; Salentin, R.; Gemsa, D.; Sprenger, H., Increase of CCR1 and CCR5 expression and enhanced functional response to MIP-1α during differentiation of human monocytes to macrophages. *Journal of Leukocyte Biology* **2001,** *69* (2), 248-252.
13. Wu, Z.; Harne, R.; Chintoan-Uta, C.; Hu, T.-J.; Wallace, R.; MacCallum, A.; Stevens, M. P.; Kaiser, P.; Balic, A.; Hume, D. A., Regulation and function of macrophage colony-stimulating factor (CSF1) in the chicken immune system. *Developmental & Comparative Immunology* **2020,** *105*, 103586.
14. Sato, Y.; Bando, H.; Di Piazza, M.; Gowing, G.; Herberts, C.; Jackman, S.; Leoni, G.; Libertini, S.; MacLachlan, T.; McBlane, J. W.; Pereira Mouriès, L.; Sharpe, M.; Shingleton, W.; Surmacz-Cordle, B.; Yamamoto, K.; van der Laan, J. W., Tumorigenicity assessment of cell therapy products: The need for global consensus and points to consider. *Cytotherapy* **2019,** *21* (11), 1095-1111.
15. Bai, H.; Wang, J.; Phan, C. U.; Chen, Q.; Hu, X.; Shao, G.; Zhou, J.; Lai, L.; Tang, G., Cyclodextrin-based host-guest complexes loaded with regorafenib for colorectal cancer treatment. *Nat Commun* **2021,** *12* (1), 759.
16. Popkov, M.; Jendreyko, N.; Gonzalez-Sapienza, G.; Mage, R. G.; Rader, C.; Barbas, C. F., 3rd, Human/mouse cross-reactive anti-VEGF receptor 2 recombinant antibodies selected from an immune b9 allotype rabbit antibody library. *J Immunol Methods* **2004,** *288* (1-2), 149-64.
17. Feng, Y.; Mu, R.; Wang, Z.; Xing, P.; Zhang, J.; Dong, L.; Wang, C., A toll-like receptor agonist mimicking microbial signal to generate tumor-suppressive macrophages. *Nat Commun* **2019,** *10* (1), 2272.
18. Locati, M.; Curtale, G.; Mantovani, A., Diversity, Mechanisms, and Significance of Macrophage Plasticity. *Annu Rev Pathol* **2020,** *15*, 123-147.
19. Hu, M. S.; Walmsley, G. G.; Barnes, L. A.; Weiskopf, K.; Rennert, R. C.; Duscher, D.; Januszyk, M.; Maan, Z. N.; Hong, W. X.; Cheung, A. T.; Leavitt, T.; Marshall, C. D.; Ransom, R. C.; Malhotra, S.; Moore, A. L.; Rajadas, J.; Lorenz, H. P.; Weissman, I. L.; Gurtner, G. C.; Longaker, M. T., Delivery of monocyte lineage cells in a biomimetic scaffold enhances tissue repair. *JCI Insight* **2017,** *2* (19).
20. Jayme, T. S.; Leung, G.; Wang, A.; Workentine, M. L.; Rajeev, S.; Shute, A.; Callejas, B. E.; Mancini, N.; Beck, P. L.; Panaccione, R.; McKay, D. M., Human interleukin-4-treated regulatory macrophages promote epithelial wound healing and reduce colitis in a mouse model. *Sci Adv* **2020,** *6* (23), eaba4376.
21. Peters, L. L.; Robledo, R. F.; Bult, C. J.; Churchill, G. A.; Paigen, B. J.; Svenson, K. L., The mouse as a model for human biology: a resource guide for complex trait analysis. *Nature Reviews Genetics* **2007,** *8* (1), 58-69.
22. Boniakowski, A. E.; Kimball, A. S.; Joshi, A.; Schaller, M.; Davis, F. M.; denDekker, A.; Obi, A. T.; Moore, B. B.; Kunkel, S. L.; Gallagher, K. A., Murine macrophage chemokine receptor CCR2 plays a crucial role in macrophage recruitment and regulated inflammation in wound healing. *Eur J Immunol* **2018,** *48* (9), 1445-1455.
23. Ferreira, D. W.; Ulecia-Morón, C.; Alvarado-Vázquez, P. A.; Cunnane, K.; Moracho-Vilriales, C.; Grosick, R. L.; Cunha, T. M.; Romero-Sandoval, E. A., CD163

overexpression using a macrophage-directed gene therapy approach improves wound healing in ex vivo and in vivo human skin models. *Immunobiology* **2020,** *225* (1), 151862.
24. Willenborg, S.; Sanin, D. E.; Jais, A.; Ding, X.; Ulas, T.; Nüchel, J.; Popović, M.; MacVicar, T.; Langer, T.; Schultze, J. L.; Gerbaulet, A.; Roers, A.; Pearce, E. J.; Brüning, J. C.; Trifunovic, A.; Eming, S. A., Mitochondrial metabolism coordinates stage-specific repair processes in macrophages during wound healing. *Cell Metabolism* **2021,** *33* (12), 2398-2414.e9.

28th Nov 2022

Dear Prof. Wang,

Thank you for the submission of your revised manuscript to EMBO Molecular Medicine. We have now received the enclosed report from the three referees who agreed to re-assess it. As you will see, the referees are now supportive, and I am pleased to inform you that we will be able to accept your manuscript pending the following amendments:

1. Please reduce the keyword number to 5.

2. Remove "data not shown". Per our guidelines, on "Unpublished Data," the journal does not permit citation of "Data not shown". All data referred to in the paper should be displayed in the main or Expanded View figures.

3. Remove the Author Contribution from the manuscript text.

4. Funding information entered in the online submission system should match that listed in the manuscript text. The current funding information is incomplete in the online submission system.

5. Reference format: Please list up to 10 co-authors of a paper before adding et al. to the reference list.

6. Please remove the two weblinks (Linkedin and lab webpage).

7. Appendix

- Remove the blue color font.
- Supplementary methods should be deleted from the appendix and merged into the Material and Methods section in the main manuscript. Update the table of content accordingly.

8. Figure 3C
- Please clarify whether the large and small images(F4/80) are derived from the same cells. In the two M1-CM panels, these images do not seem to match.

9. The Scheme illustration should be uploaded and called out as Figure 8. Move the figure legend after the current Figure 7.

10. Every published paper includes a 'Synopsis' to further enhance discoverability. Synopses are displayed on the journal webpage and are freely accessible to all readers. They include a short stand first (maximum of 300 characters, including space) as well as 2-5 one-sentences bullet points that summarizes the paper. Please write the bullet points to summarize the key NEW findings. They should be designed to be complementary to the abstract - i.e. not repeat the same text. We encourage the inclusion of key acronyms and quantitative information (maximum of 30 words / bullet point). Please use the passive voice. Please attach these in a separate file or send them by email, we will incorporate them accordingly.

Please also provide a striking image or visual abstract to illustrate your article as a PNG file 550 px wide x 300-600 px high.

11. Our data editors have seen the manuscript, and they have made some comments and suggestions that need to be addressed (see attached). Please send back a revised version (in track change mode), as we will need to go through the changes.

12. As part of the EMBO Publications transparent editorial process initiative (see our Editorial at http://embomolmed.embopress.org/content/2/9/329), EMBO Molecular Medicine will publish online a Review Process File (RPF) to accompany accepted manuscripts.

In the event of acceptance, this file will be published in conjunction with your paper and will include the anonymous referee reports, your point-by-point response, and all pertinent correspondence relating to the manuscript. Let us know whether you disagree with this and if you want to remove or keep any figures from it prior to publication.

Please note that the Authors checklist will be published at the end of the RPF.

I look forward to seeing a revised form of your manuscript as soon as possible.

Sincerely,
Jingyi

Jingyi Hou
Editor
EMBO Molecular Medicine

***** Reviewer's comments *****

Referee #1 (Remarks for Author):

The authors have very well responded to my criticisms, and the revised manuscript is significantly improved.

Referee #2 (Comments on Novelty/Model System for Author):

With increased replicates, the results are now much more credible and impactful.

Referee #3 (Comments on Novelty/Model System for Author):

No issues with the models used.

Referee #3 (Remarks for Author):

All my previous comments have been addressed satisfactorily.

The authors addressed the minor editorial issues.

2nd Dec 2022

Dear Prof. Wang,

We are pleased to inform you that your manuscript is accepted for publication and is now being sent to our publisher to be included in the next available issue of EMBO Molecular Medicine.

We would like to remind you that as part of the EMBO Publications transparent editorial process initiative, EMBO Molecular Medicine will publish a Review Process File online to accompany accepted manuscripts. If you do NOT want the file to be published or would like to exclude figures, please immediately inform the editorial office via e-mail.

Please read below for additional IMPORTANT information regarding your article, its publication and the production process.

Congratulations on your interesting work,

Jingyi

Jingyi Hou
Editor
EMBO Molecular Medicine

Follow us on Twitter @EmboMolMed
Sign up for eTOCs at embopress.org/alertsfeeds

EMBO Press Author Checklist

Corresponding Author Name: Chunming Wang, Lei Dong	
Journal Submitted to: EMBO Molecular Medicine	
Manuscript Number: EMM-2022-16671-T	

USEFUL LINKS FOR COMPLETING THIS FORM
The EMBO Journal - Author Guidelines
EMBO Reports - Author Guidelines
Molecular Systems Biology - Author Guidelines
EMBO Molecular Medicine - Author Guidelines

Reporting Checklist for Life Science Articles (updated January 2022)
This checklist is adapted from Materials Design Analysis Reporting (MDAR) Checklist for Authors. MDAR establishes a minimum set of requirements in transparent reporting in the life sciences (see Statement of Task: 10.31222/osf.io/9sm4x). Please follow the journal's guidelines in preparing your manuscript.
Please note that a copy of this checklist will be published alongside your article.

Abridged guidelines for figures
1. Data
The data shown in figures should satisfy the following conditions:
- the data were obtained and processed according to the field's best practice and are presented to reflect the results of the experiments in an accurate and unbiased manner.
- ideally, figure panels should include only measurements that are directly comparable to each other and obtained with the same assay.
- plots include clearly labeled error bars for independent experiments and sample sizes. Unless justified, error bars should not be shown for technical replicates.
- if n<5, the individual data points from each experiment should be plotted. Any statistical test employed should be justified.
- Source Data should be included to report the data underlying figures according to the guidelines set out in the authorship guidelines on Data Presentation.

2. Captions
Each figure caption should contain the following information, for each panel where they are relevant:
- a specification of the experimental system investigated (eg cell line, species name).
- the assay(s) and method(s) used to carry out the reported observations and measurements.
- an explicit mention of the biological and chemical entity(ies) that are being measured.
- an explicit mention of the biological and chemical entity(ies) that are altered/varied/perturbed in a controlled manner.
- the exact sample size (n) for each experimental group/condition, given as a number, not a range;
- a description of the sample collection allowing the reader to understand whether the samples represent technical or biological replicates (including how many animals, litters, cultures, etc.).
- a statement of how many times the experiment shown was independently replicated in the laboratory.
- definitions of statistical methods and measures:
  - common tests, such as t-test (please specify whether paired vs. unpaired), simple χ2 tests, Wilcoxon and Mann-Whitney tests, can be unambiguously identified by name only, but more complex techniques should be described in the methods section;
  - are tests one-sided or two-sided?
  - are there adjustments for multiple comparisons?
  - exact statistical test results, e.g., P values = x but not P values < x;
  - definition of 'center values' as median or average;
  - definition of error bars as s.d. or s.e.m.

Please complete ALL of the questions below.
Select "Not Applicable" only when the requested information is not relevant for your study.

Materials

Newly Created Materials	Information included in the manuscript?	In which section is the information available? (Reagents and Tools Table, Materials and Methods, Figures, Data Availability Section)
New materials and reagents need to be available; do any restrictions apply?	Not Applicable	

Antibodies	Information included in the manuscript?	In which section is the information available? (Reagents and Tools Table, Materials and Methods, Figures, Data Availability Section)
For **antibodies** provide the following information:<br>- Commercial antibodies: RRID (if possible) or supplier name, catalogue number and or/clone number<br>- Non-commercial: RRID or citation	Yes	Appendix Table S2

DNA and RNA sequences	Information included in the manuscript?	In which section is the information available? (Reagents and Tools Table, Materials and Methods, Figures, Data Availability Section)
**Short novel DNA or RNA including primers, probes**: provide the sequences.	Yes	Appendix Table S1

Cell materials	Information included in the manuscript?	In which section is the information available? (Reagents and Tools Table, Materials and Methods, Figures, Data Availability Section)
**Cell lines:** Provide species information, strain. Provide accession number in repository **OR** supplier name, catalog number, clone number, and/**OR** RRID.	Yes	Materials and Methods
**Primary cultures:** Provide species, strain, sex of origin, genetic modification status.	Yes	Materials and Methods
Report if the cell lines were recently **authenticated** (e.g., by STR profiling) and tested for mycoplasma contamination.	Not Applicable	

Experimental animals	Information included in the manuscript?	In which section is the information available? (Reagents and Tools Table, Materials and Methods, Figures, Data Availability Section)
**Laboratory animals or Model organisms:** Provide species, strain, sex, age, genetic modification status. Provide accession number in repository **OR** supplier name, catalog number, clone number, **OR** RRID.	Yes	Materials and Methods
**Animal observed in or captured from the field:** Provide species, sex, and age where possible.	Not Applicable	
Please detail **housing and husbandry conditions**.	Yes	Materials and Methods

Plants and microbes	Information included in the manuscript?	In which section is the information available? (Reagents and Tools Table, Materials and Methods, Figures, Data Availability Section)
**Plants:** provide species and strain, ecotype and cultivar where relevant, unique accession number if available, and source (including location for collected wild specimens).	Not Applicable	
**Microbes:** provide species and strain, unique accession number if available, and source.	Not Applicable	

Human research participants	Information included in the manuscript?	In which section is the information available? (Reagents and Tools Table, Materials and Methods, Figures, Data Availability Section)
If collected and within the bounds of privacy constraints report on age, sex and gender or ethnicity for all study participants.	Not Applicable	

Core facilities	Information included in the manuscript?	In which section is the information available? (Reagents and Tools Table, Materials and Methods, Figures, Data Availability Section)
If your work benefited from core facilities, was their service mentioned in the acknowledgments section?	Not Applicable	

Design

Study protocol	Information included in the manuscript?	In which section is the information available? (Reagents and Tools Table, Materials and Methods, Figures, Data Availability Section)
If study protocol has been **pre-registered, provide DOI in the manuscript**. For clinical trials, provide the trial registration number **OR** cite DOI.	Not Applicable	
Report the **clinical trial registration number** (at ClinicalTrials.gov or equivalent), where applicable.	Not Applicable	

Laboratory protocol	Information included in the manuscript?	In which section is the information available? (Reagents and Tools Table, Materials and Methods, Figures, Data Availability Section)
Provide DOI OR other citation details if **external detailed step-by-step protocols** are available.	Yes	Materials and Methods

Experimental study design and statistics	Information included in the manuscript?	In which section is the information available? (Reagents and Tools Table, Materials and Methods, Figures, Data Availability Section)
Include a statement about **sample size** estimate even if no statistical methods were used.	Yes	Materials and Methods; Figure legends
Were any steps taken to minimize the effects of subjective bias when allocating animals/samples to treatment (e.g. **randomization procedure**)? If yes, have they been described?	Yes	Materials and Methods
Include a statement about **blinding** even if no blinding was done.	Yes	Materials and Methods
Describe **inclusion/exclusion criteria** if samples or animals were excluded from the analysis. Were the criteria pre-established?<br><br>If sample or data points were omitted from analysis, report if this was due to attrition or intentional exclusion and provide justification.	Not Applicable	
For every figure, are **statistical tests** justified as appropriate? Do the data meet the assumptions of the tests (e.g., normal distribution)? Describe any methods used to assess it. Is there an estimate of variation within each group of data? Is the variance similar between the groups that are being statistically compared?	Yes	Normal distribution was tested by Shapiro-Wilk normality test

Sample definition and in-laboratory replication	Information included in the manuscript?	In which section is the information available? (Reagents and Tools Table, Materials and Methods, Figures, Data Availability Section)
In the figure legends: state number of times the experiment was **replicated** in laboratory.	Yes	Figure legends
In the figure legends: define whether data describe **technical or biological replicates**.	Yes	Figure legends

Ethics

Ethics	Information included in the manuscript?	In which section is the information available? (Reagents and Tools Table, Materials and Methods, Figures, Data Availability Section)
Studies involving **human participants**: State details of **authority granting ethics approval** (IRB or equivalent committee(s), provide reference number for approval.	Not Applicable	
Studies involving **human participants**: Include a statement confirming that **informed consent** was obtained from all subjects and that the experiments conformed to the principles set out in the WMA Declaration of Helsinki and the Department of Health and Human Services Belmont Report.	Not Applicable	
Studies involving **human participants:** For publication of **patient photos**, include a statement confirming that consent to publish was obtained.	Not Applicable	
Studies involving experimental **animals**: State details of **authority granting ethics approval** (IRB or equivalent committee(s), provide reference number for approval. Include a statement of compliance with ethical regulations.	Yes	Materials and Methods
Studies involving **specimen and field samples:** State if relevant **permits** obtained, provide details of authority approving study; if none were required, explain why.	Not Applicable	

Dual Use Research of Concern (DURC)	Information included in the manuscript?	In which section is the information available? (Reagents and Tools Table, Materials and Methods, Figures, Data Availability Section)
Could your study fall under dual use research restrictions? Please check biosecurity documents and list of **select agents and toxins** (CDC): https://www.selectagents.gov/sat/list.htm	Not Applicable	
If you used a select agent, is the security level of the lab appropriate and reported in the manuscript?	Not Applicable	
If a study is subject to dual use research of concern regulations, is the name of the **authority granting approval and reference number** for the regulatory approval provided in the manuscript?	Not Applicable	

Reporting

The MDAR framework recommends adoption of discipline-specific guidelines, established and endorsed through community initiatives. Journals have their own policy about requiring specific guidelines and recommendations to complement MDAR.

Adherence to community standards	Information included in the manuscript?	In which section is the information available? (Reagents and Tools Table, Materials and Methods, Figures, Data Availability Section)
State if relevant guidelines or checklists (e.g., **ICMJE, MIBBI, ARRIVE, PRISMA**) have been followed or provided.	Not Applicable	
For **tumor marker prognostic studies,** we recommend that you follow the **REMARK** reporting guidelines (see link list at top right). See author guidelines, under 'Reporting Guidelines'. Please confirm you have followed these guidelines.	Not Applicable	
For **phase II and III randomized controlled trials**, please refer to the **CONSORT** flow diagram (see link list at top right) and submit the CONSORT checklist (see link list at top right) with your submission. See author guidelines, under 'Reporting Guidelines'. Please confirm you have submitted this list.	Not Applicable	

Data Availability

Data availability	Information included in the manuscript?	In which section is the information available? (Reagents and Tools Table, Materials and Methods, Figures, Data Availability Section)
Have **primary datasets** been deposited according to the journal's guidelines (see 'Data Deposition' section) and the respective accession numbers provided in the Data Availability Section?	Yes	Data Availability Section
Were **human clinical and genomic datasets** deposited in a public access-controlled repository in accordance to ethical obligations to the patients and to the applicable consent agreement?	Not Applicable	
Are **computational models** that are central and integral to a study available without restrictions in a machine-readable form? Were the relevant accession numbers or links provided?	Not Applicable	
If publicly available data were reused, provide the respective **data citations in the reference list**.	Not Applicable	
